# Dual Agents: Fungal Macrocidins and Synthetic Analogues with Herbicidal and Antibiofilm Activities

**DOI:** 10.3390/antibiotics10081022

**Published:** 2021-08-23

**Authors:** Laura Treiber, Christine Pezolt, Haoxuan Zeng, Hedda Schrey, Stefan Jungwirth, Aditya Shekhar, Marc Stadler, Ursula Bilitewski, Maike Erb-Brinkmann, Rainer Schobert

**Affiliations:** 1 Department of Chemistry, University Bayreuth, Universitaetsstr. 30, 95440 Bayreuth, Germany; laura1.treiber@uni-bayreuth.de (L.T.); christine.pezolt@uni-bayreuth.de (C.P.); 2 Department of Microbial Drugs, Helmholtz Centre for Infection Research GmbH, Inhoffenstrasse 7, 38124 Braunschweig, Germany; haoxuan.zeng@helmholtz-hzi.de (H.Z.); hedda.schrey@helmholtz-hzi.de (H.S.); marc.stadler@helmholtz-hzi.de (M.S.); 3 Institute of Microbiology, Technische Universität Braunschweig, Spielmannstraße 7, 38106 Braunschweig, Germany; 4 Compound Profiling and Screening, Helmholtz Centre for Infection Research GmbH, Inhoffenstrasse 7, 38124 Braunschweig, Germany; stefan.Jungwirth@stud.uni-regensburg.de (S.J.); aditya.shekhar@helmholtz-hzi.de (A.S.); ursula.bilitewski@helmholtz-hzi.de (U.B.); 5 Phytosolution, Querfurter Strasse 9, 06632 Freyburg, Germany; m.erb-brinkmann@phytosolution.de

**Keywords:** macrocidin, polycyclic tetramate macrolactams, 3-acyltetramic acids, antibiotics, biofilms

## Abstract

Eight analogues of the bioherbicides macrocidin A (**1**) and Z (**2**) with structural variance in the size of the macrocycle, its *para*- or *meta*-cyclophane character, and its functional groups were synthesized on two modular routes and tested for herbicidal, antibiotic, and antibiofilm activities. Apart from the lead compounds **1** and **2**, the structurally simplified dihydromacrocidin Z (**3**) and normacrocidin Z (**4**) showed high herbicidal activity in either thistles, dandelions or in both. The derivatives **2**, **3**, and dibromide **9** also inhibited the growth of *Staphylococcus aureus* biofilms by ca 70% when applied at subtoxic concentrations as low as ca 20 µM, which are unlikely to induce bacterial resistance. They also led to the dispersion of preformed biofilms of *S. aureus*, exceeding a similar effect by microporenic acid A, a known biofilm inhibitor. Compounds **3** and **9** showed no noticeable cytotoxicity against human cancer and endothelial cells at concentrations below 50 µM, making them conceivable candidates for application as anti-biofilm agents in a medicinal context.

## 1. Introduction

**Figure 1:**
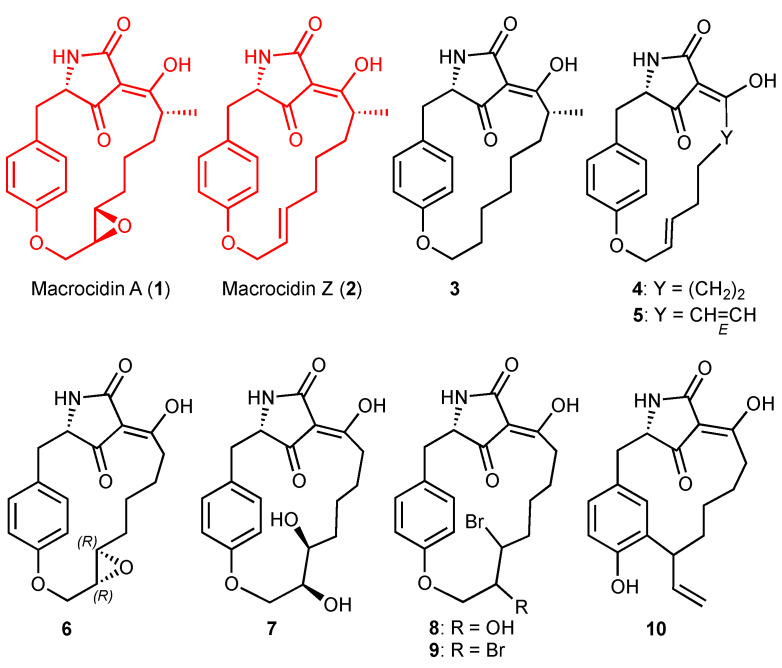
Structures of the natural lead compounds macrocidin A (**1**) and macrocidin Z (**2**) and structural variants **3**–**10**.

Macrocidins are polycyclic tetramic acid macrolactams (PTMs). Macrocidin A (**1**; Figure 1) was first isolated from the fungus *Phoma macrostoma* Montagne in 2003 by a Dow AgroSciences group headed by Graupner [1]. It was found to induce chlorosis in broadleaf weeds by a unique mode of action implying an interference with the phytoene synthase and desaturase in the chlorophyll and carotenoid biosynthesis [2]. So far, only two total syntheses of macrocidin A (**1**) have been published [3,4]. Macrocidin Z (**2**), which carries an *E*-alkene in lieu of the expoxide, was isolated from *Phoma macrostoma* cultures and synthesised in parallel by us only recently [5]. Although concentrated *Phoma macrostoma* cultures, formulated as broadcast granules, are being used as bioherbicides for environment-friendly weed management in the US and Canada, efforts towards the synthesis of simplified macrocidin derivatives with improved herbicidal properties were sporadically made (e.g., by Graupner’s group [6] and Syngenta [7]), albeit without disclosing details. As 3-acyltetramic acids from a broad range of sources were found to have antibiotic or biofilm inhibitory effects [8,9,10,11,12] (e.g., by us in the case of macrocidin Z (**2**) [5]), we now synthesised analogues of the natural macrocidins with variation of the structural key features such as the size of the macrocycle, its *para*- or *meta*-cyclophane character, and its decoration with functional groups other than epoxide. We tested them for herbicidal, antibacterial, and antibiofilm activities, and for cytotoxicity to human cells.

## 2. Results

### 2.1. Chemistry

Figure 1 depicts eight new derivatives **3**–**10** that were prepared on two efficient modular routes. They share the same tetramic acid derived from l-tyrosine, yet differ in their degree of resemblance to the lead macrocidins (**1**) and (**2**). The dihydro derivate **3** of macrocidin Z is the only one retaining the methyl group at an *R*-configured stereocenter. Like macrocidin Z (**2**), the derivatives **4** and **5** feature *E*-alkenes in lieu of the epoxide. Normacrocidin A (**6**) lacks the methyl group while retaining the epoxide, albeit with a non-natural *R*,*R* configuration. In compound **7**, the epoxide is replaced by a vicinal diol, in derivative **8** by a bromohydrin, and in **9** by a vicinal dibromide. Furthest from the natural leads **1** and **2** (in terms of structure) is derivative **10,** which has a 13-membered instead of a 17-membered macrocycle comprising a *meta*- rather than a *para*-cyclophane.

**Scheme 1:**
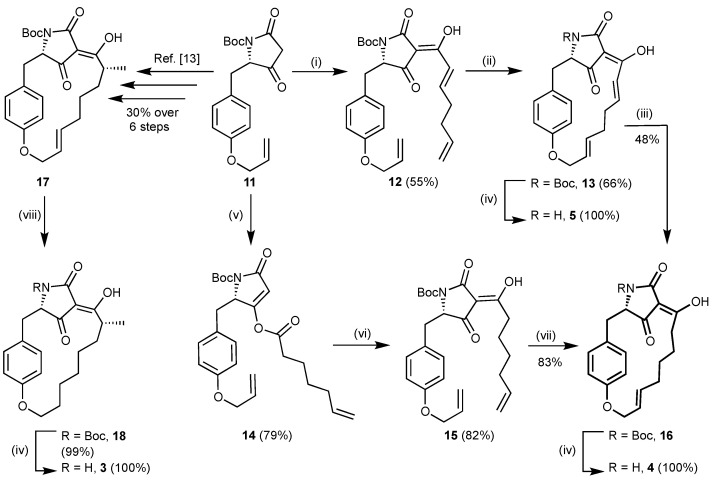
Syntheses of key intermediate **16** and of macrocidin derivatives **3**, **4**, and **5**. Reagents and conditions: (i) Ph_3_PCCO, THF, reflux, 2 h; then KO*t*Bu, THF, reflux, 20 min; then 4-pentenal, THF, reflux → rt, 21 h; (ii) Grubbs II catalyst, CH_2_Cl_2_, reflux, 18 h; (iii) Rh(PPh_3_)_3_Cl, Et_3_SiH, CH_2_Cl_2_, reflux, 19 h; then KF, MeOH, −15 °C, 27 h; (iv) TFA, CH_2_Cl_2_, rt, 15 min; (v) EDC HCl, DMAP, 6-heptenoic acid, CH_2_Cl_2_, 0 °C → rt, 4 h; (vi) NEt_3_, DMAP, CH_2_Cl_2_, rt, 24 h; (vii) Grubbs II catalyst, CH_2_Cl_2_, reflux, 24 h; (viii) H_2_ (1 atm), Pd/C, EtOAc, rt, 31 h.

In Scheme 1, two alternative synthetic approaches to key intermediate **16**, an *N*-Boc-protected normacrocidin Z, are illustrated. The known l-tyrosine derived tetramic acid **11 [4]** was furnished with a 3-(hepta-2,6-dienoyl) residue by a one-pot reaction first with ketenylidentriphenylphosphorane, Ph_3_PCCO, to give the corresponding 3-acyl ylide (not shown), followed by a Wittig olefination of the latter with 4-pentenal to leave 3-acyltetramic acid **12** in 55% yield [13]. An *E*-selective ring-closing metathesis reaction with a Grubbs type-II catalyst gave macrocycle **13** in 66% yield as a single stereoisomer. Its reduction with Wilkinson’s catalyst and triethylsilane to afford a silyl enol ether (not shown), followed by its cleavage with KF, gave *N*-Boc-protected normacrocidin Z **16** in a 48% yield. Removal of the *N*-Boc-protecting group from compounds **16** and **13** using TFA left test candidates **4** and **5** as pure stereoisomers. For the synthesis of larger quantities of **16**, another route was developed also starting from tetramic acid **11** in analogy to our syntheses of macrocidins A and Z [4,5]. 3-Acyltetramic acid **15** was built up by 4-*O*-acylation of **11** with 6-heptenoic acid and subsequent rearrangement of tetramate **14**. Ring-closing metathesis of **15** with Grubbs type-II catalyst afforded key intermediate **16** in 54% total yield over three steps. Enantiopure dihydromacrocidin Z (**3**) was obtained from *N*-Boc-protected macrocidin Z **17** by hydrogenation and removal of the *N*-Boc-protecting group from **18** in 99% yield over two steps [5].

**Scheme 2:**
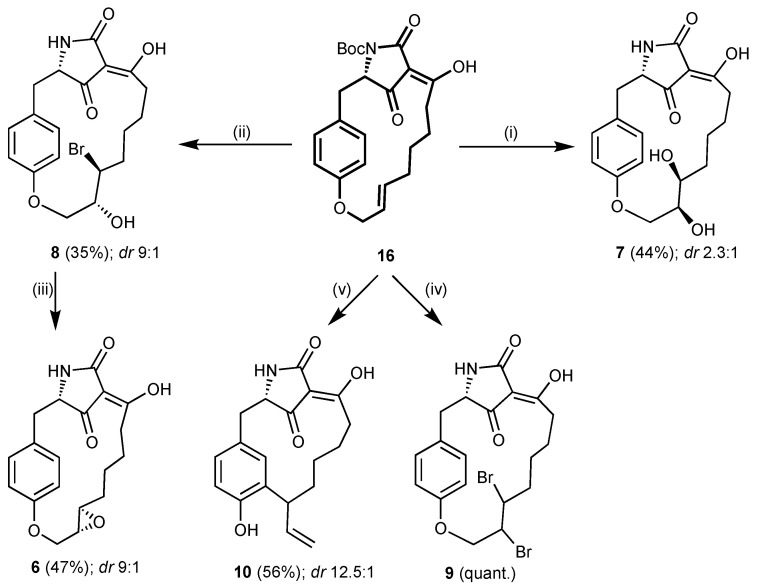
Modular synthesis of macrocidin derivatives **6**–**10** starting from key intermediate **16**. Reagents and conditions: (i) AD-mix α, *t*BuOH/H_2_O, 7 °C, 9 d; then TFA, CH_2_Cl_2_, rt, 15 min; (ii) NBS, H_2_O, DMSO, 8 °C → rt, 22 h; then TFA, CH_2_Cl_2_, rt, 15 min; (iii) KO*t*Bu, THF, 0 °C → rt, 4 d; (iv) Br_2_, CCl_4_, 80 °C, 30 h; (v) diethylaniline, sealed tube, 190 °C, 42 h.

Key intermediate **16** was also used to introduce further functionalizations, formally replacing the epoxide in macrocidin A (**1**). Its hydroxylation with AD-mix α afforded, after deprotection, diol **7** as an inseparable 2.3:1 mixture of two diastereomers (Scheme 2 shows major diastereomer). Alkene **16** was also converted to an inseparable mixture of two diastereomeric bromohydrins **8** with NBS and H_2_O in DMSO [14,15]. A side product (16%) of this reaction, carrying an additional bromo residue next to the enol group, could be separated. Upon treatment with KO*t*Bu, the bromohydrins **8** were converted in 47% yield to a 9:1 mixture of expoxides **6** as indicated by ^1^H NMR spectra. The configuration of the major isomer of **6** (shown in Scheme 2) was assigned by comparison with a mixture of isomers **6** obtained on a different route. Hence, we assume that the *dr* of precursor bromohydrins **8** was also 9:1 as for the epoxides **6**. After futile attempts by Ramana et al. [16] and our group at direct epoxidation of alkenes such as **4**, **16**, or **17**, this was the first time the epoxide function could be installed in the preformed macrocycle of a macrocidin precursor. Alkene **16** could also be brominated with bromine in CCl_4_ to give vicinal dibromide **9** as a mixture of two diastereomers with *trans*-positioned bromo residues as to NMR spectra. Finally, when heated in diethylaniline in a sealed tube, the *para*-cyclophane **16** underwent a Claisen rearrangement to afford *meta*-cyclophane **10** in 56% yield and as a 12.5:1 mixture of diastereomers according to NMR spectra. Like the stereopure derivatives **3**–**5**, the diastereomeric mixtures of derivatives **6**–**10** were tested for bioactivity, although in the case of diol **7** the two diastereomers, present in similar proportions, might dilute or cancel each other out in terms of biological activities. It should be noted, that most derivatives shown in Scheme 1 and Scheme 2 were difficult to purify and analyze. Sometimes, only multiple reversed-phase column chromatography runs led to the desired degree of purity. Their NMR spectra (*cf*. Supporting Information, SI) are rather complex due to the tautomerization of the 3-acyltetramic acid moiety [17].

### 2.2. Herbicidal Activity

**Figure 2:**
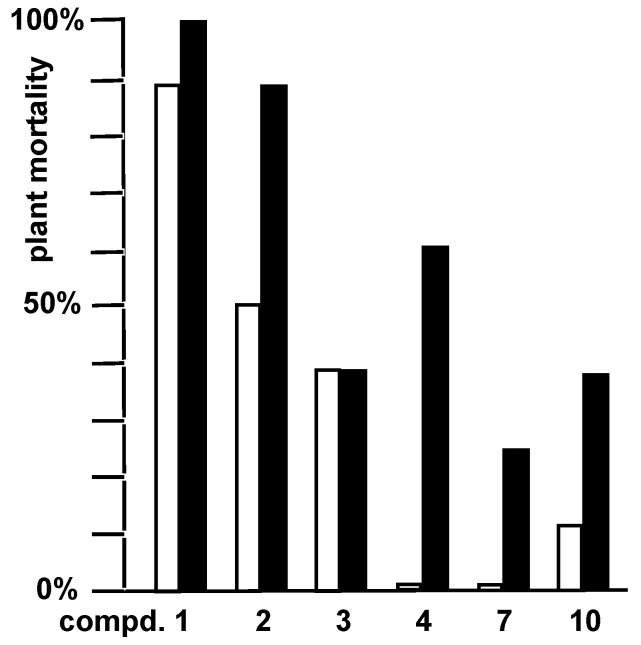
Percentage of final mortality of dandelions (white columns) and thistles (black columns) treated with 0.2 mL/plant of 100–150 mM solutions of active macrocidin derivatives after two to six weeks. Mortality by diflufenican was 100% for either plant species.

Prior to screening them for antimicrobial effects, the new derivatives were tested for herbicidal activity against thistles and dandelions which had been found to be susceptible to the chlorosis-inducing natural macrocidins [2,6]. For both species, they were applied as max. 150 mM solutions to four pots with two plants each, and their bleaching, withering, and necrotizing effects were assessed after two and then after three to six weeks. None of the compounds reached the efficiency of the synthetic commercial herbicide diflufenican, which was used as a positive control. In line with literature, the lead compound macrocidin A (**1**) exhibited the highest maximum herbicidal efficiency of all tested compounds, causing 88% mortality of dandelions and 100% of thistles, three weeks after application of a 100 mM solution in a mixture of isopropanol/water = 1:1 + 0.25% Tween 20 (Figure 2). Interestingly, the epoxide appeared not to be crucial for herbicidal activity, since macrocidin Z (**2**) still displayed a high efficiency of 88% mortality in thistles and of 50% in dandelions after 42 days at 100 mM. Contrary to an earlier assumption by Graupner, Bailey et al. [6], even derivatives with saturated backbones may show herbicidal efficiency, e.g., dihydromacrocidin Z (**3**) (38% mortality in thistles and dandelions). The α-methyl group seemed to be important, apparent from the lower figures for normacrocidin Z (**4**) when compared to **2** (dandelions: 0%, thistles: 63% mortality, 35 days after treatment with 150 mM) and for *S*,*R*,*R*-normacrocidin A (**6**) which was virtually inactive against both plants. The 13-membered macrocyclic *meta*-cyclophane **10** exerted a maximum herbicidal efficiency with 38% mortality in thistles, yet only 13% in dandelions after four weeks at 150 mM. Normacrocidin Z (**4**) and diol **7** also displayed a distinct specificity for thistles over dandelions (for pictures of treated plants *cf*. Figure S68 in the SI).

### 2.3. Antimicrobial Activity

As 3-acyltetramic acids were frequently shown to have antimicrobial effects [8,9,12,18,19,20], the new macrocidin derivatives were tested for activity against three different bacteria, namely the Gram-positive strain *Staphylococcus aureus* (SH1000) and the Gram-negative strains *Acinetobacter baumannii*, *Escherichia coli* with the wild-type strain K12 and the ΔTolC mutant (JW5503), which lacks the AcrAB−TolC efflux system. None of the macrocidin analogues displayed activity against the wild-type strain of *E. coli*. Weak activities against *E. coli* ΔTolC were found only for dibromide **9** (IC_50_ = 75 ± 15 µM), dihydromacrocidin Z (**3**) (IC_50_ = 82 ± 15 µM), and macrocidin Z (**2**) (IC_50_ ca 100 µM). These derivatives were also similarly active against *S. aureus* (*cf*. Table S1 for IC_50_ values and Figures S69–S70 for growth curves in the SI). In comparison, the clinically established antibiotic vancomycin was active with an IC_50_ of ca 12 µM against *S. aureus*, and the antibiotic erythromycin with a nanomolar IC_50_ value. None of the macrocidin derivatives showed a clear antibiotic effect on *A. baumannii*. Even when applied at the highest concentration of 100 µM, the compounds could not prevent the cultures from reaching an OD_600_ of at least 60–70% of the maximum value (*cf*. Figure S71 in the SI). Overall, the toxicity of macrocidin Z (**2**) and its new synthetic analogues **3**–**10** against bacteria is weak.

### 2.4. Antibiofilm Activity

**Figure 3:**
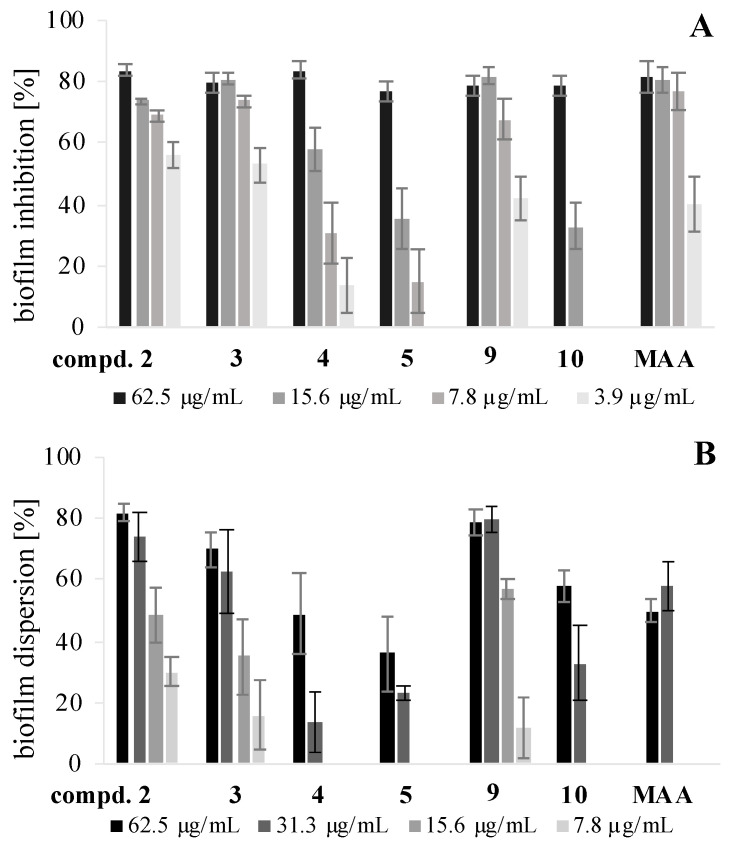
Effects of various concentrations of compounds **2**–**5**, **9**, and **10** on the formation of (**A**) and the dispersion of preformed (**B**) biofilms of *S. aureus*; positive control: microporenic acid A (MAA); error bars indicate SD of two repeats with duplicates.

The macrocidinoids **2**–**10** were tested for inhibitory effects on the formation of biofilms by *Staphylococcus aureus* and *Pseudomonas aeruginosa* bacteria, as well as for dispersive effects on preformed biofilms of *S. aureus* and the fungal species *Candida albicans* (*cf*. SI for data Table S2). While all compounds **2**–**6** and **8**–**10** inhibited the formation of *S. aureus* biofilms by at least 75% relative to untreated controls (=0%) at the highest tested concentration of 250 µg/mL, only macrocidin Z (**2**), its dihydro derivative **3** and dibromide **9** caused a distinct biofilm inhibition of ca 70% when applied at subtoxic concentrations as low as 7.8 µg/mL (corresponding to 16 µM and 23 µM, respectively). These activities matched or even exceeded that of microporenic acid A (MAA), the known biofilm inhibitor [21] used as a positive control (Figure 3A). A second group of moderately active inhibitors, comprising normacrocidin Z (**4**), diene **5** and phenol **10**, led to an inhibition of biofilm formation of more than 30% when applied at a concentration of 15.6 µg/mL (corresponding to 43–48 µM). The derivatives **7** and **8** had little inhibitory effect at concentrations below 250 µg/mL.

The dispersive effects on preformed biofilms of *S. aureus* were generally slightly less pronounced (Figure 3B). Derivatives **6**–**8** were inactive at all concentrations up to 250 µg/mL. The most distinct effects of at least 35% dispersion over the concentration range from 250 µg/mL down to 15.6 µg/mL were again observed for derivatives **2**, **3,** and **9**, which clearly outperformed MAA. The latter was active only at the highest two concentrations, such as the compounds **4**, **5,** and **10**. In the case of *C. albicans*, compounds **7** and **10** proved inactive, whereas the other compounds, including MAA, showed weak dispersive effects at very high concentrations (250–125 µg/mL) with derivate **5** being the only one active at a lower concentration of 62.5 µg/mL (192 µM) (*cf*. Figure S72 in the SI). None of the tested derivatives inhibited the formation of *P. aeruginosa* biofilms.

This study demonstrated that macrocidin analogues may interfere with the formation and persistence of bacterial and fungal biofilms, depending on their structure and polarity. The strongest effects against *S. aureus* were found for the lipophilic and structurewise “simple” derivatives **2**–**5** and **9**. Interestingly, the activity against biofilms decreased, or even disappeared, when hydroxy groups were introduced into the molecule, as the derivatives **7** and **8** exemplify.

### 2.5. Cytotoxicity

The macrocidinoids **2**, **3**, and **6**–**10** were submitted to provisional MTT tests for cytotoxicity/antiproliferative effects against human 518A2 melanoma cells, colon carcinoma cells HCT-116^wt^ and HCT-116^p53−/−^, and KBV cervix carcinoma cells as well as hybrid endothelial EaHy cells. Gratifyingly, none of the compounds but **2** caused signs of toxicity or inhibition of proliferation in the tested cells when applied at concentrations as high as 50 µM (*cf*. Table S3 in the SI). This is far outside of any clinically relevant range and would bode well for a potential future application as biofilm interfering agents in a medicinal context. Even macrocidin Z (**2**) which was antiproliferative with IC_50_ concentrations of ca. 15 to 30 µM in cells of colon carcinoma HCT-116 and cervical carcinoma KBV warrants a more in-depth study of its applicability as an anti-biofilm agent.

## 3. Discussion

We synthesised eight derivatives of the natural tetramic acids macrocidin A (**1**) and Z (**2**) on two efficient modular routes, which allow the introduction of various functionalities and scaffold modifications on a few key intermediates. The double bonds of intermediates **13** and **16** were converted in good yields to bromides, diols, bromohydrins and saturated bonds using standard reactions. A thermal Claisen rearrangement opened an easy access to a 13-membered macrocycle featuring a *meta*-cyclophane motif. For the first time, we could introduce the epoxide in a macrocidin precursor with a preformed macrocycle. The derivatives and the natural lead compound macrocidin Z (**2**) were tested for herbicidal and antimicrobial activity, as well as for biofilm interference. The incentive for this extended bioscreening were the frequent reports on the high incidence of antimicrobial and antifungal effects by 3-acyltetramic acids in general. Interestingly, the structurally simple compounds macrocidin Z (**2**) and dihydromacrocidin Z (**3**) showed a high herbicidal *and* antibiofilm activity. Normacrocidin Z (**4**) was selectively herbicidal against thistles, and dibromide **9** displayed an *S. aureus* biofilm dispersing effect surpassing even that of the known biofilm inhibitor microporenic acid A. With the exception of diol **7**, which was moderately herbicidal against thistles, hydroxy groups on the alkyl backbone of the macrocycle, appear to be generally detrimental to both herbicidal and antibiofilm effects. Contrary to suppositions in the scant literature on macrocidinoids, the epoxide function is obviously not crucial to either activity. The observed distinct and strain-specific effects of the active macrocidinoids **2**, **3**, and **9** on the biofilms of *S. aureus* are all the more interesting, as their direct antibacterial activities (i.e., toxicities against bacteria) are rather limited. Thus, their application as biofilm inhibitors would probably not induce bacterial resistance. The at best marginal cytotoxicities of compounds **3** and **9** in human cancer and endothelial cells indicate that these compounds would presumably be well tolerated also by higher organisms. Compound **2** might indeed pose a toxicity problem which should be clarified prior to further tests as a biofilm inhibitor.

## 4. Materials and Methods

### 4.1. General Information

IR spectra were recorded with a PerkinElmer Spectrum 100 FT-IR spectrophotometer (PerkinElmer, Rodgau, Germany) with ATR sampling unit. Optical rotations were measured at 589 nm (Na-D line) on a PerkinElmer 241 polarimeter (PerkinElmer, Rodgau, Germany); [α]_D_ values are given in 10^−1^ deg cm^2^ g^−1^. High resolution mass spectra were obtained with a UPLC/Orbitrap MS system in ESI mode (ThermoFisher Scientific, Bremen, Germany). NMR spectra were recorded with a Bruker Avance III HD 500 spectrometer (^1^H NMR: 500 MHz and ^13^C NMR: 125 MHz) (Bruker, Karlsruhe, Germany). Chemical shifts are given in parts per million, relative to the residual solvent peak as an internal standard, and coupling constants (*J*) are quoted in Hz. Most tetramic acids were measured in CDCl_3_ and also in CD_3_OD where they usually exist as a single (enol) tautomer. Quaternary C-atoms of tetramic acids were sometimes difficult to spot in JMOD or ^13^C NMR spectra. For these, more signals cropped up in HMBC and/or HSQC correlation spectra and were considered for peak assignment. In CDCl_3_ solution, signals of virtually all C-atoms of tetramic acids were visible yet split up in multiple, difficult to assign sets for individual tautomers both in ^1^H and JMOD/^13^C NMR spectra. In line with literature, we assume the tautomers with exocyclic C–C double bond as drawn for the 3-acyltetramic acids in Figure 1, to be the major tautomer [17]. For the purification of synthetic products, chromatography silica gel 60 (40–63 μm) or silica gel RP18 (40–63 μm) were used. Analytical thin layer chromatography (TLC) was carried out using Merck silica gel 60 F_254_ pre-coated aluminum-backed plates. Analytical HPLC was performed on a Shimadzu Nexera XR (Shimadzu GmbH, Duisburg, Germany) using a Knauer Eurospher II C18-column (150 × 4 mm) (Knauer GmbH, Berlin, Germany). Chiral HPLC was performed on a Beckmann System Gold Programmable Solvent Modul 126 using a Phenomenex Lux^®^ Amylose-1-HPLC-column (100 × 4.6 mm) (Phenomenex Ltd., Aschaffenburg, Germany). All air- and water-sensitive reactions were carried out under a dry argon atmosphere.

### 4.2. Compounds

**(*S*,*Z*)-tert-Butyl-2-(4-(allyloxy)benzyl)-4-((*E*)-1-hydroxyhepta-2,6-dien-1-ylidene)-3,5-dioxopyrrolidine-1-carboxylate (12)**. Tetramic acid **11** [4] (1.90 g, 5.50 mmol, 1.10 eq) in dry THF (305 mL) was treated with ketenylidenetriphenylphosphorane (1.66 g, 5.50 mmol, 1.10 eq) in dry THF (140 mL) over 20 min while refluxing. After stirring for 2 h, KO*t*Bu (0.62 g, 5.50 mmol, 1.10 eq) was added. The solution was stirred for a further 20 min, before 4-pentenal (0.42 g, 5.00 mmol, 1.00 eq) in dry THF (65 mL) was added over a period of 15 min. Stirring at reflux was continued for 4 h and for 17 h at room temperature. The solvent was concentrated under reduced pressure and the crude product was dissolved in CH_2_Cl_2_ (300 mL). It was washed with sat. NH_4_Cl solution (200 mL). The aqueous phase was extracted with CH_2_Cl_2_ (3 × 100 mL), the combined organic phases were washed with brine (300 mL) and dried over Na_2_SO_4_. Removal of the solvent and purification by column chromatography on reversed phase silica gel (RP18, 40% MeCN in H_2_O + 0.1% HCOOH → 60% MeCN in H_2_O + 0.1% HCOOH → 70% MeCN in H_2_O + 0.1% HCOOH → 80% MeCN in H_2_O + 0.1% HCOOH → 100% MeCN + 0.1% HCOOH) afforded 3-acyltetramic acid **12** (1.24 g, 2.74 mmol, 55%). *R_f_* = 0.88 (10% MeOH in CH_2_Cl_2_); $[\alpha {]}_{D}^{20}$ −95.0° (c 1.00, MeOH); Major tautomer: ^1^H NMR (500 MHz, CD_3_OD) *δ* 7.29 (dt, *J* = 15.6, 6.9 Hz, 1H), 7.12 (d, *J* = 15.6 Hz, 1H), 6.90 (m, 2H), 6.76 (m, 2H), 6.01 (ddt, *J* = 17.3, 10.7, 5.3 Hz, 1H), 5.83 (ddt, *J* = 17.0, 10.3, 6.7 Hz, 1H), 5.34 (dq, *J* = 17.3, 1.6 Hz, 1H), 5.20 (dq, *J* = 10.7, 1.6 Hz, 1H), 5.08 (dq, *J* = 17.0, 1.4 Hz, 1H), 5.02 (dq, *J* = 10.3, 1.6 Hz, 1H), 4.53 (m, 1H), 4.47 (dt, *J* = 5.3, 1.6 Hz, 2H), 3.35 (dd, *J* = 14.0, 5.5 Hz, 1H), 3.20 (dd, *J* = 14.0, 2.6 Hz, 1H), 2.45 (q, *J* = 6.9 Hz, 2H), 2.27 (q, *J* = 6.9 Hz, 2H), 1.62 (s, 9H) ppm; Significant signals minor tautomer: ^1^H NMR (500 MHz, CD_3_OD) *δ* 5.00 (m, 2H), 4.60 (m, 1H), 4.43 (m, 2H), 3.39 (m, 1H), 3.07 (m, 1H), 2.10 (m, 2H) ppm; ^13^C NMR (125 MHz, CD_3_OD) *δ* 159.3, 153.9, 138.2, 134.9, 131.8, 127.6, 122.1, 117.4, 116.2, 115.6, 69.7, 35.9, 35.7, 33.7, 33.0, 28.4 ppm; Major tautomer: ^1^H NMR (500 MHz, CDCl_3_) *δ* 7.30–7.11 (m, 2H), 6.92 (m, 2H), 6.75 (m, 2H), 6.01 (ddt, *J* = 17.2, 10.5, 5.3 Hz, 1H), 5.78 (ddt, *J* = 17.0, 10.3, 6.5 Hz, 1H), 5.37 (dq, *J* = 17.2, 1.6 Hz, 1H), 5.25 (dq, *J* = 10.5, 1.6 Hz, 1H), 5.11–4.91 (m, 2H), 4.46 (dt, *J* = 5.3, 1.6 Hz, 2H), 4.38 (m, 1H), 3.40–3.30 (m, 1H), 3.25 (m, 1H), 2.45 (q, *J* = 6.7 Hz, 2H), 2.27 (q, *J* = 6.7 Hz, 2H), 1.61 (s, 9H) ppm; Significant signals minor tautomer: ^1^H NMR (500 MHz, CDCl_3_) *δ* 4.60 (m, 1H), 3.38 (m, 1H), 3.20 (m, 1H), 2.40 (m, 2H), 2.24 (m, 2H) ppm; Major tautomer: ^13^C NMR (125 MHz, CDCl_3_) *δ* 192.5, 176.3, 173.8, 157.7, 152.3, 149.0, 136.8, 133.3, 130.84, 126.5, 121.7, 117.8, 116.0, 114.7, 100.7, 84.1, 68.8, 65.7, 35.1, 32.7, 32.1, 28.2 ppm; Significant signals minor tautomer: ^13^C NMR (125 MHz, CDCl_3_) *δ* 201.0, 178.4, 164.6, 157.8, 153.2, 150.2, 130.8, 126.4, 121.4, 114.8, 102.9, 83.4, 63.2, 34.9, 32.8, 32.0, 28.3 ppm; IR *ν*_max_ 2981 (w), 2937 (w), 2367 (w), 1769 (w), 1712 (m), 1642 (m), 1610 (m), 1578 (m), 1511 (m), 1414 (w), 1396 (w), 1370 (m), 1349 (m), 1304 (s), 1248 (m), 1150 (m), 1028 (w), 996 (w) cm^–1^; HRMS (ESI) *m*/*z* [M + Na]^+^ calcd. for C_26_H_31_NO_6_Na^+^ 476.20436, found 476.20380.

**(3*S*,6*Z*,8*E*,12*E*)-4-Aza-*N*-(tert-butoxycarbonyl)-7-hydroxy-15-oxa-5,21-dioxo-tricyclo-[14.2.2.1^3,6^]henicosa-1(18),6,8,12,16(17),19-hexaene (13)**. 3-Acyltetramic acid **12** (207 mg, 456 μmol, 1.00 eq) in dry, degassed CH_2_Cl_2_ (90 mL) was treated with 2nd generation Grubbs catalyst (39 mg, 46 μmol, 10 mol%). The solution was stirred at reflux for 18 h. The solvent was removed under reduced pressure and the crude product was purified by column chromatography on reversed phase silica gel (RP18, 40% MeCN in H_2_O + 0.1% HCOOH → 50% MeCN in H_2_O + 0.1% HCOOH → 60% MeCN in H_2_O + 0.1% HCOOH → 70% MeCN in H_2_O + 0.1% HCOOH → 80% MeCN in H_2_O + 0.1% HCOOH → 100% MeCN + 0.1% HCOOH) to afford **13** as pale brown resin (128 mg, 301 µmol, 66%). *R_f_* = 0.75 (5% MeOH in CH_2_Cl_2_); $[\alpha {]}_{D}^{20}$ −19.6° (c 1.00, CHCl_3_); ^1^H NMR (500 MHz, CD_3_OD) *δ* 6.90 (dt, *J* = 15.6, 7.6 Hz, 1H), 6.80 (d, *J* = 8.5 Hz, 2H), 6.74–6.61 (m, 2H), 6.58 (d, *J* = 15.6 Hz, 1H), 5.54 (dt, *J* = 15.3, 7.6 Hz, 1H), 5.41 (dt, *J* = 15.3, 5.7 Hz, 1H), 4.63–4.48 (m, 3H), 3.29 (dd, *J* = 13.8, 3.2 Hz, 1H), 3.04 (dd, *J* = 13.8, 3.2 Hz, 1H), 2.52–2.26 (m, 4H), 1.63 (s, 9H) ppm; Major tautomer: ^13^C NMR (125 MHz, CD_3_OD) *δ* 174.7 (HMBC correlation), 158.4, 153.1, 134.1, 131.1, 128.3, 126.6, 122.9, 118.0, 117.6, 115.3, 84.9, 67.7, 66.0, 37.0, 33.6, 31.9, 28.4 ppm; Significant signals minor tautomer: ^13^C NMR (125 MHz, CD_3_OD) *δ* 159.9, 132.3, 117.2, 114.9 ppm; Major tautomer: ^1^H NMR (500 MHz, CDCl_3_) *δ* 6.95–6.48 (m, 6H), 5.50 (m, 1H), 5.35 (m, 1H), 4.62–4.39 (m, 3H), 3.27 (dd, *J* = 13.6, 3.6 Hz, 1H), 3.07 (m, 1H), 2.57–2.41 (m, 2H), 2.33–2.12 (m, 2H), 1.63 (s, 9H) ppm; Significant signals minor tautomer: ^1^H NMR (500 MHz, CDCl_3_) *δ* 3.38 (dd, *J* = 13.6, 3.6 Hz, 1H) ppm; C2, C4 and C3 not observed; IR *ν*_max_ 2973 (w), 2931 (w), 2934 (m), 1764 (m), 1713 (m), 1644 (s), 1608 (w), 1579 (s), 1508 (m), 1394 (w), 1369 (m), 1350 (s), 1306 (s), 1274 (m), 1254 (m), 1222 (m), 1159 (s), 1141 (m), 1109 (w), 978 cm^–1^; HRMS (ESI) *m*/*z* [M + Na]^+^ calcd. for C_24_H_27_NO_6_Na^+^ 448.17306, found 448.17270. 

**(3*S*,6*Z*,8*E*,12*E*)-4-Aza-7-hydroxy-15-oxa-5,21-dioxo-tri-cyclo [14.2.2.1^3,6^]henicosa-1(18),6,8,12,16(17),19-hexaene (5)**. Tetramic acid **13** (245 mg, 576 μmol, 1.00 eq) in dry CH_2_Cl_2_ (11 mL) was treated with TFA (1.10 mL) and stirred for 15 min at room temperature. Toluene (75 mL) was added and the solvent was concentrated under reduced pressure. This was repeated once to yield **5** as a pale brown foam (187 mg, 576 µmol, quant.). *R_f_* = 0.63 (10% MeOH in CH_2_Cl_2_ + 0.1% HCOOH); $[\alpha {]}_{D}^{20}\;$+92.9° (c 1.00, MeOH); ^1^H NMR (500 MHz, CD_3_OD): Diastereotopic H-atoms indicated as a, b: *δ* 7.03–6.41 (m, 6H, OC*H*C=C*H*CH_2_, CH_Ar_), 5.57 (m, 1H, OCH_2_HC=C*H*), 5.35 (m, 1H, OCH_2_*H*C=CH), 4.60/4.48 (m, 2H, ArOCH_2_), 4.05 (brs, 1H, CHN), 3.01^a^ (dd, *J* = 13.6, 3.9 Hz, 1H), 2.85^b^ (dd, *J* = 13.6, 2.0 Hz, 1H, ArCH), 2.53–2.13 (m, 4H, OCH_2_HC=CH(C*H*_2_)_2_) ppm; ^13^C NMR (125 MHz, CD_3_OD) *δ* 172.8 (HNCO), 157.9 (OC_q,Ar_), 149.8 (OCHC=*C*HCH_2_), 132.8 (OCH_2_H*C*=CH), 131.2 (CH_2_C*C*H_Ar_), 128.0 (OCH_2_HC=*C*H), 126.5 (CH_2_*C*_q,Ar_), 123.2 (OCH*C*=CHCH_2_), 117.6 (OC*C*H_Ar_), 67.4 (ArOCH_2_), 38.0 (ArCH_2_), 33.5 (OCHC=CH*C*H_2_), 32.5 (OCH_2_HC=CH*C*H_2_) ppm; Major tautomer: ^1^H NMR (500 MHz, CDCl_3_): *δ* 7.07–6.42 (m, 6H), 5.57–5.31 (m, 2H), 4.55 (m, 2H), 4.10 (m, 1H), 3.13 (dd, *J* = 13.8, 4.1 Hz, 1H), 2.84 (m, 1H), 2.55–2.11 (m, 4H) ppm; Significant signals minor tautomer: ^1^H NMR (500 MHz, CDCl_3_) *δ* 4.20 (m, 1H), 2.90 (m, 1H) ppm; Major tautomer: ^13^C NMR (125 MHz, CDCl_3_) *δ* 195.6, 176.1, 171.8, 156.5, 148.6, 133.5, 132.2, 130.0, 126.8, 125.0, 122.3, 117.0, 111.6, 100.9, 66.5, 62.2, 37.6, 32.6, 31.7 ppm; Significant signals minor tautomer: ^13^C NMR (125 MHz, CDCl_3_) *δ* 203.7, 172.7, 170.2, 157.1, 149.7, 131.6, 130.8, 127.1, 125.5, 122.0, 115.7, 115.4, 104.0, 67.5, 60.2, 38.1, 32.1, 30.4 ppm; IR *ν*_max_ 3303 (m), 2927 (w), 2934 (m), 2070 (w), 1643 (s), 1576 (s), 1507 (m), 1428 (m), 1369 (w), 1338 (w), 1254 (m), 1219 (m), 1177 (m), 1115 (m), 975 (s) cm^–1^; HRMS (ESI) *m*/*z* [M + H^+^] calcd. for C_19_H_20_NO_4_^+^ 326.13868, found 326.13785.

**(3*S*,6*Z*,12*E*)-4-Aza*-N*-(tert-butoxycarbonyl)-7-hydroxy-15-oxa-5,21-dioxo-tricyclo-[14.2.2.1^3,6^]henicosa-1(18),6,12, 16(17),19-pentaene (16).** Tetramic acid **13** (77.0 mg, 181 μmol, 1.00 eq) and Wilkinson’s catalyst (17 mg, 18 μmol, 10 mol%) in dry CH_2_Cl_2_ (2.5 mL) were treated with Et_3_SiH (144 μL, 905 μmol, 5.00 eq). The solution was stirred for 19 h under reflux and the solvent was removed under reduced pressure. The crude product was dissolved in dry MeOH (2.6 mL) and KF (26.3 mg, 453 μmol, 2.50 eq) was added. After stirring for 20 h at −15 °C, more KF (26.3 mg, 453 μmol, 2.50 eq) was added and stirring was continued for a further 7 h at −15 °C. Chilled H_2_O (50 mL) and chilled brine (20 mL) were added. The aqueous phase was extracted with EtOAc (3 × 50 mL), and the combined organic phases were washed with 0.5M H_2_SO_4_ (40 mL) and dried over Na_2_SO_4_. Removal of the solvent and purification by column chromatography on reversed phase silica gel (RP18, 30% MeCN in H_2_O + 0.1% HCOOH → 40% MeCN in H_2_O + 0.1% HCOOH → 50% MeCN in H_2_O + 0.1% HCOOH → 60% MeCN in H_2_O + 0.1% HCOOH) gave **16** as a colourless foam (37 mg, 86.6 µmol, 48%). *R_f_* = 0.83 (10% MeOH in CH_2_Cl_2_); $[\alpha {]}_{D}^{20}$ +12.4° (c 1.00, MeOH); Major tautomer: ^1^H NMR (500 MHz, CD_3_OD) *δ* 6.90–6.60 (m, 4H), 5.53 (dt, *J* = 15.4, 7.9 Hz, 1H), 5.40 (dt, *J* = 15.4, 5.3 Hz, 1H), 4.64–4.49 (m, 3H), 3.41 (dd, *J* = 14.1, 4.5 Hz, 1H), 3.10 (dd, *J* = 14.1, 2.9 Hz, 1H), 2.36 (m, 2H), 2.09–1.90 (m, 2H), 1.64 (s, 9H), 1.30 (m, 2H), 1.08 (m, 2H) ppm; Significant signals minor tautomer: ^1^H NMR (500 MHz, CDCl_3_) *δ* 3.29 (dd, *J* = 14.1, 4.5 Hz, 1H), 3.03 (dd, *J* = 14.1, 2.9 Hz, 1H), 2.91 (m, 2H) ppm; Major tautomer: ^13^C NMR (125 MHz, CD_3_OD) *δ* 158.0, 150.8, 135.8, 131.7, 127.1, 127.0, 117.9, 84.7, 68.0, 35.7, 33.6, 33.2, 29.6, 28.4, 28.0 ppm; Significant signals minor tautomer: ^13^C NMR (125 MHz, CD_3_OD) *δ* 158.3, 128.2, 118.5, 67.7, 37.0, 33.4, 29.8, 28.3, 27.7 ppm; ^1^H NMR (500 MHz, CDCl_3_) *δ* 7.02–6.42 (m, 4H), 5.56–5.26 (m, 2H), 4.69–4.37 (m, 3H), 3.49–3.34 (dd, *J* = 14.0, 4.9 Hz, 1H), 3.29 (m, 1H), 3.15–3.03 (m, 1H), 2.48–1.86 (m, 3H), 1.63 (s, 9H), 1.47–0.99 (m, 4H) ppm; Major tautomer: ^13^C NMR (125 MHz, CDCl_3_) *δ* 197.4, 196.8, 164.3, 156.6, 149.8, 134.5, 131.9, 125.8, 125.70, 118.4, 102.2, 83.5, 67.6, 62.1, 35.0, 32.7, 32.22, 28.7, 28.3, 26.9 ppm; Significant signals minor tautomer: ^13^C NMR (125 MHz, CD_3_OD) *δ* 192.0, 191.8, 156.1, 136.6, 130.0, 125.75, 114.0, 105.5, 84.4, 67.0, 65.8, 34.7, 34.5, 32.24, 28.4, 27.6, 26.7, 26.0 ppm; IR *ν*_max_ 2978 (w), 2935 (w), 2863 (w), 1778 (m), 1744 (m), 1712 (s), 1662 (m), 1607 (s), 1509 (s), 1475 (w), 1456 (w), 1440 (m), 1423 (w), 1395 (w), 1366 (m), 1350 (s), 1305 (s), 1272 (m), 1258 (s), 1217 (s), 1150 (s), 1111 (m), 1081 (w), 1017 (w), 971 (m) cm^–1^; HRMS (ESI) *m*/*z* [M + Na^+^] calcd. for C_24_H_29_NO_6_Na^+^ 450.18871, found 450.18776.

**(*S*)-tert-Butyl-2-(4-(allyloxy)benzyl)-3-(hept-6-enoyloxy)-5-oxo-2,5-dihydro-1H-pyrrole-1-carboxylate (14).** 6-Heptenoic acid (2.11 mL, 15.6 mmol, 1.00 eq) in dry CH_2_Cl_2_ (78 mL) was treated with EDC∙HCl (3.59 g, 18.7 mmol, 1.20 eq) and DMAP (0.38 g, 3.12 mmol, 0.20 eq) at 0 °C. The solution was stirred for 20 min, before tetramic acid **11** (5.93 g, 17.2 mmol, 1.1 eq) was added at room temperature. After stirring for 4 h, the reaction was quenched with 0.5M H_2_SO_4_ (250 mL). The organic phase was separated and the aqueous phase was extracted with EtOAc (3 × 150 mL). The combined organic phases were washed with brine (200 mL) and dried over Na_2_SO_4_. After removal of the solvent under reduced pressure, the crude product was purified by column chromatography (silica gel 60, 10% EtOAc in hexanes → 15% EtOAc in hexanes → 20% EtOAc in hexanes → 25% EtOAc in hexanes) to obtain **14** as an orange resin (5.64 g, 12.4 mmol, 79%). *R_f_* = 0.48 (30% EtOAc in hexanes); $[\alpha {]}_{D}^{20}$ +107.5° (c 1.00, MeOH); ^1^H NMR (500 MHz, CDCl_3_) *δ* 6.93–6.83 (m, 2H), 6.81–6.71 (m, 2H), 6.03 (m, 1H), 5.88 (s, 1H), 5.79 (ddt, *J* = 17.0, 10.3, 6.7 Hz, 1H), 5.40 (m, 1H), 5.28 (m, 1H), 5.06–4.94 (m, 2H), 4.77 (dd, *J* = 6.0, 2.8 Hz, 1H), 4.48 (m, 2H), 3.29 (dd, *J* = 14.3, 6.2 Hz, 1H), 3.14 (dd, *J* = 14.3, 2.8 Hz, 1H), 2.48 (td, *J* = 7.4, 1.9 Hz, 2H), 2.09 (m, 2H), 1.74–1.65 (m, 2H), 1.60 (s, 9H), 1.46 (qn, *J* = 7.7 Hz, 2H) ppm; ^13^C NMR (125 MHz, CDCl_3_) *δ* 168.8, 168.2, 165.3, 157.9, 149.5, 138.0, 133.3, 130.5, 126.2, 117.9, 115.3, 114.8, 108.3, 83.3, 68.9, 60.7, 35.0, 34.3, 33.4, 28.4, 28.2, 23.9 ppm; IR *ν*_max_ 3075 (w), 2975 (w), 2939 (w), 2863 (w), 1777 (s), 1744 (s), 1712 (s), 1633 (m), 1611 (w), 1582 (w), 1514 (s), 1478 (w), 1457 (w), 1424 (w), 1392 (w), 1370 (m), 1356 (m), 1320 (s), 1248 (s), 1226 (m), 1172 (s), 1158 (s), 1115 (m), 1064 (s), 1032 (m), 996 (m) cm^–1^; HRMS (ESI) *m*/*z* [M + Na^+^] calcd. for C_26_H_33_O_6_NNa^+^ 478.22001, found 478.21968. 

**(*S,Z*)-tert-Butyl-2-(4-(allyloxy)benzyl)-4-(1-hydroxyhept-6-en-1-ylidene)-3,5-dioxopyrrolidine-1-carboxylate (15)**. 4-*O*-acyltetramic acid **14** (5.54 g, 12.2 mmol, 1.0 eq) in dry CH_2_Cl_2_ (122 mL) was treated with dry NEt_3_ (2.04 mL, 14.6 mmol, 1.2 eq) at room temperature and stirred for 10 min. DMAP (743 mg, 6.1 mmol, 0.5 eq) was added and the solution was stirred for a further 24 h. NaHCO_3_ (200 mL) was added and the aqueous phase was extracted with EtOAc (2 × 150 mL). The combined organic phases were washed with brine (200 mL) and dried over Na_2_SO_4_. Removal of the solvent under reduced pressure and purification by column chromatography on reversed phase silica gel (RP18, 40% MeCN in H_2_O + 0.1% HCOOH → 60% MeCN in H_2_O + 0.1% HCOOH → 80% MeCN in H_2_O + 0.1% HCOOH → 100% MeCN + 0.1% HCOOH) afforded **15** as an orange resin (4.54 g, 9.97 mmol, 82%). *R_f_* = 0.91 (10% MeOH in CH_2_Cl_2_); $[\alpha {]}_{D}^{20}$ −31.8° (c 1.00, MeOH); ^1^H NMR (500 MHz, CD_3_OD) *δ* 6.90 (m, 2H), 6.77 (m, 2H), 6.01 (m, 1H), 5.78 (ddt, *J* = 17.1, 10.5, 7.2 Hz, 1H), 5.35 (dd, *J* = 17.1, 1.5 Hz, 1H), 5.21 (m, 1H), 5.01 (m, 1H), 4.94 (m, 1H), 4.58 (s, 1H), 4.46 (d, *J* = 5.2 Hz, 2H), 3.38 (dd, *J* = 14.2, 5.4 Hz, 1H), 3.18 (dd, *J* = 14.2, 2.3 Hz, 1H), 2.75 (t, *J* = 6.4 Hz, 2H), 2.04 (q, *J* = 7.2 Hz, 2H), 1.62 (s, 9H), 1.57–1.45 (m, 2H), 1.34 (m, 2H) ppm; ^13^C NMR (125 MHz, CD_3_OD) *δ* 195.2 (HMBC correlation), 159.3, 139.5, 134.9, 131.9, 127.4, 117.4, 115.5, 115.3, 84.8, 69.7, 64.6 (HMBC correlation), 35.8, 34.4, 29.4, 28.4, 26.2 ppm; Major tautomer: ^1^H NMR (500 MHz, CDCl_3_) *δ* 6.92 (m, 2H), 6.75 (m, 2H), 6.02 (m, 1H), 5.85–5.70 (m, 1H), 5.38 (m, 1H), 5.27 (m, 1H), 5.07–4.91 (m, 2H), 4.39 (dd, *J* = 5.4, 2.7 Hz, 1H), 4.46 (d, *J* = 5.3 Hz, 2H), 3.33 (dd, *J* = 14.1, 5.8 Hz, 1H), 3.24 (dd, *J* = 14.1, 2.6 Hz, 1H), 2.93–2.61 (m, 2H), 2.07 (q, *J* = 7.1 Hz, 2H), 1.61 (s, 9H), 1.60–1.29 (m, 4H) ppm; Significant signals minor tautomer: ^1^H NMR (500 MHz, CDCl_3_) *δ* 7.10 (m, 2H), 6.87 (m, 2H), 4.64 (dd, *J* = 5.4, 2.7 Hz, 1H), 4.52 (d, *J* = 5.3 Hz, 2H), 3.41 (dd, *J* = 14.1, 5.8 Hz, 1H), 3.21 (dd, *J* = 14.1, 2.6 Hz, 1H), 2.01 (q, *J* = 7.1 Hz, 2H) ppm; Major tautomer: ^13^C NMR (125 MHz, CDCl_3_) *δ* 196.5, 192.4, 164.4, 157.8, 149.1, 138.3, 133.32, 130.9, 126.4, 117.8, 115.1, 114.7, 102.5, 84.2, 68.82, 65.8, 35.0, 33.4, 32.9, 28.3, 28.2, 25.5 ppm; Significant signals minor tautomer: ^13^C NMR (125 MHz, CDCl_3_) *δ* 157.9, 150.0, 138.4, 133.30, 130.8, 126.0, 117.9, 115.3, 114.9, 105.6, 83.5, 68.80, 61.9, 34.9, 34.8, 32.7, 28.5, 28.4, 24.7 ppm; IR *ν*_max_ 3075 (w), 2978 (w), 2932 (w), 2860 (w), 1770 (w), 1744 (m), 1716 (s), 1667 (m), 1640 (m), 1604 (s), 1510 (s), 1457 (w), 1421 (m), 1395 (w), 1366 (m), 1349 (s), 1301 (s), 1241 (s), 1223 (s), 1172 (s), 1151 (s), 1025 (m), 996 (m), 967 (m), 913 (s) cm^–1^; HRMS (ESI) *m*/*z* [M + Na^+^] calcd. for C_26_H_33_O_6_NNa^+^ 478.22001, found 478.21944. 

**(3*S*,6*Z*,12*E*)-4-Aza-*N*-(tert-butoxycarbonyl)-7-hydroxy-15-oxa-5,21-dioxo-tricyclo[14.2.2.1^3,6^]henicosa-1(18),6,12,16(17),19-pentaene (16) from 15**. 3-Acyltetramic acid **15** (2.30 g, 5.04 mmol, 1.00 eq) in dry, degassed CH_2_Cl_2_ (1.00 L) was treated with 2nd generation Grubbs catalyst (428 mg, 504 μmol, 10 mol%). The solution was stirred at reflux for 24 h. The solvent was removed under reduced pressure and the crude product was purified by column chromatography on reversed phase silica gel (RP18, 40% MeCN in H_2_O + 0.1% HCOOH → 60% MeCN in H_2_O + 0.1% HCOOH → 80% MeCN in H_2_O + 0.1% HCOOH → 100% MeCN + 0.1% HCOOH) to yield **16** as a pale brown foam (1.78 g, 4.16 mmol, 83%). For analytical data see above.

**(3*S*,6*Z*,12*E*)-4-Aza-7-hydroxy-15-oxa-5,21-dioxo-tricyclo[14.2.2.1^3,6^]henicosa- 1(18),6,12,16(17),19-pentaene (4)**. Carbamate **16** (926 mg, 2.17 mmol, 1.00 eq) in dry CH_2_Cl_2_ (43 mL) was treated with TFA (4.3 mL) and stirred for 15 min at room temperature. Toluene (100 mL) was added and the solvent was concentrated under reduced pressure. This was repeated once to yield **4** as a pale brown foam (709 mg, 2.17 mmol, quant.). *R_f_* = 0.64 (10% MeOH in CH_2_Cl_2_ + 0.01% HCOOH); $[\alpha {]}_{D}^{20}$ +52.2° (c 1.00, MeOH); ^1^H NMR (500 MHz, CD_3_OD): Diastereotopic H-atoms indicated as a, b: *δ* 7.09–6.68 (m, 4H, CH_Ar_), 5.58 (dt, *J* = 15.1, 7.8 Hz, 1H, OCH_2_*H*C=CH), 5.33 (m, 1H, OCH_2_HC=C*H*), 4.64^a^ (dd, *J* = 13.9, 7.7 Hz, 1H, ArOCH), 4.53^b^ (m, 1H, ArOCH), 4.15 (t, *J* = 3.3 Hz, 1H, CHN), 3.11–2.67^a^ (brs, 1H, OCCH), 3.07^a^ (dd, *J* = 14.1, 3.8 Hz, 1H, ArCH), 2.91^b^ (dd, *J* = 14.1, 3.5 Hz, 1H, ArCH), 2.42–1.86^b^ (brs, 1H, OCCH), 2.05^a^ (m, 1H, HC=CHC*H*), 1.94^b^ (m, 1H, HC=CHC*H*), 1.34–1.05 (m, 4H, HC=CHCH_2_(C*H*_2_)_2_) ppm; ^13^C NMR (125 MHz, CD_3_OD) *δ* 157.5 (OC_q,Ar_), 127.4 (OCH_2_*C*H=CH), 126.9 8 (CH_2_*C*_q,Ar_), 118.3 (OC*C*H_Ar_), 67.8 (ArOCH_2_), 36.6 (ArCH_2_), 33.6 (OC*C*H_2_), 33.4 (HC=CH*C*H_2_), 29.8 (HC=CHCH_2_(*C*H_2_)_2_), 28.5 (HC=CHCH_2_(*C*H_2_)_2_) ppm; Major tautomer: ^1^H NMR (500 MHz, CDCl_3_) *δ* 7.10–6.58 (m, 4H), 5.54 (m, 1H), 5.39 (m, 1H), 4.60 (m, 2H), 4.16 (m, 1H), 3.28–3.16 (m, 2H), 2.87 (dd, *J* = 14.1, 1.7 Hz, 1H), 2.12–1.84 (m, 3H), 1.40–1.04 (m, 4H) ppm; Significant signals minor tautomer: ^1^H NMR (500 MHz, CDCl_3_) *δ* 5.49 (m, 1H), 5.32 (m, 1H), 4.29 (s, 1H), 2.87 (m, 1H) ppm; Major tautomer: ^13^C NMR (125 MHz, CDCl_3_) *δ* 194.5, 188.6, 175.8, 156.0, 136.8, 132.0, 130.3, 125.7, 125.6, 117.9, 114.1, 101.5, 67.0, 62.4, 36.2, 32.43, 32.41, 28.7, 27.6 ppm; Significant signals minor tautomer: ^13^C NMR (125 MHz, CDCl_3_) *δ* 201.2 (HMBC correlation), 192.4, 170.3, 156.5, 134.9, 131.2, 118.2, 104.7, 67.6, 59.8, 36.4, 33.1, 32.1, 28.5, 27.1 ppm; IR *ν*_max_ 3255 (w), 2929 (w), 2857 (w), 1770 (w), 1646 (s), 1608 (s), 1508 (s), 1433 (m), 1367 (w), 1305 (w), 1259 (m), 1214 (s), 1176 (s), 1159 (s), 1113 (m), 1075 (m), 1062 (m), 1015 (m), 973 (s) cm^–1^. HRMS (ESI) *m*/*z* [M + H^+^] calcd. for C_19_H_22_NO_4_^+^ 328.15433, found 328.15343.

**(3*S*,6*Z*,12*S*,13*S*)-4-Aza-7,12,13-trihydroxy-15-oxa-5,21-dioxo-tricyclo[14.2.2.1^3,6^]henicosa-1(18),6,16(17),19-tetraene (7)**. AD-mix α (2.86 g, 1.4 g/mmol) in H_2_O (10.3 mL) was treated with alkene **16** (874 mg, 2.04 mmol, 1.00 eq) in *t*BuOH (10.3 mL) at 0 °C. The two-phase mixture was stirred at 7 °C for 5 d, before more AD-mix α (1.43 g, 0.7 g/mmol) was added. After stirring for a further 4 d, the mixture was treated with Na_2_SO_3_ (3.60 g, 28.6 mmol, 14.0 eq) and stirred for 2 h at room temperature. H_2_O was added to dissolve the precipitate. The aqueous phase was washed with EtOAc (10 mL) and the organic phase was extracted with H_2_O (10 mL). The solvent was removed under reduced pressure and the crude product was suspended in MeOH. The precipitate was filtered off and washed with MeOH. Concentration of the filtrate and purification of the residue by column chromatography on reversed phase silica gel (RP18, H_2_O + 0.1% HCOOH → 20% MeCN in H_2_O + 0.1% HCOOH → 40% MeCN in H_2_O + 0.1% HCOOH → 60% MeCN in H_2_O + 0.1% HCOOH) gave a mixture of Boc-protected and deprotected tetramic acid **7**. This was dissolved in dry CH_2_Cl_2_ (25 mL) and treated with TFA (1.5 mL). After stirring for 15 min at room temperature, toluene (100 mL) was added. The solvent was concentrated under reduced pressure and more toluene (100 mL) was added. Removal of the solvent under reduced pressure gave diol **7** as a yellowish foam and as a mixture of two diastereomers according to HPLC and NMR spectra. Yield: 323 mg (894 µmol, 44%, *dr* 2.3:1). *R_f_* = 0.63 (10% MeOH in CH_2_Cl_2_ + 0.1% HCOOH); ^1^H NMR (500 MHz, CD_3_OD): Signals of major diastereomer marked as A, signals of minor diastereomer marked as B; diastereotopic H-atoms indicated as a, b: *δ* 7.13–6.75 (m, 4H, CH_Ar_), 4.26 (m, 1H, ArOCH^a^), 4.22 (m, 1H, CHN), 4.07 (dd, *J* = 11.7, 8.8 Hz, 1H, ArOCH^b,B^), 4.05 (m, 1H, *J* = 11.8, 8.9 Hz, 1H, ArOCH^b,A^), 3.80 (m, 1H, ArOCH_2_C*H*OH^A^), 3.60 (m, 2H, ArOCH_2_C*H*OH^B^, ArOCH_2_CHOHC*H*OH^B^), 3.46 (dt, *J* = 9.6, 2.7, 3.9 Hz, 1H, ArOCH_2_CHOHC*H*OH^A^), 3.10 (dt, *J* = 14.0, 3.8 Hz, 1H, ArCH^a^), 2.97 (dt, *J* = 14.0, 3.2 Hz, 1H, ArCH^b^), 1.67–1.17 (m, 4H, (CH_2_)_2_), 0.88–0.58 (m, 2H, OCCH_2_C*H*_2_) ppm; OCCH_2_ not observed; Major diastereomer: ^13^C NMR (125 MHz, CD_3_OD): *δ* 189.0 (CHO, HMBC correlation), 157.8 (OC_q,Ar_), 131.1 (CH_2_C*C*H_Ar_, HMBC correlation), 127.2 (CH_2_*C*_q,Ar_, HMBC correlation), 118.4 (OC*C*H_Ar_), 113.7 (OC*C*H_Ar_), 69.9 (ArOCH_2_CHOH*C*HOH), 67.5 (ArOCH_2_), 67.4 (ArOCH_2_*C*HOH, HSQC correlation), 36.6 (ArCH_2_), 34.5 (ArOCH_2_(HCOH)_2_*C*H_2_), 32.9 (OC*C*H_2_), 27.3 (ArOCH_2_(HCOH)_2_CH_2_*C*H_2_), 25.4 (OCCH_2_*C*H_2_) ppm; Minor diastereomer ^13^C NMR (125 MHz, CD_3_OD): *δ* 131.9 (HMBC correlation), 126.5 (HMBC correlation), 119.4, 115.2, 69.7, 68.5, 32.8, 25.9 ppm; IR *ν*_max_ 3354 (m), 2933 (m), 1646 (s), 1607 (s), 1508 (s), 1433 (w), 1338 (w), 1255 (m), 1216 (m), 1177 (w), 1113 (w), 1016 (w) cm^–1^; HRMS (ESI) *m*/*z* [M + H^+^] calcd. for C_19_H_24_NO_4_^+^ 362.15981, found 362.15894.

**(3*S*,6*Z*)-4-Aza-12-bromo-7,13-dihydroxy-15-oxa-5,21-dioxo-tricyclo[14.2.2.1^3,6^]henicosa-1(18),6,16(17),19-tetraene (8)**. Alkene **16** (660 mg, 1.54 mmol, 1.00 eq) in DMSO (8 mL) was treated with H_2_O (41.7 μL, 2.32 mmol, 1.50 eq) and NBS (412 mg, 2.23 mmol, 1.50 eq) at 8 °C. After stirring the solution for 22 h at room temperature, sat. NaHCO_3_ solution (50 mL) was added. The aqueous phase was extracted with EtOAc (3 × 50 mL) and the combined organic phases were dried over Na_2_SO_4_. The solvent was removed under reduced pressure and the crude mixture of products was purified by column chromatography on reversed phase silica gel (RP18, 20% MeCN in H_2_O + 0.1% HCOOH → 30% MeCN in H_2_O + 0.1% HCOOH → 40% MeCN in H_2_O + 0.1% HCOOH → 50% MeCN in H_2_O + 0.1% HCOOH → 60% MeCN in H_2_O + 0.1% HCOOH → 80% MeCN in H_2_O + 0.1% HCOOH). Bromohydrins **8** and a side product with an additional bromo substituent were obtained separately and only partially deprotected. The mixture of bromohydrin **8** and its *N*-Boc-protected derivative was dissolved in dry CH_2_Cl_2_ (9 mL) and treated with TFA (900 μL). The solution was stirred for 15 min at room temperature and toluene (100 mL) was added. The mixture was concentrated under reduced pressure and toluene (50 mL) was added again. Removal of the solvent under reduced pressure gave **8** as a yellowish foam and as a mixture of two inseparable diastereomers of initially unknown *dr* according to ^13^C and ^1^H NMR spectra. Yield (**8**): 196 mg (462 µmol, 30%). *R_f_* = 0.41 (10% MeOH in CH_2_Cl_2_); ^1^H NMR (500 MHz, CD_3_OD): Signals of major diastereomer marked as A, signals of minor diastereomer marked as B; diastereotopic H-atoms indicated as a, b: *δ* 7.24–6.75 (m, 4H, CH_Ar_), 4.67–4.19 (m, 4H, CHN, ArOCH_2_, CHBr), 3.73 (m, 1H, C*H*OH), 3.14 (m, 2H, ArCH^a^, OCCH^a,A^), 2.96 (dd, *J* = 14.0, 3.9 Hz, 1H, ArCH^b^), 1.60–0.91 (m, 5H, CBrC*H*_2_C*H^a^*C*H*_2_), 0.76–0.26 (m, 1H, CBrC*H*_2_C*H^b^*C*H*_2_) ppm; ^1^H NMR (500 MHz, CDCl_3_) *δ* 7.20–6.72 (m, 4H), 4.60–4.11 (m, 4H), 3.78 (m, 1H), 3.60–3.20 (m, 2H), 2.94 (m, 1H), 2.20–2.00 (m, 1H), 1.63–1.33 (m, 3H), 1.30–0.96 (m, 2H), 0.66–0.30 (m, 1H) ppm; Major tautomer: ^13^C NMR (125 MHz, CDCl_3_): Signals of major diastereomer marked as A, signals of minor diastereomer marked as B: *δ* 194.3^B^ (CO), 194.2^A^ (CO), 188.2 (COH), 175.6^B^ (HNCO),175.5^A^ (HNCO), 157.1 (OC_q,Ar_), 131.4^B^ (CH_2_C*C*H_Ar_), 130.1^A^ (CH_2_C*C*H_Ar_), 127.6 (CH_2_*C*_q,Ar_), 117.0 (OC*C*H_Ar_), 101.90^B^ (NCO*C*CO), 101.88^A^ (NCO*C*CO), 73.0 (CHOH), 69.6 (ArOCH_2_), 62.0^B^ (HCNH), 61.9^A^ (HCNH), 55.9 (CHBr), 36.2 (ArCH_2_), 34.2 (CHBr*C*H_2_), 33.1 (OC*C*H_2_), 27.4 (CHBr*C*H_2_), 26.0 (OCCH_2_*C*H_2_), 24.6 (CHBrCH_2_*C*H_2_) ppm; Significant signals minor tautomer: ^13^C NMR (125 MHz, CDCl_3_): Signals of major diastereomer marked as A, signals of minor diastereomer marked as B: *δ* 202.6^B^, 202.5^A^, 189.03^B^, 189.00^A^, 169.1^B^, 169.0^A^, 156.3, 133.3^A^, 132.4^B^, 127.5, 116.2^A^, 115.3^B^, 106.0, 67.7, 59.6^B^, 59.5^A^, 53.7, 36.6, 33.4, 31.4, 24.6 ppm; IR *ν*_max_ 3356 (m), 2936 (m), 1652 (s), 1609 (s), 1508 (s), 1462 (w), 1374 (w), 1254 (m), 1217 (m), 1177 (w), 1114 (w), 1043 (w) cm^–1^; HRMS (ESI) *m*/*z* [M + H^+^] calcd. for C_19_H_23_NO_5_Br^+^ 424.07451, found 424.07521.

**(3*S*,6*E*)-4-Aza-13,16-dioxa-5,22-dioxo-tetracyclo[15.2.2.1^3,6^.0^12,14^]docosa-1(19),6,17(18),20-tetraene (6)**. Bromohydrin **8** (248 mg, 473 μmol, 1.00 eq) in dry THF (1.5 mL) was treated with KO*t*Bu (86.7 mg, 709 μmol, 1.50 eq) at 0 °C. The suspension was stirred for 4 d at room temperature and then more KO*t*Bu (58 mg, 473 μmol, 1.00 eq) was added. Stirring was continued for 1 d and H_2_O (5 mL) as well as EtOAc (5 mL) were added. The organic phase was separated and extracted with H_2_O (5 mL). The combined aqueous phases were concentrated under reduced pressure. The crude product was purified by column chromatography on reversed phase silica gel (RP18, 20% MeCN in H_2_O + 0.1% HCOOH → 30% MeCN in H_2_O + 0.1% HCOOH → 40% MeCN in H_2_O + 0.1% HCOOH → 50% MeCN in H_2_O + 0.1% HCOOH → 80% MeCN in H_2_O + 0.1% HCOOH) to yield a virtually pure product. Another column chromatography on reversed phase silica gel (RP18, 0% MeCN in H_2_O + 0.1% HCOOH → 20% MeCN in H_2_O + 0.1% HCOOH → 30% MeCN in H_2_O + 0.1% HCOOH → 40% MeCN in H_2_O + 0.1% HCOOH → 50% MeCN in H_2_O + 0.1% HCOOH→ 80% MeCN in H_2_O + 0.1% HCOOH) afforded epoxide **6** as a pale brown foam and as a 9:1 mixture of two diastereomers according to ^13^C and ^1^H NMR spectra. Yield: 76.0 mg (221 µmol, 47%). *R_f_* = 0.28 (10% MeOH in CH_2_Cl_2_); ^1^H NMR (500 MHz, CD_3_OD): Signals of major diastereomer marked as A, signals of minor diastereomer marked as B¸ diastereotopic H-atoms indicated as a, b: *δ* 7.05 (m, 2H, OC(CH)_2_), 6.77 (m, 2H, CH_2_C(CH)_2_), 4.55^A,a^ (dd, *J* = 14.0, 3.1 Hz, 1H, ArOCH), 4.40^B,a^ (m, 1H, ArOCH), 4.21 (t, *J* = 3.2 Hz, 1H, CHN), 4.03^A,b^ (dd, *J* = 14.0, 3.1 Hz, 1H, ArOCH), 3.95^B,b^ (m, 1H, ArOCH), 3.14^a^ (dd, *J* = 14.1, 3.5 Hz, 1H, ArCH), 3.22–2.69^a^ (brs, 1H, OCCH), 3.05 (m, 1H, ArOCH_2_C*H*O), 2.93^b^ (dd, *J* = 14.1, 3.5 Hz, 1H, ArCH), 2.65 (m, 1H, ArOCH_2_CHOC*H*), 1.71^a^ (m, 1H, ArOCH_2_CHOCHC*H*), 1.63–1.15 (m, 3H, OCCH^b^, ArOCH_2_CHOCHCH_2_C*H*_2_), 1.15–0.41 (m, 3H, ArOCH_2_CHOCHCH^b^, OCCH_2_C*H*_2_) ppm; ^13^C NMR (125 MHz, CD_3_OD): Signals of major diastereomer marked as A, signals of minor diastereomer marked as B: *δ* 187.5 (HMBC correlation), 157.7 (OC_q,Ar_), 132.7^A^(CH_2_C*C*H_Ar_), 132.5^B^ (CH_2_C*C*H_Ar_), 128.2 (CH_2_*C*_q,Ar_), 116.5^B^ (OC*C*H_Ar_), 116.0^A^ (OC*C*H_Ar_), 113.2^A^ (OC*C*H_Ar_), 66.6^B^ (ArOCH_2_), 66.4^A^ (ArOCH_2_), 62.4 (HCNH, HSQC correlation), 58.5 (OCH_2_*C*HOCH), 58.4 (OCH_2_CHO*C*H), 36.3 (CH_2_Ar), 33.3 (ArOCH_2_CHOCH*C*H_2_), 31.9 (OC*C*H_2_), 27.3 (ArOCH_2_CHOCHCH_2_*C*H_2_), 24.8^A^ (OCCH_2_*C*H_2_), 24.7^B^ (OCCH_2_*C*H_2_) ppm; ^1^H NMR (500 MHz, CDCl_3_) *δ* 7.25–6.43 (m, 5H), 4.53 (t, *J* = 11.7 Hz, 1H), 4.44–3.83 (m, 2H), 3.40 (m, 1H), 3.24 (m, 1H), 3.12–2.80 (m, 2H), 2.71 (m, 1H), 2.21 (m, 1H), 1.92–1.42 (m, 3H), 1.19–0.35 (m, 3H) ppm; Major tautomer: ^13^C NMR (125 MHz, CDCl_3_): Signals of major diastereomer marked as A, signals of minor diastereomer marked as B: *δ* 194.6, 187.6, 175.3, 156.2, 131.5, 126.65, 115.1, 103.5, 65.5, 62.4, 57.2, 57.08, 35.7, 32.1, 30.6, 26.0, 23.5 ppm; Significant signals minor tautomer: ^13^C NMR (125 MHz, CDCl_3_): Signals of major diastereomer marked as A, signals of minor diastereomer marked as B: *δ* 202.2, 190.0, 169.2, 156.5, 131.8, 126.5, 115.2, 106.3, 66.2, 59.5, 58.0, 57.12, 36.1, 32.4, 31.4, 26.6, 23.4 ppm; IR *ν*_max_ 3283 (w), 2931 (m), 1706 (s), 1654 (s), 1609 (s), 1509 (s), 1436 (m), 1373 (m), 1306 (w), 1239 (s), 1221 (s), 1180 (m), 1113 (w), 1072 (w), 1044 (m), 915 (m) cm^–1^; HRMS (ESI) *m*/*z* [M + H^+^] calcd. for C_19_H_22_NO_5_^+^ 344.14925, found 344.14904.

**(3*S*,6*Z*)-4-Aza-12,13-dibromo-7-hydroxy-15-oxa-5,21-dioxo-tricyclo-[14.2.2.1^3,6^] henicosa-1(18),6,16(17),19-tetraene (9)**. A solution of alkene **16** (500 mg, 1.17 mmol, 1.00 eq) in CCl_4_ (5.3 mL) was treated with bromine (90.3 μL, 1.75 mmol, 1.50 eq) and stirred in a sealed tube for 30 h at 80 °C. The solvent was removed under reduced pressure and the remainder was purified by column chromatography on reversed phase silica gel (RP18, 30% MeCN in H_2_O + 0.1% HCOOH → 35% MeCN in H_2_O + 0.1% HCOOH → 40% MeCN in H_2_O + 0.1% HCOOH → 45% MeCN in H_2_O + 0.1% HCOOH → 50% MeCN in H_2_O + 0.1% HCOOH → 100% MeCN in H_2_O + 0.1% HCOOH) to afford dibromide **9** as a yellow foam and as a mixture of two diastereomers of unknown *dr*. Yield: 228 mg (468 µmol, 40%). *R_f_* = 0.62 (10% MeOH in CH_2_Cl_2_); ^1^H NMR (500 MHz, CDCl_3_) *δ* 8.67/8.42/8.17 (s, 1H, NH), 7.24–6.72 (m, 4H, CH_Ar_), 4.82–3.83 (m, 5H, CH_2_C*H*NH, ArOCH_2_, (CHBr)_2_), 3.39–2.94 (m, 2H, ArCH_2_), 2.41–0.75 (m, 8H, OCC*H*_2_, CHBr(C*H*_2_)_3_) ppm; Major tautomer: ^13^C NMR (125 MHz, CDCl_3_) *δ* 198.3 (CO), 193.3 (COH), 168.5 (HNCO), 157.5 (OC_q,Ar_), 133.0 (CH_2_C*C*H_Ar_), 130.4 (CH_2_C*C*H_Ar_), 126.8 (CH_2_*C*_q,Ar_), 115.6 (OC*C*H_Ar_), 101.6 (NCO*C*CO, HMBC correlation), 70.0 (ArOCH_2_), 62.5 (HCNH), 59.6 ((CHBr)_2_), 54.3 ((CHBr)_2_), 38.2 (*C*H_2_CHBr), 34.9 (CH_2_Ar), 27.0, 21.9 (HOC*C*H_2_, CHBrC*H*_2_(*C*H_2_)_2_) ppm; Significant signals minor tautomer: ^13^C NMR (125 MHz, CDCl_3_): *δ* 168.7, 132.7, 130.9, 62.4, 61.9, 55.0, 26.4/25.5 ppm; IR *ν*_max_ 3241 (m), 2929 (m), 1693 (s), 1652 (s), 1605 (s), 1507 (s), 1460 (m), 1431 (m), 1350 (w), 1299 (w), 1250 (m), 1215 (s), 1177 (m), 1112 (w), 1112 (w), 1040 (w), 1016 (m), 925 (w), 906 (m), 858 (m), 811 (w), 769 (w), 733 (s) cm^–1^; HRMS (ESI) *m*/*z* [M + H^+^] calcd. for C_19_H_22_Br_2_NO_4_^+^ 487.98896, found 487.98884.

**((3*S*,6*Z*)-4-Aza-7,14-dihydroxy-5,18-dioxo-12-vinyl-tricyclo[11.3.1.1^3,6^]octadeca-1(17),6,13(14),15-tetraene (10)**. A solution of alkene **16** (500 mg, 1.17 mmol, 1.00 eq) in degassed diethylaniline (4.7 mL) was stirred in a sealed tube at 190 °C for 42 h. The solution was diluted with EtOAc (50 mL). The organic phase was washed with 2M HCl (2 × 75 mL) and the aqueous phase was extracted with EtOAc (30 mL). The combined organic phases were dried over Na_2_SO_4_ and the solvent was removed under reduced pressure. Purification of the crude product by column chromatography on reversed phase silica gel (RP18, 30% MeCN in H_2_O + 0.1% HCOOH → 40% MeCN in H_2_O + 0.1% HCOOH → 50% MeCN in H_2_O + 0.1% HCOOH → 60% MeCN in H_2_O + 0.1% HCOOH → 100% MeCN in H_2_O + 0.1% HCOOH) afforded **10** as a colourless foam and as an inseparable mixture of two diastereomers. Yield: 218 mg (666 µmol, 57%, *dr* 12.5:1). *R_f_* = 0.28 (10% MeOH in CH_2_Cl_2_); ^1^H NMR (500 MHz, CD_3_OD): Signals of major diastereomer marked as A, signals of minor diastereomer marked as B; diastereotopic H-atoms indicated as a, b: *δ* 6.88–6.72 (m, 2H, C*H_Ar_*CCH_2_), 6.60^A^ (m, 1H, C*H_Ar_*COH), 6.56^B^ (m, 1H, C*H_Ar_*COH), 6.36^B^ (m, 1H, C=CH), 5.95^A^ (ddd, *J* = 16.0, 10.4, 6.2 Hz, 1H, C=CH), 5.00–4.90 (m, 2H, CH_2_=C), 4.00^A^ (t, *J* = 3.4 Hz, 1H, CH_2_C*H*NH), 3.96^B^ (t, *J* = 3.4 Hz, 1H, CH_2_C*H*NH), 3.71 (m, 1H, CHC=C), 3.58^a^ (m, 1H, HOCC*H*), 3.14^A,a^ (dd, *J* = 14.0, 3.5 Hz, ArCH), 3.08^B,a^ (dd, *J* = 14.0, 3.5 Hz, ArCH), 2.78^b^ (dd, *J* = 13.7, 3.5 Hz, 1H, ArCH), 2.13^b^ (dt, *J* = 14.0, 4.0 Hz, 1H, HOCC*H*), 1.99^a^ (m, 1H, OCCH_2_C*H*), 1.67^a^ (m, 1H, CHCH_2_C*H*), 1.55^b^ (m, 1H, CHCH_2_C*H*), 1.45–1.26 (m, 2H, OC(CH_2_)_2_C*H*_2_), 0.89^B,b^ (m, 1H, OCCH_2_C*H*), 0.44^A,b^ (m, 1H, OCCH_2_C*H*) ppm; ^13^C NMR (125 MHz, CD_3_OD): Signals of major diastereomer marked as A, signals of minor diastereomer marked as B: *δ* 198.9 (CO), 188.8 (COH), 176.2 (HNCO), 155.4 (OC_q,Ar_), 144.1^A^ (C=*C*H), 143.1^B^ (C=*C*H), 131.1 (OCCH*C*H_Ar_), 130.9 (CH*C*_q,Ar_), 130.7^A^ (C*C*H_Ar_C), 129.9^B^ (C*C*H_Ar_C), 126.2 (CH_2_*C*_q,Ar_), 115.8^B^ (OC*C*H_Ar_), 115.2^A^ (OC*C*H_Ar_), 113.2^B^ (C=*C*H_2_), 113.0^A^ (C=*C*H_2_), 105.4 (NCO*C*CO), 63.9^A^ (HCNH), 63.4^B^ (HCNH), 40.7 (*C*HC=C), 37.4^A^ (ArCH_2_), 37.3^B^ (ArCH_2_), 35.4^A^ (CHCH_2_*C*H_2_), 32.1^B^ (CHCH_2_*C*H_2_), 29.3^A^ (HOC*C*H_2_), 25.9^B^ (HOC*C*H_2_), 24.9^A^, 24.8^A^ (OC(CH_2_)_2_*C*H_2_, OCCH_2_*C*H_2_), 23.6^B^, 23.4^B^ (OC(CH_2_)_2_*C*H_2_, OCCH_2_*C*H_2_) ppm; IR *ν*_max_ 3294 (m), 2931 (m), 2863 (w), 1969 (w), 1656 (s), 1694 (s), 1511 (m), 1435 (m), 1336 (w), 1252 (m), 1169 (w), 1100 (w), 911 (m) cm^–1^; HRMS (ESI) *m*/*z* [M + H^+^] calcd. for C_19_H_22_NO_4_^+^ 328.15433, found 328.15411.

**(3*S*,6*Z*,8*R*,12*E*)-4-Aza-*N*-(tert-butoxycarbonyl)-7-hydroxy-8-methyl-15-oxa-5,21-dioxo-tricyclo[14.2.2.1^3,6^]henicosa-1(18),6,12,16(17),19-pentaene (18)**. A solution of alkene **17** (263 mg, 596 μmol, 1.00 eq) in EtOAc (6 mL) was treated with Pd on charcoal (26.3 mg, 10 wt%). The resulting suspension was stirred under a H_2_-atmosphere for 31 h at room temperature. The solid was filtered off over Celite^®^ and washed with EtOAc. The combined filtrates were concentrated under reduced pressure to give **18** as an orange foam. Yield: 261 mg (588 µmol, 99%). *R_f_* = 0.93 (10% MeOH in CH_2_Cl_2_); $[\alpha {]}_{D}^{20}\;$+38.8° (c 0.75, MeOH); Major tautomer: ^1^H NMR (500 MHz, CD_3_OD) *δ* 7.02–6.57 (m, 4H), 4.50 (m, 1H), 4.26–4.06 (m, 2H), 3.41 (dd, *J* = 14.4, 3.0 Hz, 1H), 3.37 (m, 1H), 3.08 (dd, *J* = 14.8, 3.0 Hz, 1H), 1.63 (s, 9H), 1.60–1.15 (m, 7H), 1.08 (d, *J* = 6.7 Hz, 3H), 1.03 (m, 1H), 0.73–0.42 (m, 2H) ppm; Significant signals minor tautomer: ^1^H NMR (500 MHz, CD_3_OD) *δ* 4.68 (m, 1H), 3.54 (m,1H) ppm¸ Major tautomer: ^13^C NMR (125 MHz, CD_3_OD) *δ* 193.7, 174.9, 157.4, 150.6, 131.8, 127.9, 116.1, 104.2, 85.2, 67.8, 66.4, 38.3, 35.4, 34.9, 29.4, 28.4, 27.5, 26.1, 24.7, 17.7 ppm; Significant signals minor tautomer: ^13^C NMR (125 MHz, CD_3_OD) *δ* 63.3, 32.8, 30.4, 28.44, 23.7, 14.3 ppm; Major tautomer: ^1^H NMR (500 MHz, CDCl_3_) *δ* 7.06–6.65 (m, 4H), 4.44 (m, 1H), 4.26–4.06 (m, 2H), 3.42 (m, 1H), 3.41 (dd, *J* = 14.6, 3.0 Hz, 1H), 3.12 (dd, *J* = 14.6, 4.0 Hz, 1H), 1.64 (s, 9H), 1.50–1.13 (m, 7H), 1.06 (m, 3H), 1.04 (m, 1H), 0.73–0.42 (m, 2H) ppm; Significant signals minor tautomer: ^1^H NMR (500 MHz, CDCl_3_) *δ* 4.62 (m, 1H), 3.55 (m, 2H), 1.60 (s, 9H) ppm; Major tautomer: ^13^C NMR (125 MHz, CDCl_3_) *δ* 195.6, 191.6, 174.1, 155.8, 149.0, 132.6, 129.3, 126.6, 117.6, 117.0, 102.8, 84.4, 67.0, 65.5, 36.5, 34.6, 34.1, 28.36, 28.3, 26.1, 24.9, 23.5, 17.6 ppm; Significant signals minor tautomer: ^13^C NMR (125 MHz, CDCl_3_) *δ* 131.1, 130.7, 116.5, 115.4, 62.2, 36.6, 34.9, 33.8, 28.4, 28.0, 26.4, 25.8, 25.4, 23.8, 16.6 ppm; IR *ν*_max_ 2971 (m), 2935 (m), 2233 (w), 2078 (m), 1740 (s), 1611 (s), 1509 (m), 1440 (m), 1354 (s), 1302 (m), 1256 (w), 1228 (s), 1218 (s), 1156 (m), 1116 (s), 972 (s) cm^–1^; HRMS (ESI) *m*/*z* [M + Na^+^] calcd. for C_25_H_33_NO_6_Na^+^ 466.22001, found 466.21899.

**(3*S*,6*Z*,8*R*)-4-Aza-7-hydroxy-8-methyl-15-oxa-5,21-dioxo-tricyclo[14.2.2.1^3,6^] henicosa-1(18),6,16(17),19-tetraene (3)**. To a solution of tetramic acid **18** (227 mg, 512 μmol, 1.00 eq) in CH_2_Cl_2_ (9.5 mL) was added TFA (950 μL). After stirring for 15 min at room temperature, toluene (100 mL) was added and the solvent was removed under reduced pressure. Toluene (50 mL) was added again and the solvent was removed, which afforded **3** as an orange foam. Yield: 176 mg (512 µmol, quant.). *R_f_* = 0.51 (10% MeOH in CH_2_Cl_2_); $[\alpha {]}_{D}^{20}$–17.7° (c 0.74, MeOH); Major tautomer: ^1^H NMR (500 MHz, CD_3_OD): diastereotopic H-atoms indicated as a, b: *δ* 7.14 (m, 1H, C*H_Ar_*CCH_2_), 6.96 (m, 1H, C*H_Ar_*CCH_2_), 6.78 (m, 2H, CH_Ar_CO), 4.23–4.07 (m, 3H, CHN, ArOCH_2_), 3.33 (m, 1H, CHMe), 3.07^a^ (dd, *J* = 14.3, 4.1 Hz, 1H, ArCH), 2.97^b^ (dd, *J* = 14.4, 2.4 Hz, 1H, ArCH^b^), 1.52 (m, 2H, OCH_2_C*H*_2_), 1.44–1.16 (m, 5H, CHMeC*H*^a^, CMeCH_2_C*H*_2_, O(CH_2_)_2_C*H*_2_), 1.08^b^ (m, 1H, CHMeC*H*), 1.05 (d, *J* = 6.9 Hz, 3H, CH_3_), 0.68–0.40 (m, 2H, CMe(CH_2_)_2_C*H*_2_) ppm; Significant signals minor tautomer: ^1^H NMR (500 MHz, CD_3_OD) *δ* 6.64 (m, 2H), 3.53 (m, 1H) ppm; Major tautomer: ^13^C NMR (125 MHz, CD_3_OD) *δ* 198.2 (CO, HMBC correlation), 190.2 (COH), 177.5 (HNCO, HMBC correlation), 157.0 (OC_q,Ar_), 133.3 (C*H_Ar_*CCH_2_), 130.8 (C*H_Ar_*CCH_2_), 128.1 (CH_2_*C*_q,Ar_), 118.2 (OC*C*H_Ar_), 117.4 (OC*C*H_Ar_), 103.2 (NCO*C*CO), 67.6 (ArOCH_2_), 63.6 (CHN), 37.3 (CHMe), 36.4 (ArCH_2_), 35.2 (CMe*C*H_2_), 29.5 (CMeCH_2_*C*H_2_), 27.3 (CMe(CH_2_)_2_*C*H_2_), 25.9 (OCH_2_*C*H_2_), 24.7 (O(CH_2_)_2_*C*H_2_), 17.8 (CH_3_) ppm; Significant signals minor tautomer: ^13^C NMR (125 MHz, CD_3_OD) *δ* 131.9, 116.0 ppm; Major tautomer: ^1^H NMR (500 MHz, CDCl_3_) *δ* 7.19–7.00 (m, 2H), 6.79 (m, 2H), 4.35–4.09 (m, 3H), 3.38 (m, 1H), 3.28–2.83 (m, 2H), 1.52 (m, 2H), 1.44–1.12 (m, 5H), 1.12 (m, 1H), 1.07 (d, *J* = 6.8 Hz, 3H), 0.67–0.41 (m, 2H) ppm; Significant signal minor tautomer: ^1^H NMR (500 MHz, CD_3_OD) *δ* 3.63 (m, 1H) ppm; Major tautomer: ^13^C NMR (125 MHz, CDCl_3_) *δ* 194.3, 192.1, 175.9, 155.7, 132.6, 129.6, 126.4, 117.3, 116.7, 102.0, 66.8, 62.2, 36.0, 34.4, 28.4, 26.0, 24.9, 23.6, 17.6 ppm; Significant signals minor tautomer: ^13^C NMR (125 MHz, CDCl_3_) *δ* 130.5, 116.3, 115.9, 66.9, 63.7, 36.4, 36.1, 29.4, 25.9, 25.1, 23.9, 17.1 ppm; IR *ν*_max_ 2929 (m), 2853 (m), 1654 (s), 1608 (s), 1509 (s), 1456 (m), 1340 (m), 1259 (m), 1222 (m), 1174 (m) cm^–1^; HRMS (ESI) *m*/*z* [M + H]^+^ calcd. for C_20_H_26_NO_4_^+^ 344.18563, found 344.18481.

### 4.3. Experimental Design and Evaluation of Herbicidal Tests

The test compounds were applied as solutions in a mixture of isopropanol/water = 1:1 + 0.25% Tween 20 to arrays of two plant species (dandelions and thistle) that are susceptible to natural macrocidins. Plants were grown in 100 cm^2^ pots with two individuals/pot and four replicates/treatment. Within each species there were three treatments: untreated control, the macrocidinoids (100 or 150 mM) and the commercial herbicide diflufenican (1.2 mM). Spraying was done with 0.4 mL/100 cm^2^. Effects were assessed after 14 days and a second time after three to six weeks when mortality was fully developed. A mortality factor, i.e., the percentage of eventually dead plants, was calculated according to the Henderson-Tilton method [22] using the formula:

Mortality [%] = (1 − T_a_ × C_b_/T_b_ × C_a_) × 100

with T_b_ = number of plants before treatment (=8); T_a_ = number of vital, treated plants at the end of observation period; C_b_ = number of vital, untreated control plants at beginning (=8); C_a_ = number of vital, untreated control plants at the end of observation period.

### 4.4. Antimicrobial Activity

The antibacterial activities were determined by the so-called broth microdilution method [23]. In brief: all cultivations were done in standard microbiological media such as TSB medium (tryptic soy broth) for *S. aureus* (SH1000) and LB medium (lysogeny broth) for *Escherichia coli* (ATCC25922) and at 37 °C (only *A. baumannii* was cultivated at 30 °C). The overnight cultures of the bacterial test strains were diluted to an OD_600_ of 0.1 and further incubated until an OD_600_ = 0.5 was reached. These cultures were used as working cultures. They were diluted to obtain an OD_600_ of 0.1, determined in 45 µL of the bacterial suspension in each well of a 384-well plate, or in 90 µL of the bacterial suspension in each well of a half-area 96-well plate. Compound solutions were prepared in separate 96-well compound plates starting from 10 mM stock solutions in DMSO. The compound concentrations were adjusted so that the maximum DMSO concentrations in the assay plates were 1%, assuring no interference with growth from the solvent. The respective volumes of the compound solutions were added to the microbial suspensions with the 96-channel semi-automated pipettor CyBio Selma (Analytik Jena). The OD_600_ was determined directly after compound addition and subsequently after 1, 3 and 24 h using the Epoch 2 microplate reader (BioTek Instruments).

### 4.5. Antibiofilm Activity

*Staphylococcus aureus* DSM 1104 from a stock kept at −20 °C was precultured in 25 mL CASO (casein-peptone soymeal-peptone) medium in a 250 mL flask at 37 °C and shaken (100 rpm, 20 h. The OD_600_ of the culture solution was adjusted to 0.001 McFarland standard. The solution was incubated in 96-well microtiter plates (TPP tissue culture ref. No. 92196) for 18 h at 37 °C with 150 μL of serially diluted test compounds (250–2 μg/mL) in CASO with 4% glucose broth. Compounds showing high activities (e.g., **2**, **3**, **9**) were diluted in the range of 10–0.3 μg/mL. The inhibition of biofilm formation was evaluated by staining with 150 μL of 0.1% crystal violet (CV; Thermo Fisher, Waltham, MA, USA) following previously established protocols [21,24]. Briefly, the supernatant of the 96-well plate was discarded and the wells were washed once with PBS buffer. The remaining biofilms were stained with 0.1% CV at room temperature for 15 min, washed three times with PBS buffer, and finally dissolved in 150 μL ethanol (95%). The absorbance of the resulting solution at 530 nm was quantified using a plate reader (Synergy 2, BioTek, Santa Clara, CA, USA). Methanol (2.5%) and microporenic acid A [21] (250–2 μg/mL) were used as negative and positive controls. Standard deviations (SD) of two repeats with duplicates were 10% or less. Effects on the biofilms and SD values are shown in Table S1 in the Supporting Information (SI).

The precultured bacterial suspension of *S. aureus* strain DSM 1104 was adjusted to 0.001 McFarland standard at OD_600_ and incubated in 96-well tissue microtiter plates for 18 h in 150 μL CASO with 4% glucose broth. The supernatant of the 96-well plate was removed and the remainder was washed with 150 μL PBS buffer. The test compounds were serially diluted in 150 μL of fresh media (CASO with 4% glucose broth) to concentrations of 250–2 μg/mL, and added to the wells. The plates were incubated for a further 24 h at 37 °C. Staining of the preformed biofilm and of the controls was carried out as described above [21,24]. The SD of two repeats with duplicates were 15% or less. SD values are shown in Table S1 in the ESI.

*Candida albicans* DSM 11225 was taken from a −20 °C stock and precultured in 25 mL YPED (Yeast extract Peptone Dextrose) medium in a 250 mL flask at 30 °C and shaken (100 rpm, 18 h). The OD_600_ of the culture solution was adjusted to 0.05 McFarland standard in RPMI 1640 medium. 150 μL of the solution was added to 96-well non-tissue microtiter plates (Falcon non-tissue plate ref. No. 351172) for 90 min at 37 °C, and shaken with 150 rpm. The supernatant was discarded and the residue washed twice with PBS buffer. The test compounds were serially diluted in 150 μL of fresh medium (RPMI 1640) to concentrations of 250–2 μg/mL and added to the wells. Methanol (2.5%) was used as the negative control. The plates were further incubated at 37 °C and shaken (150 rpm, 24 h). The supernatant of the 96-well plate was discarded, the wells were washed once with PBS buffer, and the biofilms were stained with 150 μL of 0.1% CV at room temperature for 25 min and then washed four times with PBS buffer. The biofilms were dissolved in 150 μL ethanol (95%), and the absorbance of the resulting solution at 610 nm was finally quantified using a plate reader (Synergy 2, BioTek, Santa Clara, CA, USA). SD of two repeats with duplicates each were 10% or less. Dispersal effects on preformed biofilms and SD values are shown in Table S1 (SI).

*P. aeruginosa* (PA 14) was cultured in 25 mL LB medium (Luria-Bertani Broth) in a 250 mL flask at 37 °C, shaken with 100 rpm, for 18 h. The OD_600_ of the culture solution was adjusted to 0.025 McFarland standard in LB medium. The test compounds were diluted in 100 μL bacterial solution to concentrations of 250–2 μg/mL and the resulting solutions were added to 96-well plates in an MBEC Innovatech incubator (MBEC Assay^®^, Edmonton, AB, Canada). The plates were incubated at 37 °C at 150 rpm for 24 h. The biofilms were established on the pegs under the growth conditions. The pegs and plates were rinsed once with PBS buffer, the biofilms on pegs were stained with 150 μL 0.1% CV at room temperature for 15 min and then rinsed twice with PBS buffer. The pegs were transferred into a new plate with 150 μL ethanol (95%) and the absorbance at 550 nm was quantified using a plate reader (Synergy 2, BioTek, Santa Clara, CA, USA). Myxovalargin A and methanol (2.5%) were used as positive and negative control.

### 4.6. Cytotoxicity

The cytotoxic effect upon treatment with macrocidinoids **2**, **3**, **6**–**10** for 72 h was determined by standard MTT assays [25]. The tetrazolium salt 3-(4,5-dimethylthiazol-2-yl)-2,5-diphenyltetrazolium bromide (MTT; abcr) is reduced by viable cells to a violet, water-insoluble formazan. Human 518A2 melanoma cells, colon carcinoma cells HCT-116^wt^ and HCT-116^p53−/−^, and KBV cervix carcinoma cells as well as hybrid endothelial EaHy cells (5 × 10^4^ cells mL^−1^, 100 µL/well), were seeded in 96-well tissue culture plates and cultured for 24 h at 37 °C, 5% CO_2_ and 95% humidity. After treatment with the test compounds (stock solutions 10 mM in DMSO and freshly diluted appropriately with sterile Milli-Q water) incubation of cells was continued for 72 h. Blank and solvent controls were treated identically. After addition of a 5 mg mL^−1^ MTT stock solution in phosphate buffered saline (PBS), microplates were incubated for 2 h at 37 °C, centrifuged at 300 g, 4 °C for 5 min and the supernatant was discarded. The precipitate of formazan crystals was then redissolved in a 10% (*w*/*v*) solution of sodium dodecylsulfate (SDS; Carl Roth) in DMSO containing 0.6% (*v*/*v*) acetic acid. To ensure complete dissolution of the formazan, the microplates were incubated for at least 1 h in the dark. Finally, the absorbance at λ = 570 and 630 nm (background) was measured using a microplate reader (Tecan F200). All experiments were carried out in quadruplicate and the percentage of viable cells was calculated as the mean ± SD with controls set to 100%. The determined IC_50_ (inhibitory concentration) values are shown in Table S2 (*cf*. SI).

## Data Availability

The datasets used and/or analyzed during the current study are available from the corresponding author upon reasonable request.

## Supplementary Materials

The following are available online at https://www.mdpi.com/article/10.3390/antibiotics10081022/s1. Figures S1–S67: ^1^H and ^13^C NMR spectra of **3**–**10**, **12**–**16**, **18** and HPLC spectra of **3**–**10**. Figure S68: herbicidal effects on pot plants. Figure S69: growth inhibitory effects of macrocidin derivatives on *E. coli* ΔTolC cultures; Figure S70: growth inhibitory effects of macrocidin derivatives on *Staphylococcus aureus* (SH1000) cultures. Figure S71: growth inhibitory effects of selected macrocidin derivatives on *Actinetobacter baumannii* cultures. Figure S72: dispersal effects on preformed biofilms of *C. albicans*. Table S1: antibacterial effects of compounds **2**–**10** on *E. coli* ΔTolC and *S. aureus*. Table S2: *S. aureus* and *C. albicans* biofilm growth inhibition and dispersion data. Table S3: IC_50_ values for human cells.

## Abbreviations

DMAP: dimethylaminopyridine; DMSO: dimethylsulfoxide; EDC: 1-ethyl-3-(3-dimethylaminopropyl)carbodiimide; NBS: *N*-bromosuccinimide; NMO: *N*-methylmorpholine-*N*-oxide; PBS: phosphate-buffered saline; TFA: trifluoracetic acid; THF: tetrahydrofuran; sat.: saturated.
