# Supplementary material for: Dual Agents: Fungal Macrocidins and Synthetic Analogues with Herbicidal and Antibiofilm Activities
Source: Antibiotics (Basel). 2021 Aug 23;10(8):1022. doi: 10.3390/antibiotics10081022 (PMC8388955; doi:10.3390/antibiotics10081022)
Supplement: Supplementary file 1 [file antibiotics-10-01022-s001.zip › antibiotics-1336664-supplementary.pdf]

Supporting Information

Dual agents: fungal macrocidins and synthetic analogues with herbicidal and antibiofilm activities

Laura Treiber[1], Christine Pezolt[1], Haoxuan Zeng[2,3], Hedda Schrey[2,3], Stefan Jungwirth[4], Aditya Shekhar[4], Marc Stadler[2,3], Ursula Bilitewski[4], Maike Erb-Brinkmann[5], Rainer Schobert[1,*]

[1] *Department of Chemistry, University Bayreuth, Universitaetsstrasse 30, 95440 Bayreuth, Germany*
[2] *Department of Microbial Drugs, Helmholtz Centre for Infection Research GmbH, Inhoffenstrasse 7, 38124 Braunschweig, Germany*
[3] *Institute of Microbiology, Technische Universität Braunschweig, Spielmannstraße 7, 38106 Braunschweig, Germany*
[4] *Compound Profiling and Screening, Helmholtz Centre for Infection Research GmbH, Inhoffenstrasse 7, 38124 Braunschweig, Germany*
[5] *Phytosolution, Querfurter Strasse 9, 06632 Freyburg, Germany*

Table of contents *pages*

NMR spectra of compounds **3–10**, **12-16**, **18** and HPLC chromatograms of compounds **3-10** (Figures S1-S67) …………………………………………………………………….. S1-35

Herbicidal effects on pot plants (Figure S68) S35

Growth inhibitory effects of macrocidin derivatives on *E. coli* ΔTolC cultures (Figure S69) S36

Growth inhibitory effects of macrocidin derivatives and established antibiotics on *Staphylococcus aureus* (SH1000) cultures (Figure S70) S36

Growth inhibitory effects of selected macrocidin derivatives on *Acinetobacter baumannii* cultures (Figure S71) S36

Dispersal effects on preformed biofilms of *C. albicans* (Figure S72) ………………………..... S37

Antibacterial effects of compounds **2-10** on *E. coli* ΔTolC and *S. aureus* (Table S1) S37

S. aureus and *C. albicans* biofilm growth inhibition and dispersion data (Table S2) S38

IC_{50} values for human cells (Table S3) ....……………………… S39

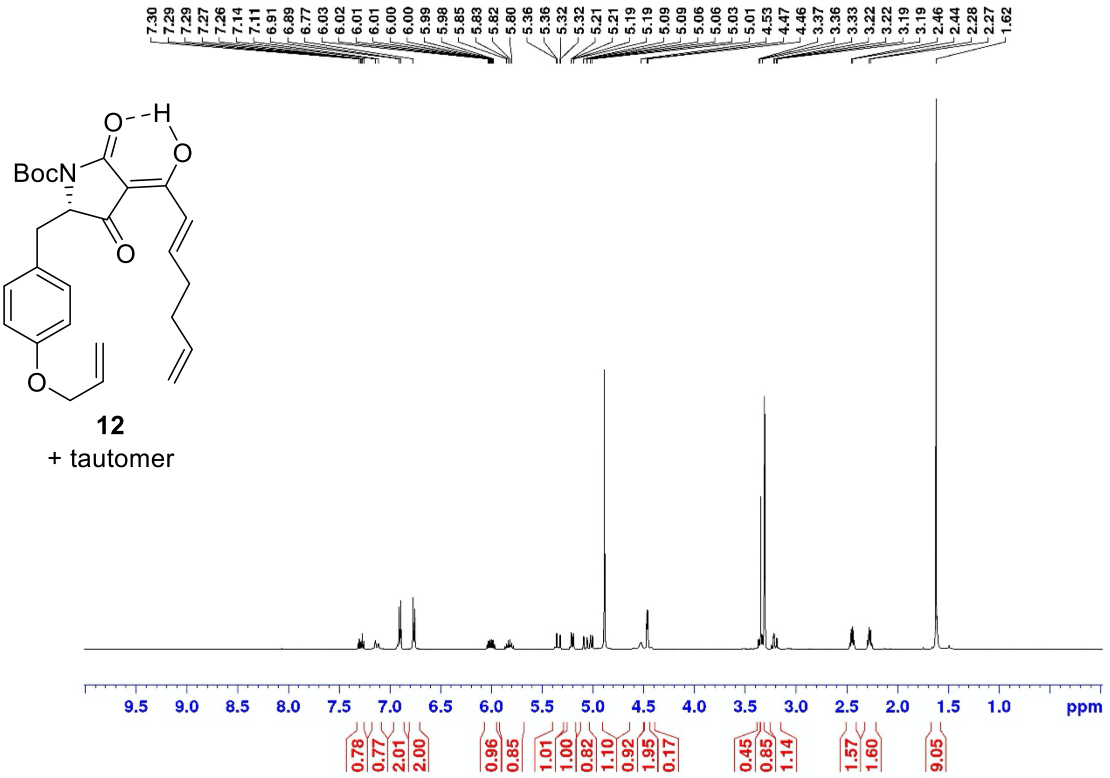

Figure S1. ^{1}H-NMR spectrum of compound **12** in CD_3OD.

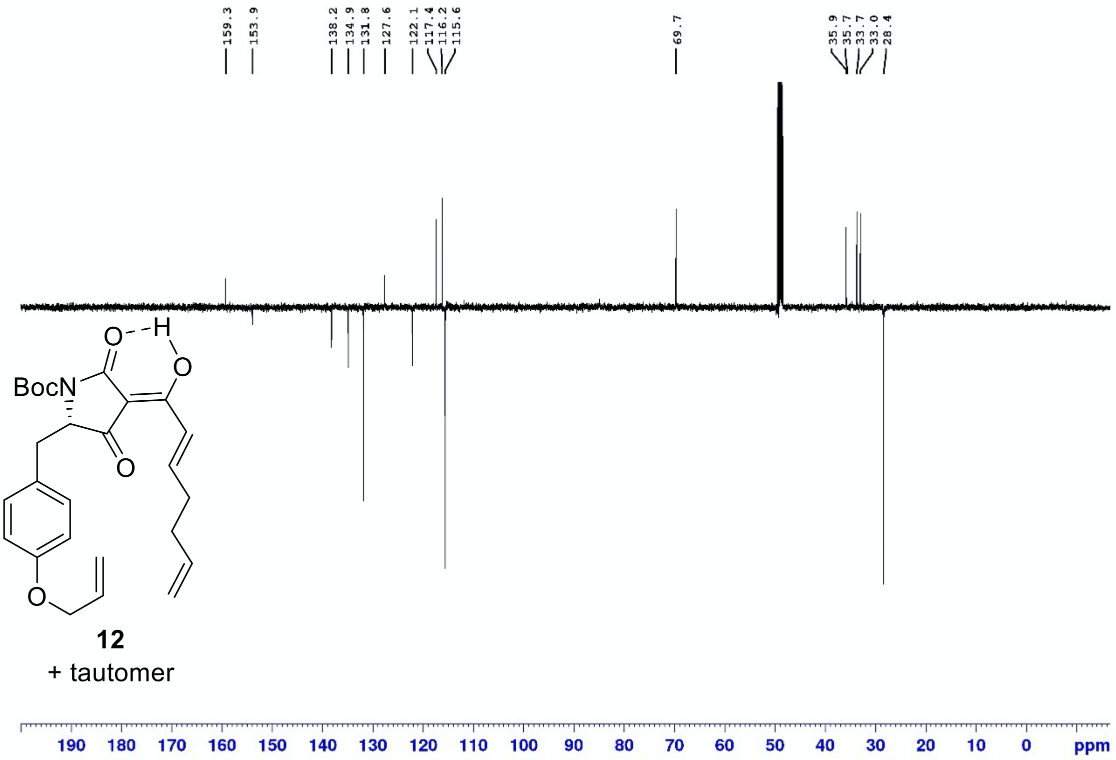

Figure S2. ^{13}C-NMR spectrum of compound **12** in CD_3OD.

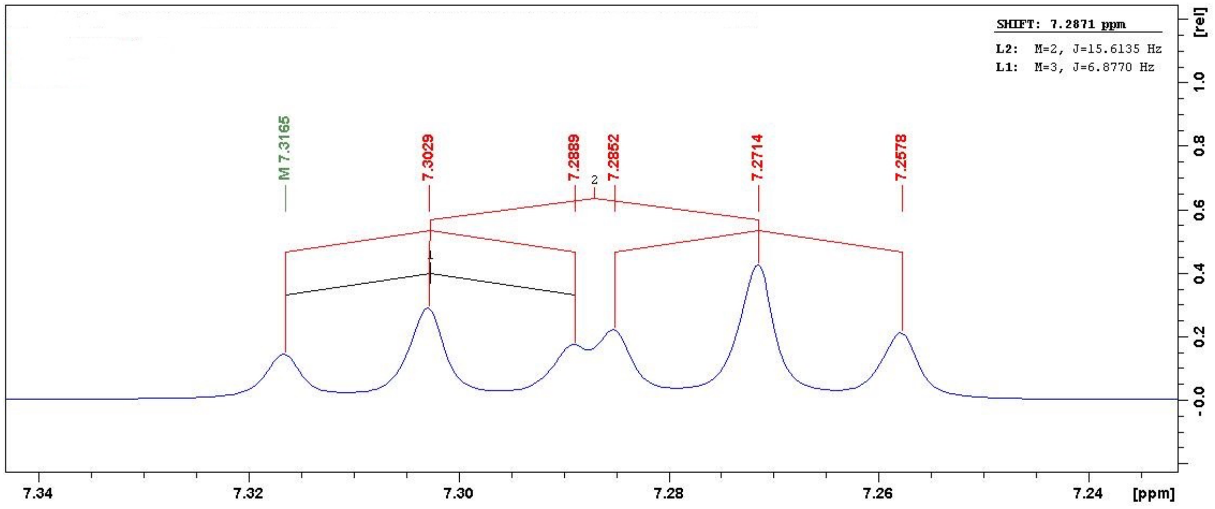

Figure S3. Part of ^{1}H-NMR spectrum of compound **12** in CD_3OD with defined multiplet and coupling constants (J =15.6, 6.9 Hz) that prove the (E)-configuration of the resulting double bond.

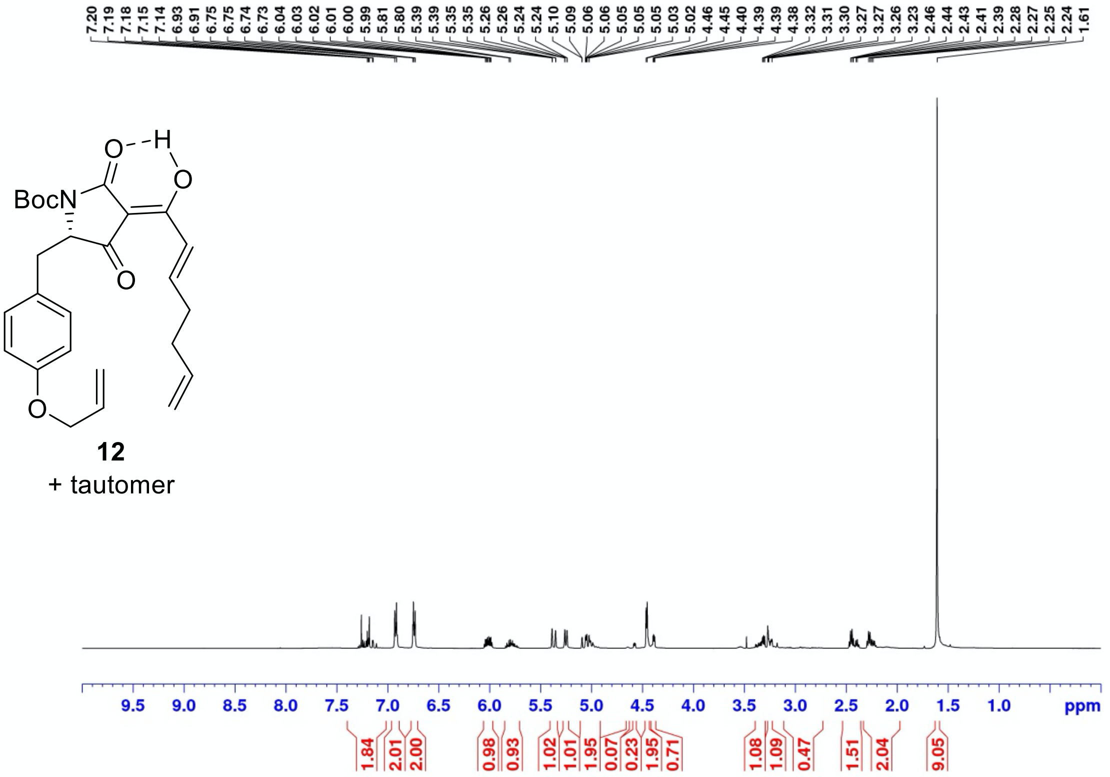

Figure S4. ^{1}H-NMR spectrum of compound **12** in $CDCl_3$.

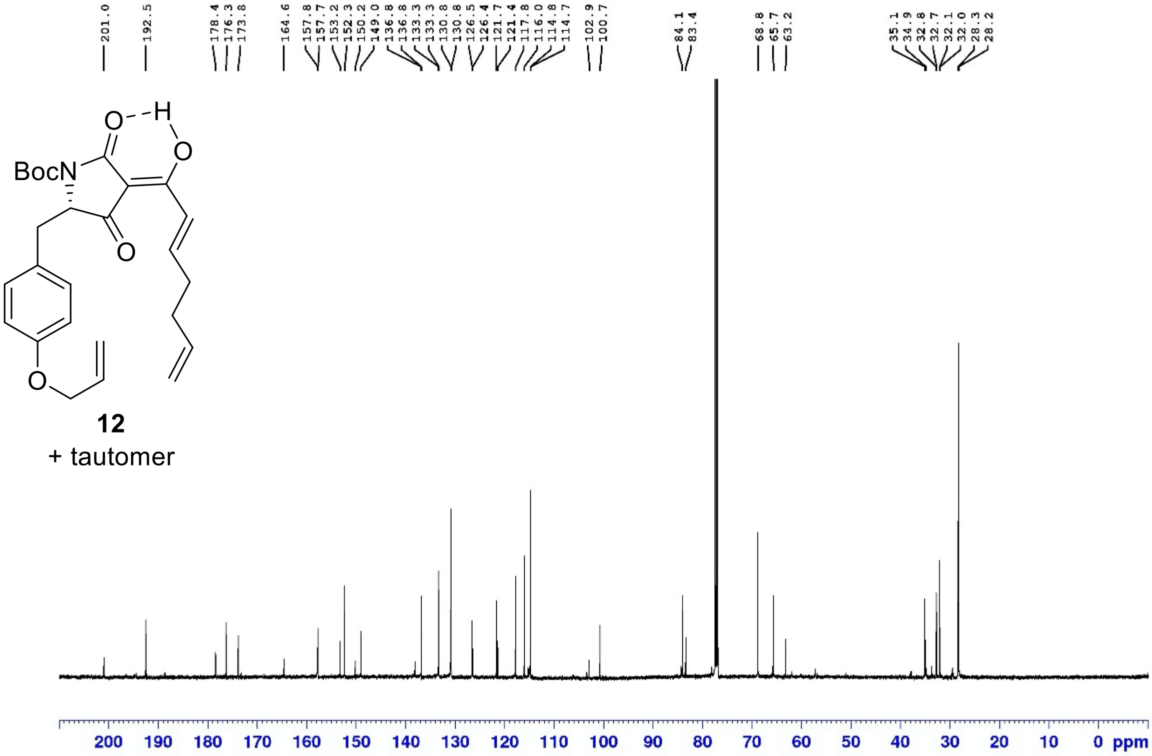

Figure S5. ^{13}C-NMR spectrum of compound **12** in $CDCl_3$.

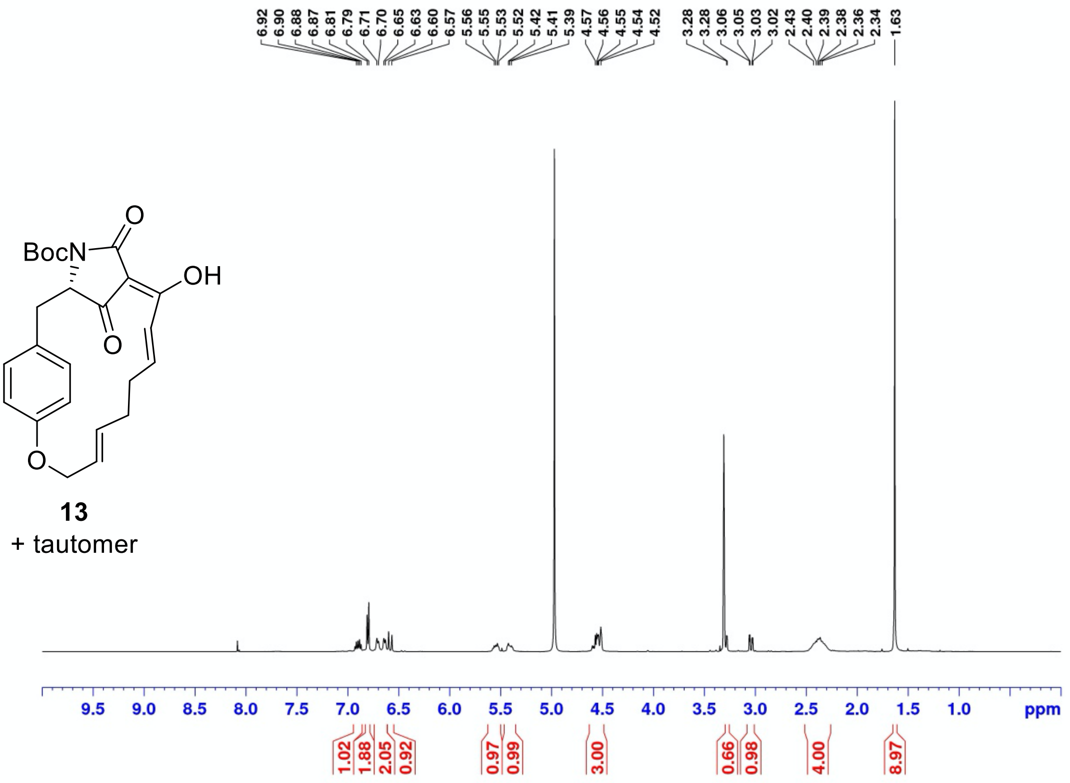

Figure S6. ^{1}H-NMR spectrum of compound **13** in CD_3OD.

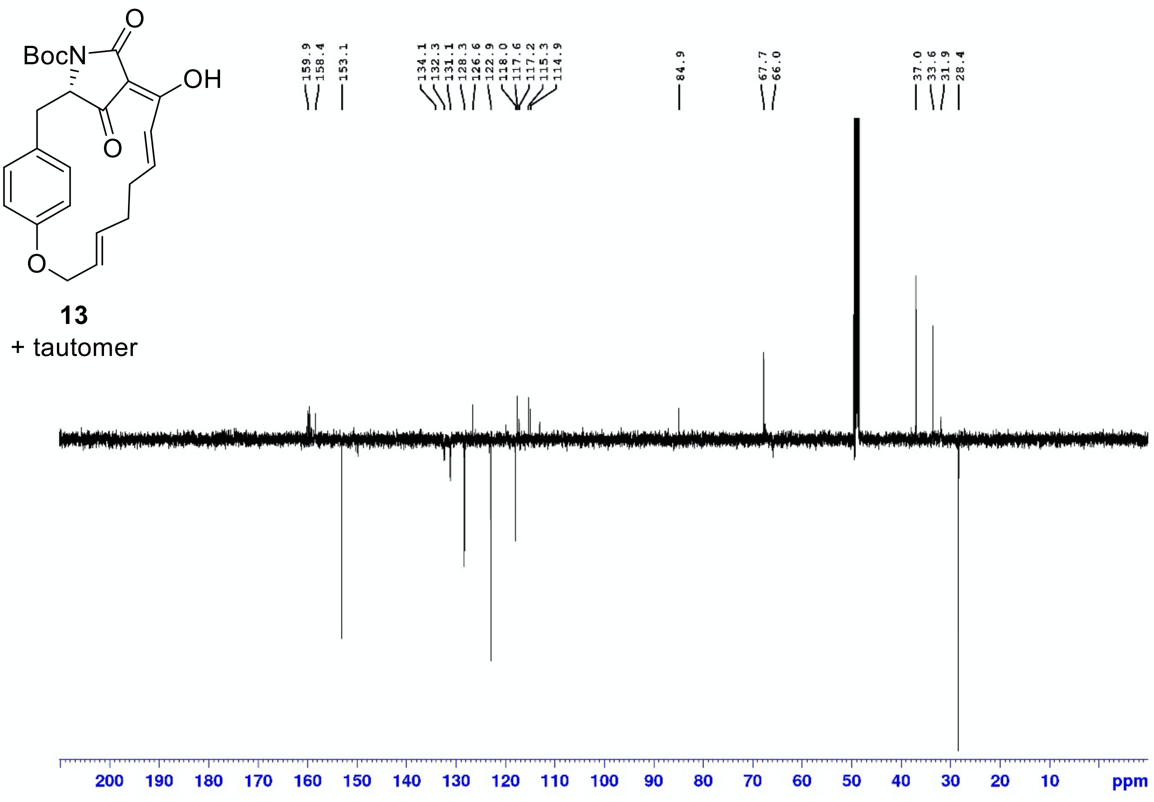

Figure S7. ^{13}C-NMR spectrum of compound **13** in CD_3OD.

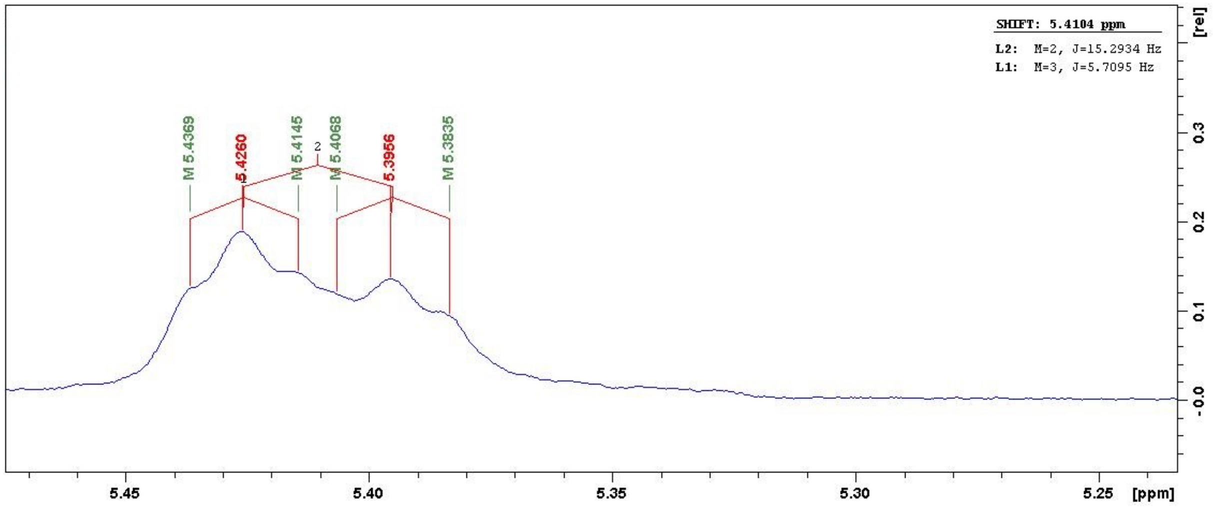

Figure S8. Part of ^{1}H-NMR spectrum of compound **13** in CD_3OD with defined multiplet and coupling constants (J =15.3, 5.7 Hz) that prove the (E)-configuration of the resulting isolated double bond.

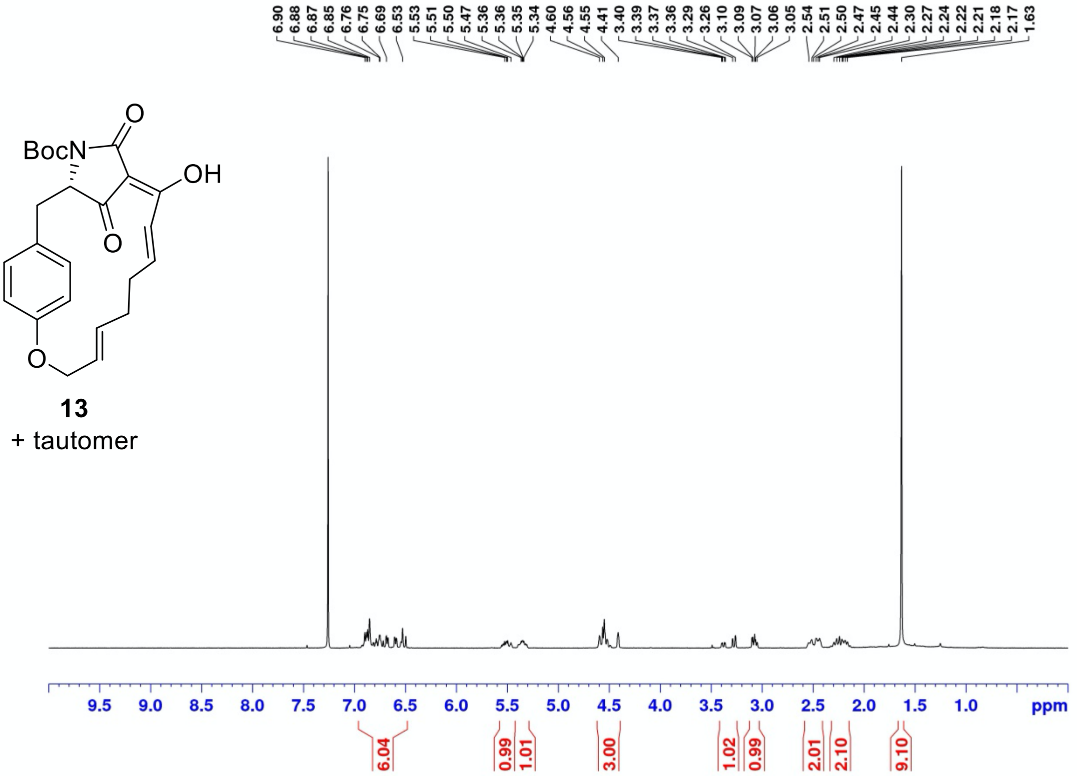

Figure S9. ^{1}H-NMR spectrum of compound **13** in $CDCl_3$.

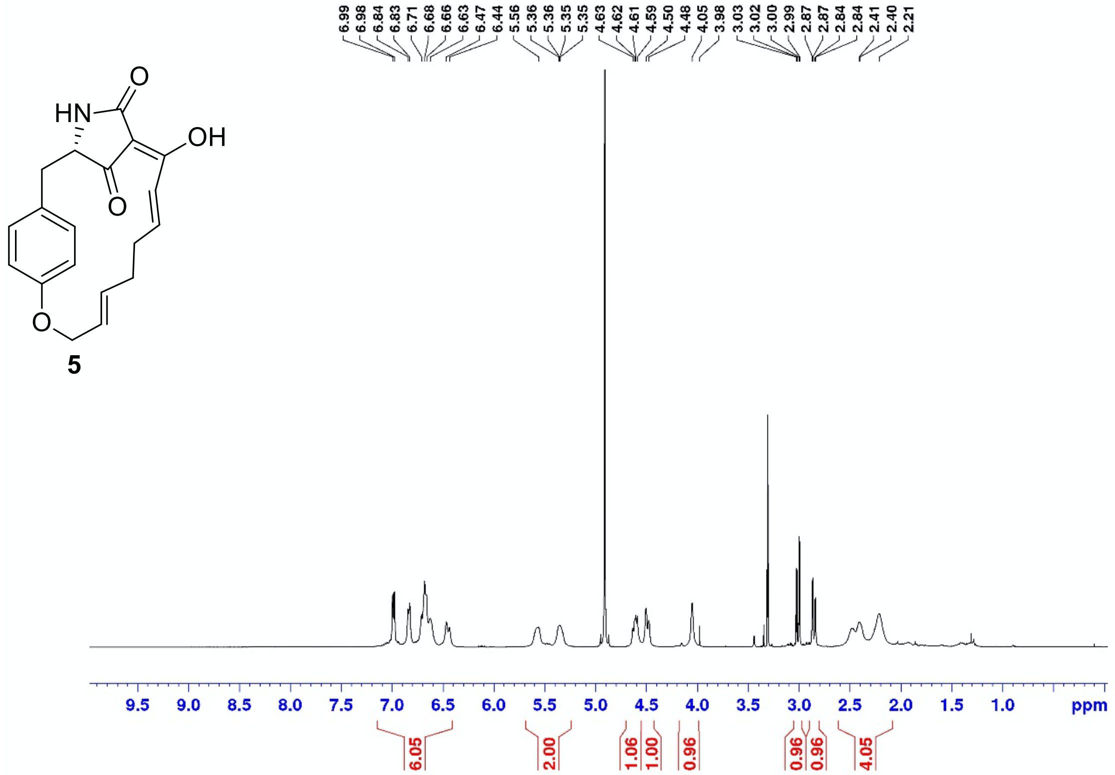

Figure S10. ^{1}H-NMR spectrum of compound **5** in CD_3OD.

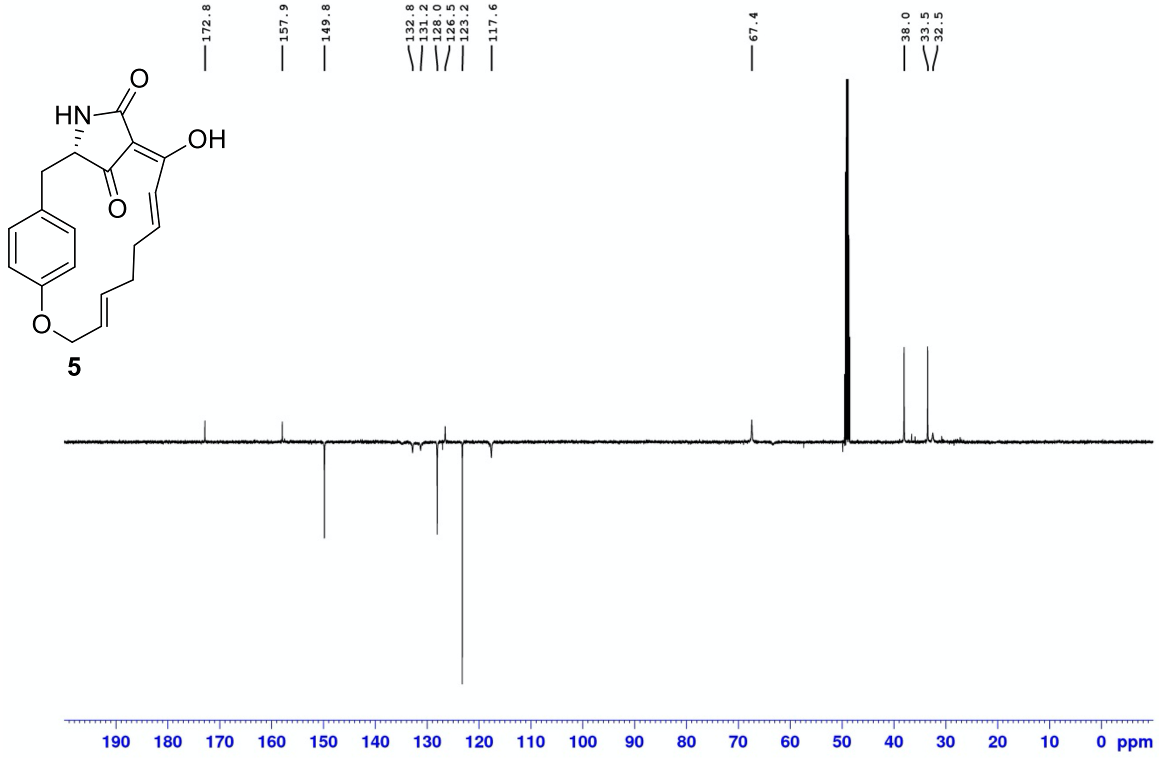

Figure S11. ^{13}C-NMR spectrum of compound **5** in CD_3OD.

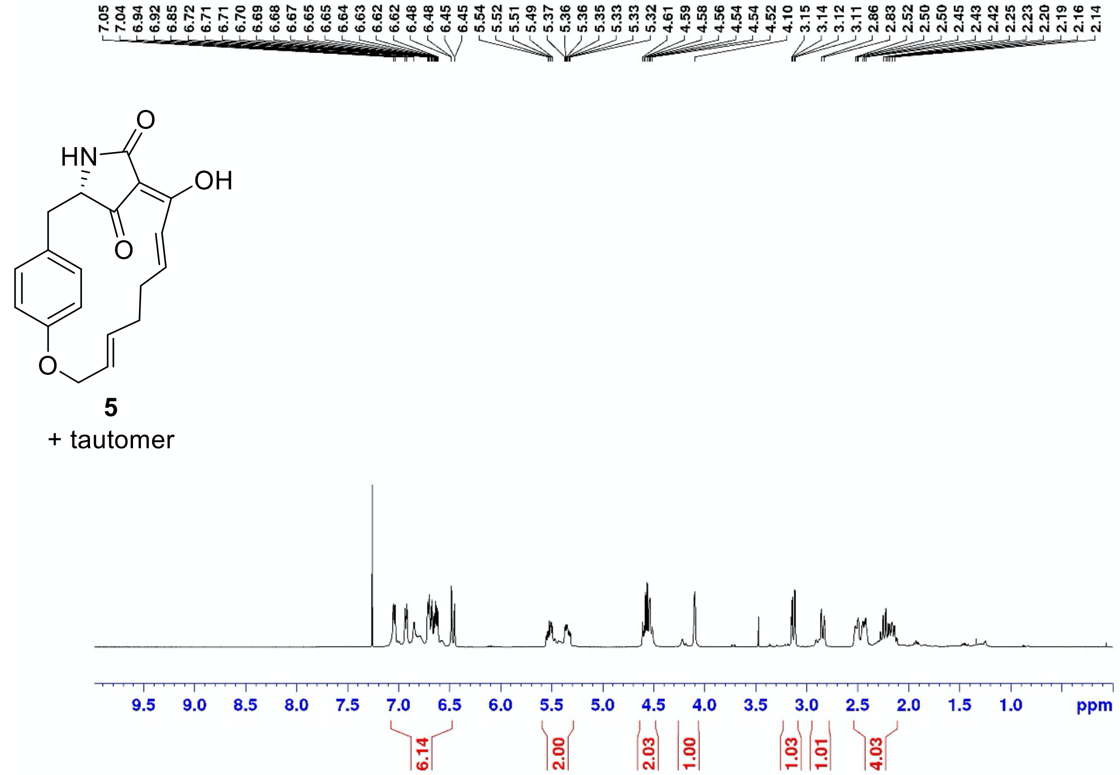

Figure S12. ^{1}H-NMR spectrum of compound **5** in $CDCl_3$.

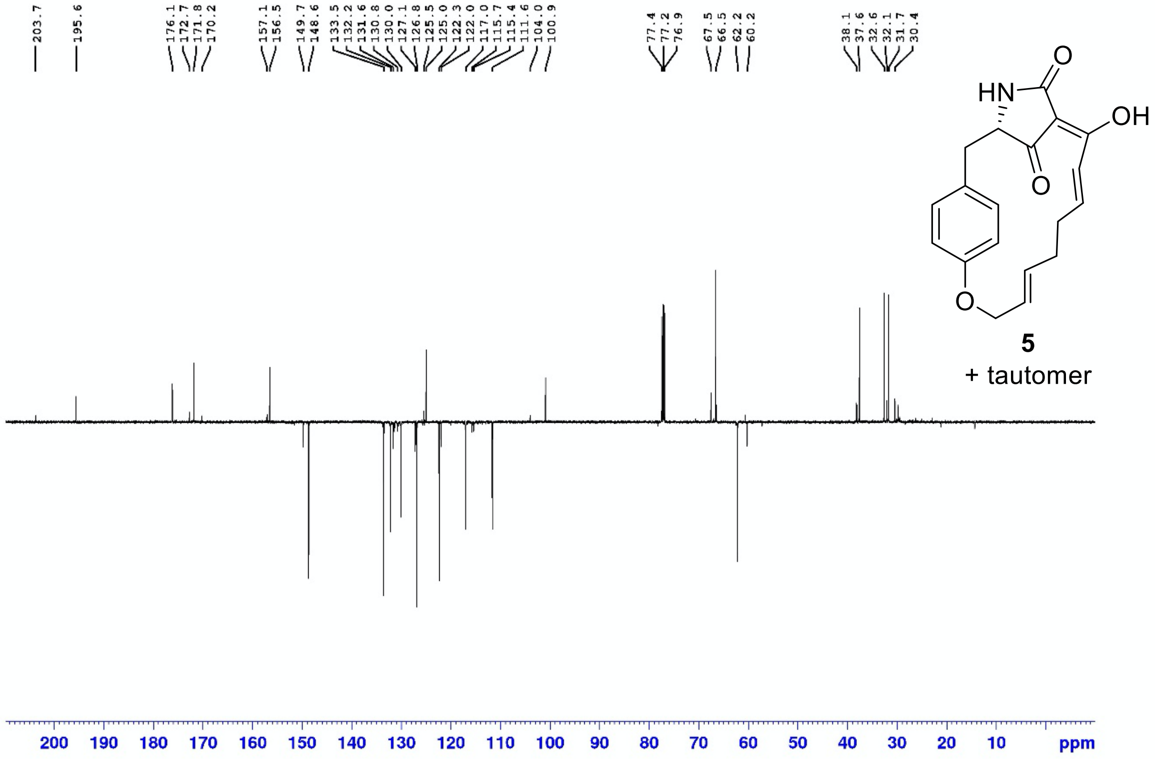

Figure S13. ^{13}C-NMR spectrum of compound **5** in $CDCl_3$.

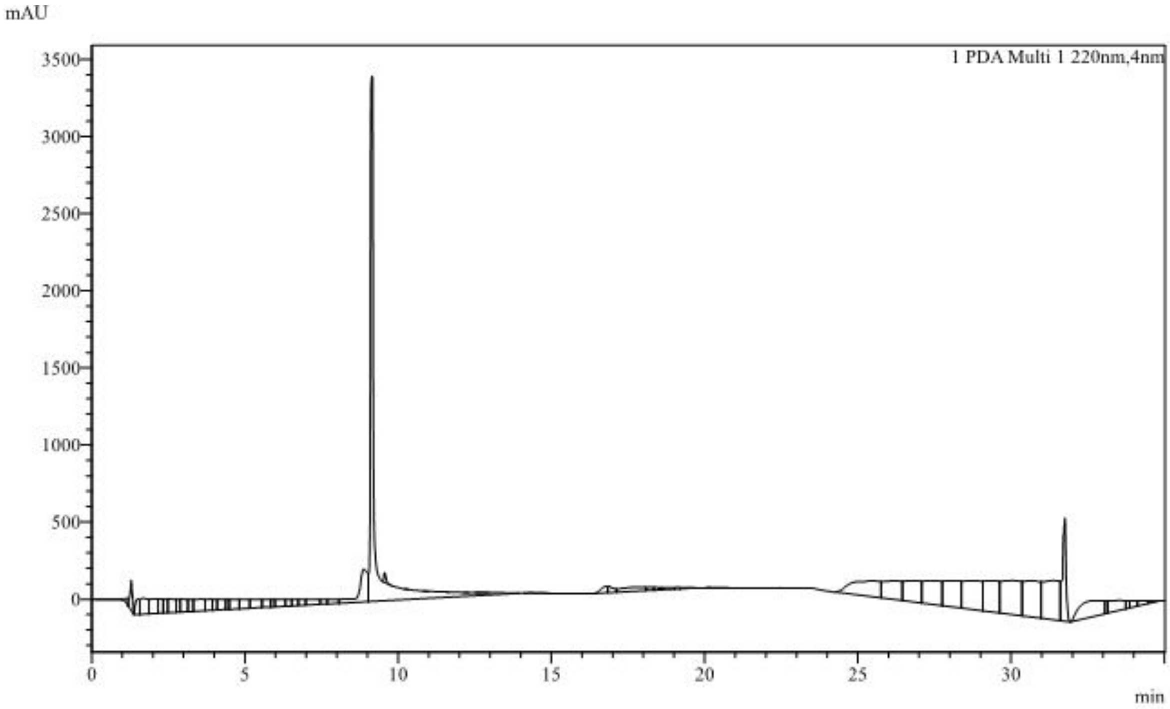

Figure S14. Chromatogram of compound **5**. HPLC: *Shimadzu Nexera XR,* Autosampler *SIL-20A*, diode array detector *SPD-M20A*, C18-column (150 × 4 mm). Method: 40% MeCN in H_2O + 0.1% HCOOH → 60% MeCN in H_2O + 0.1% HCOOH → 80% MeCN in H_2O + 0.1% HCOOH → 97% MeCN in H_2O + 0.1% HCOOH, flow: 1.0 mL/min.

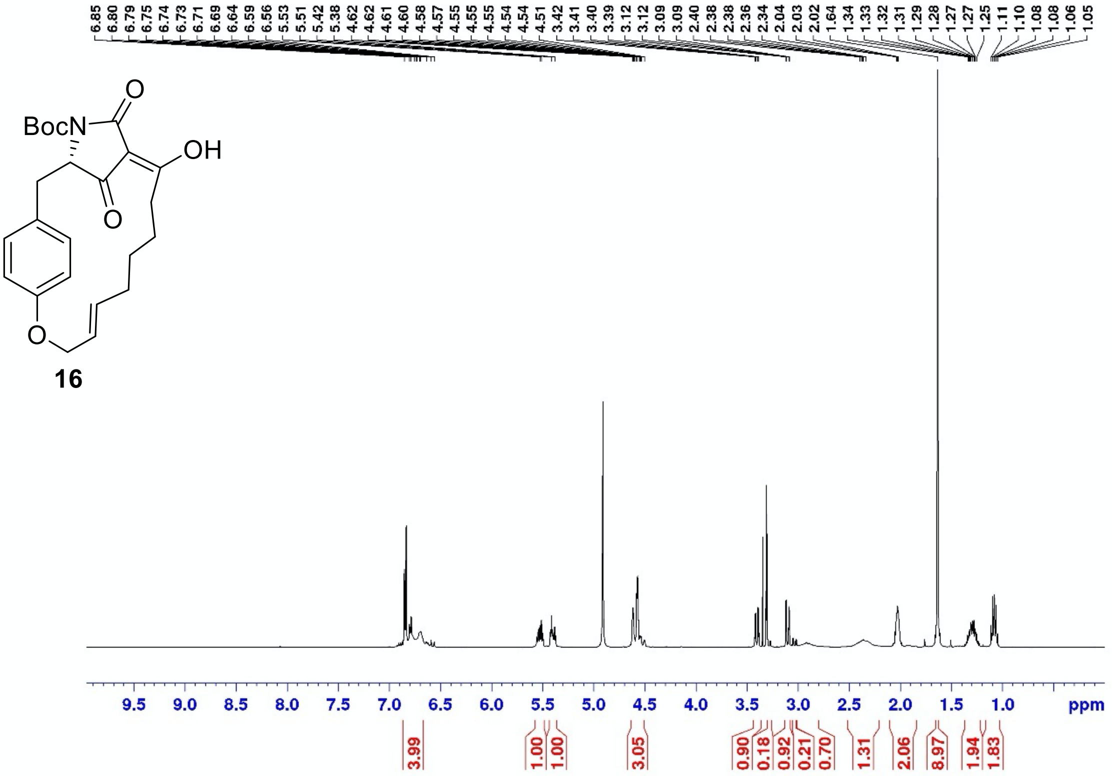

Figure S15. ^{1}H-NMR spectrum of compound **16** in CD_3OD.

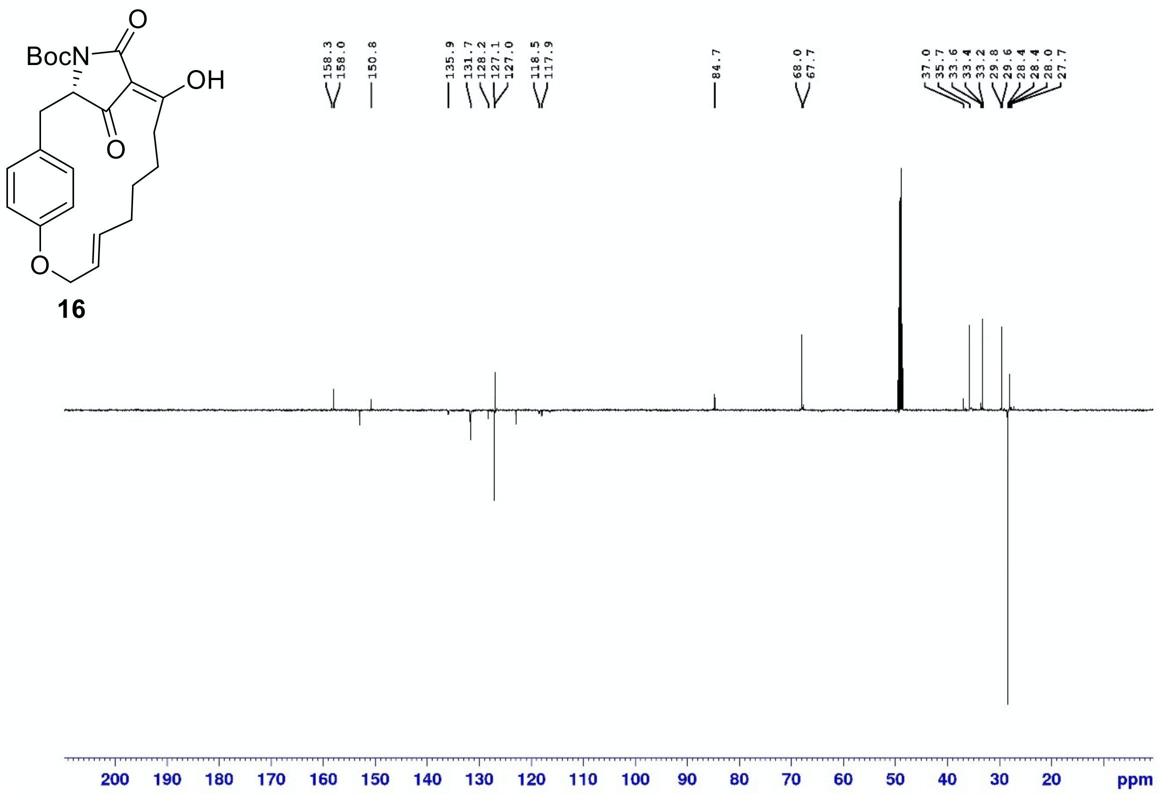

Figure S16. ^{13}C-NMR spectrum of compound **16** in CD_3OD.

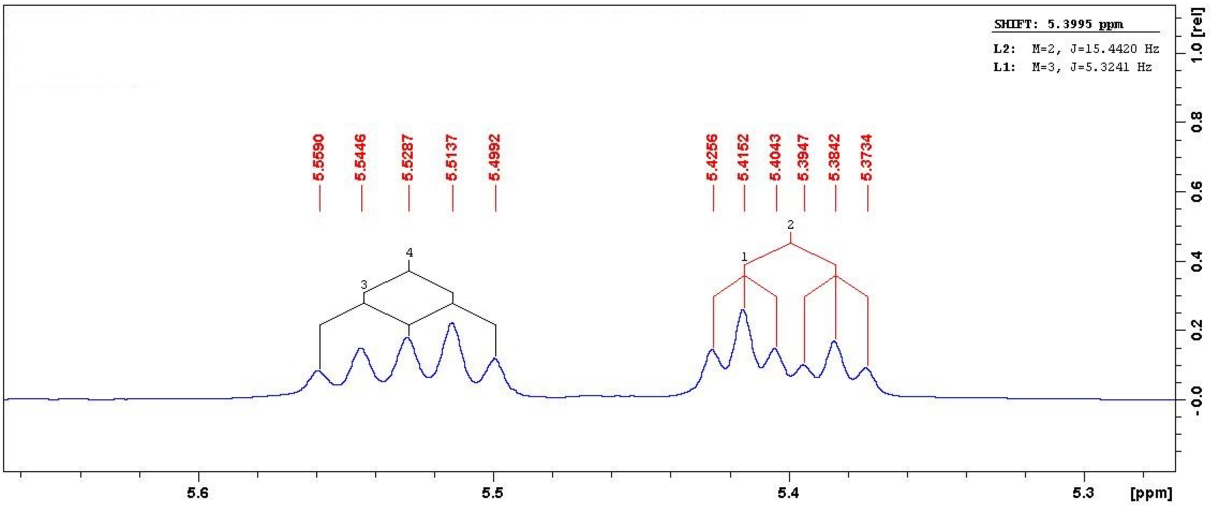

Figure S17. Part of ^{1}H-NMR spectrum of compound **16** in CD_3OD with defined multiplet and coupling constants (J =15.4, 5.3 Hz) that prove the (E)-configuration of double bond.

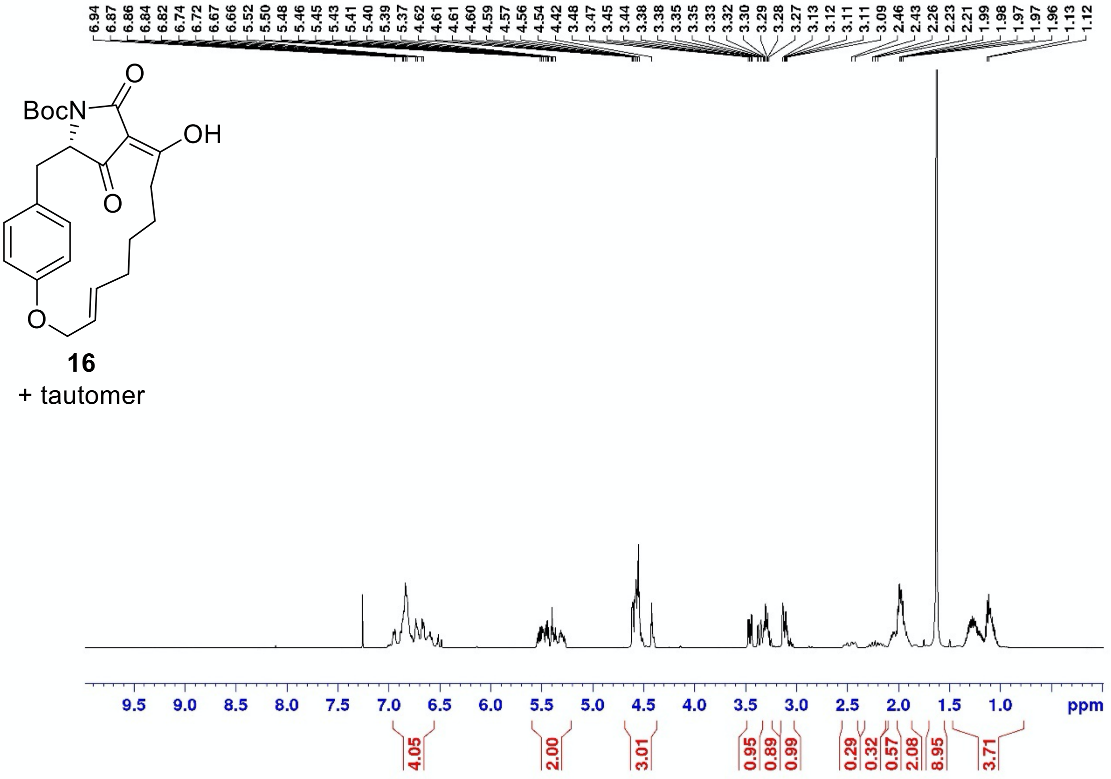

Figure S18. ^{1}H-NMR spectrum of compound **16** in $CDCl_3$.

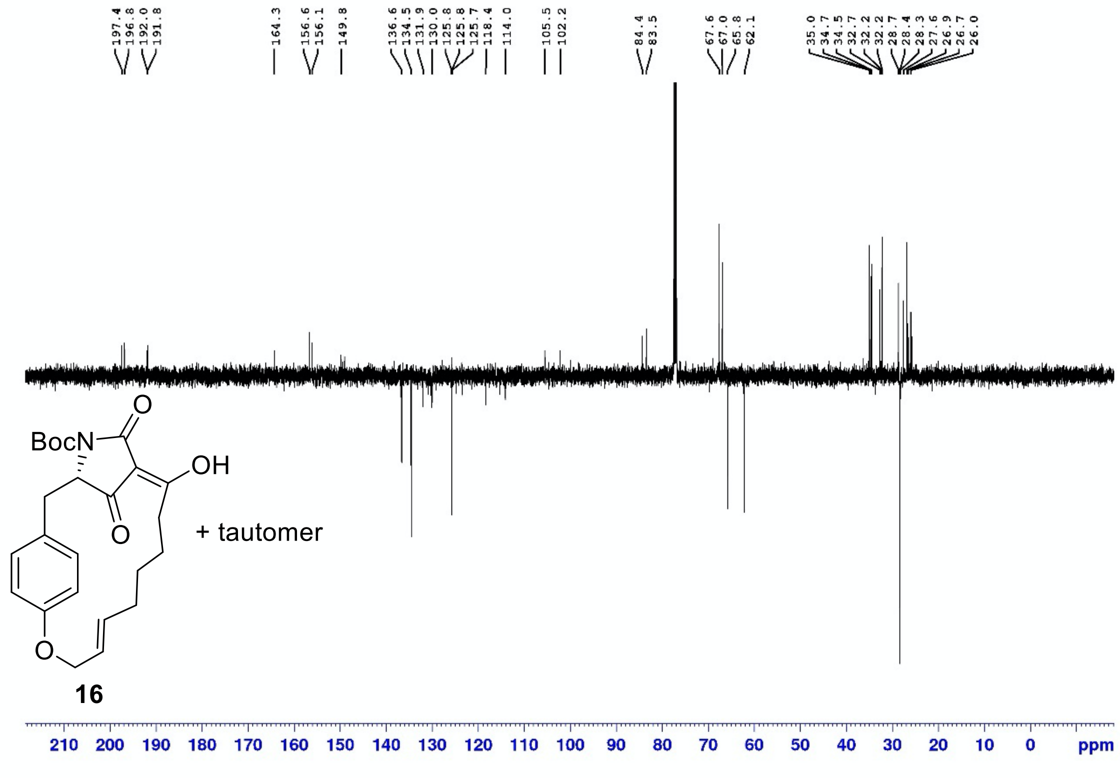

Figure S19. ^{1}H-NMR spectrum of compound **16** in $CDCl_3$.

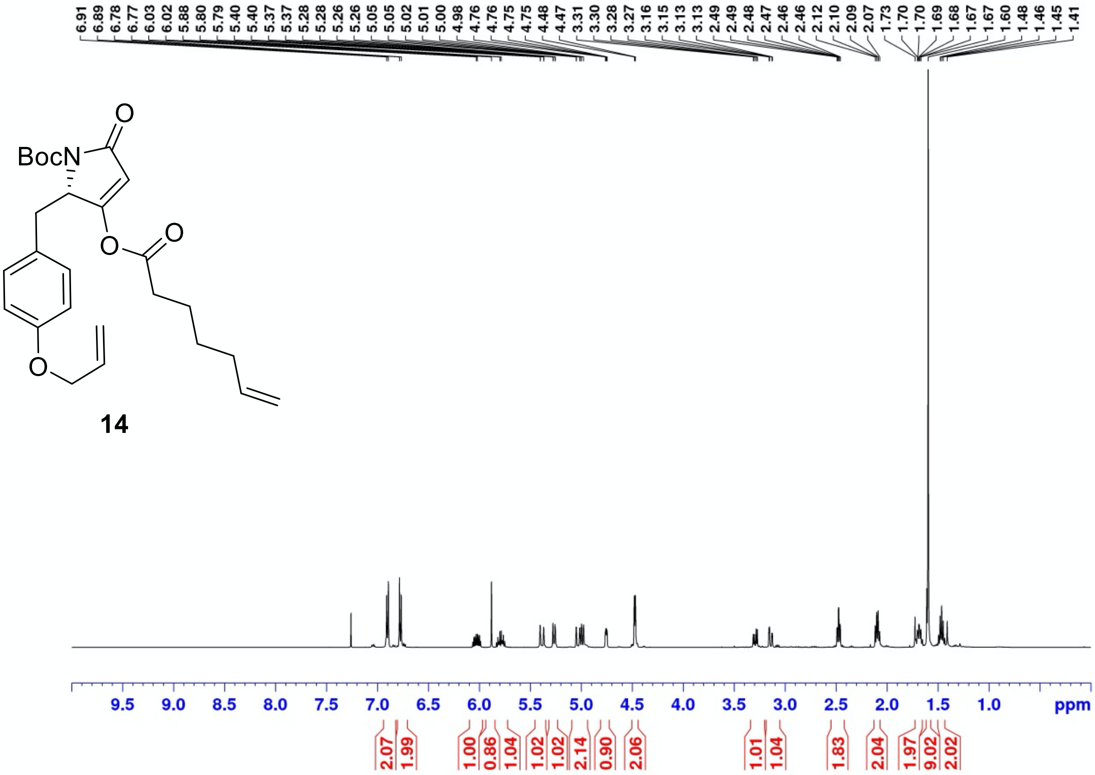

Figure S20. ^{1}H-NMR spectrum of compound **14** in $CDCl_3$.

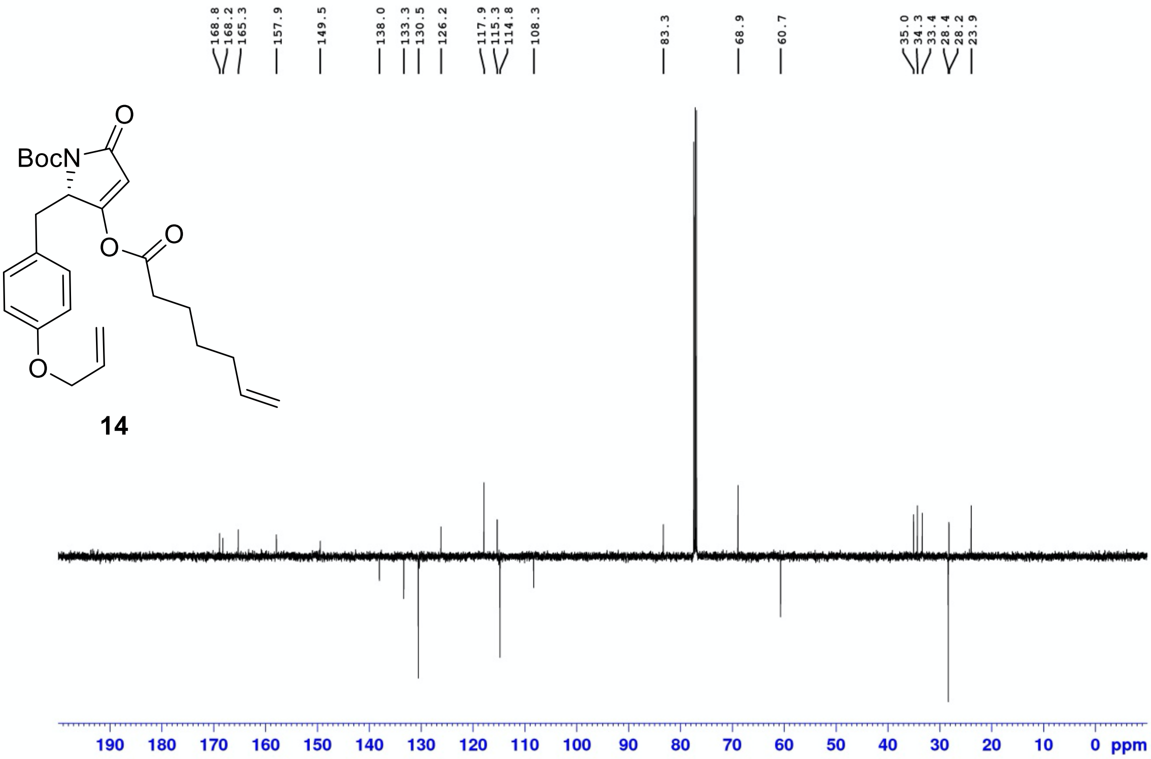

Figure S21. ^{13}C-NMR spectrum of compound **14** in $CDCl_3$.

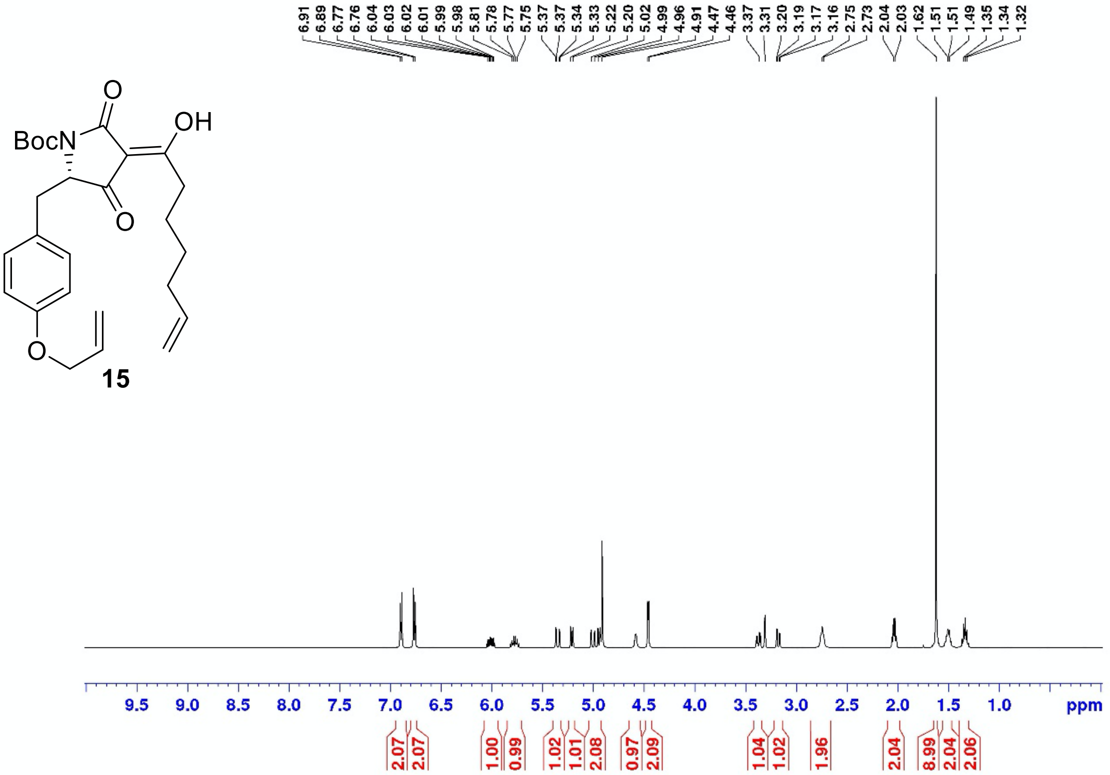

Figure S22. ^{1}H-NMR spectrum of compound **15** in CD_3OD.

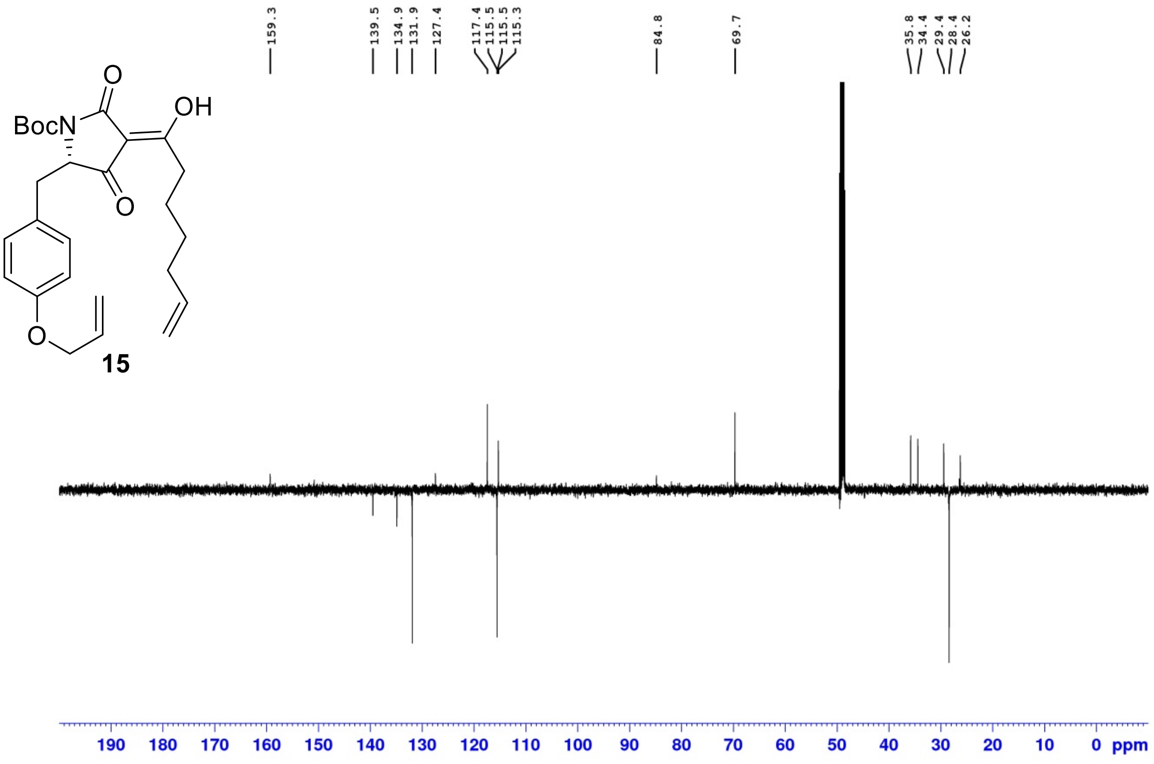

Figure S23. ^{13}C-NMR spectrum of compound **15** in CD_3OD.

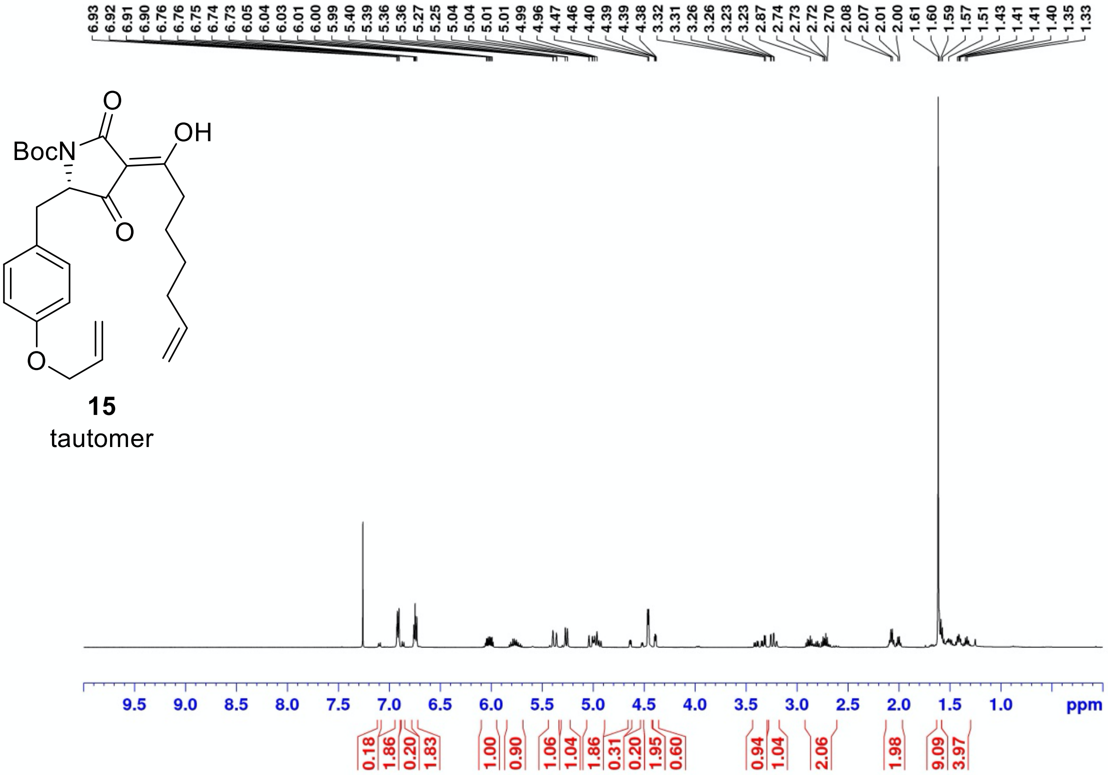

Figure S24. ^{1}H-NMR spectrum of compound **15** in $CDCl_3$.

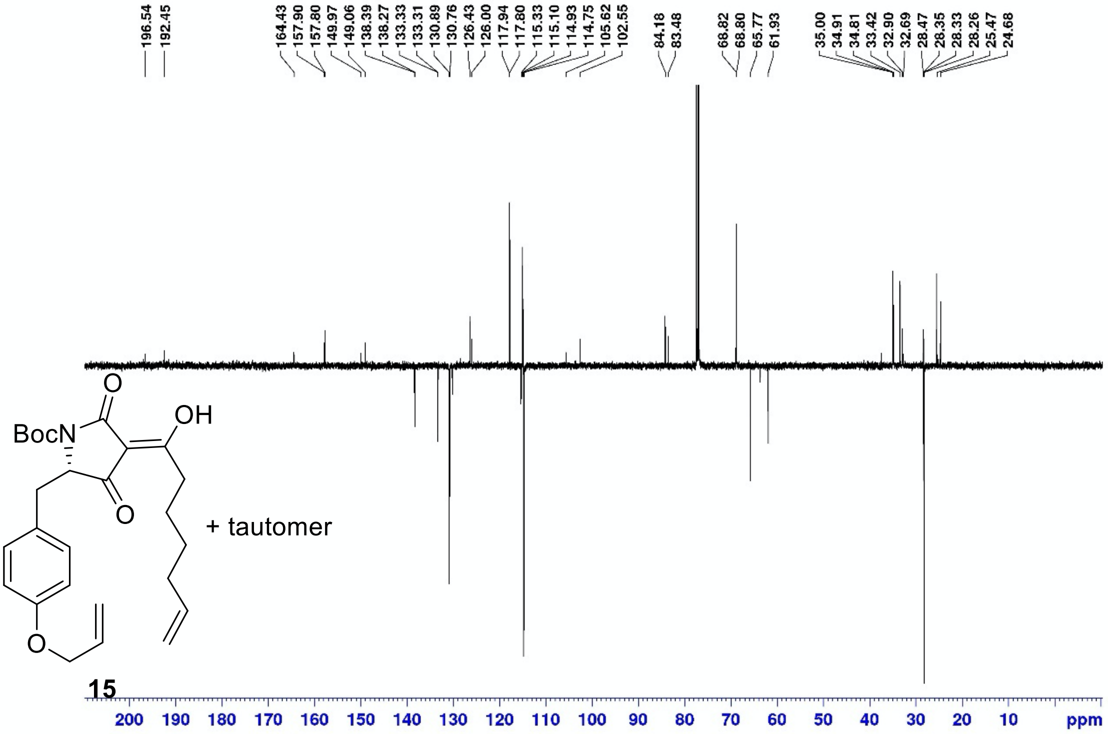

Figure S25. ^{13}C-NMR spectrum of compound **15** in $CDCl_3$.

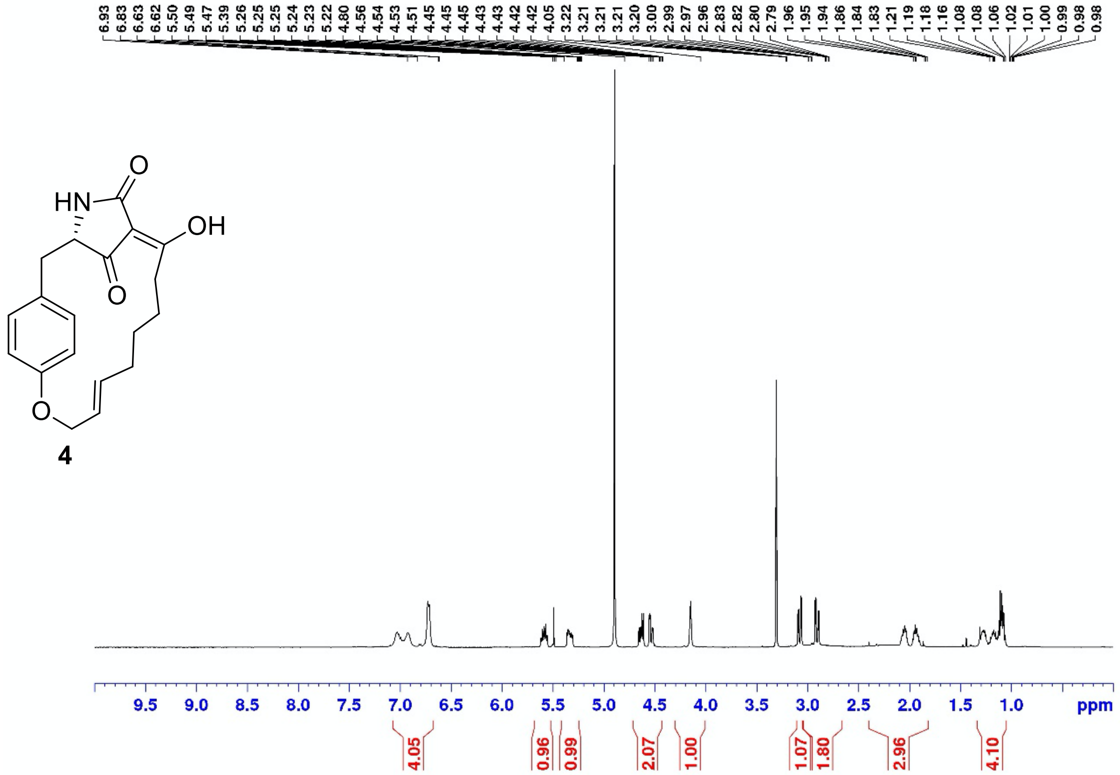

Figure S26. ^{1}H-NMR spectrum of compound **4** in CD_3OD.

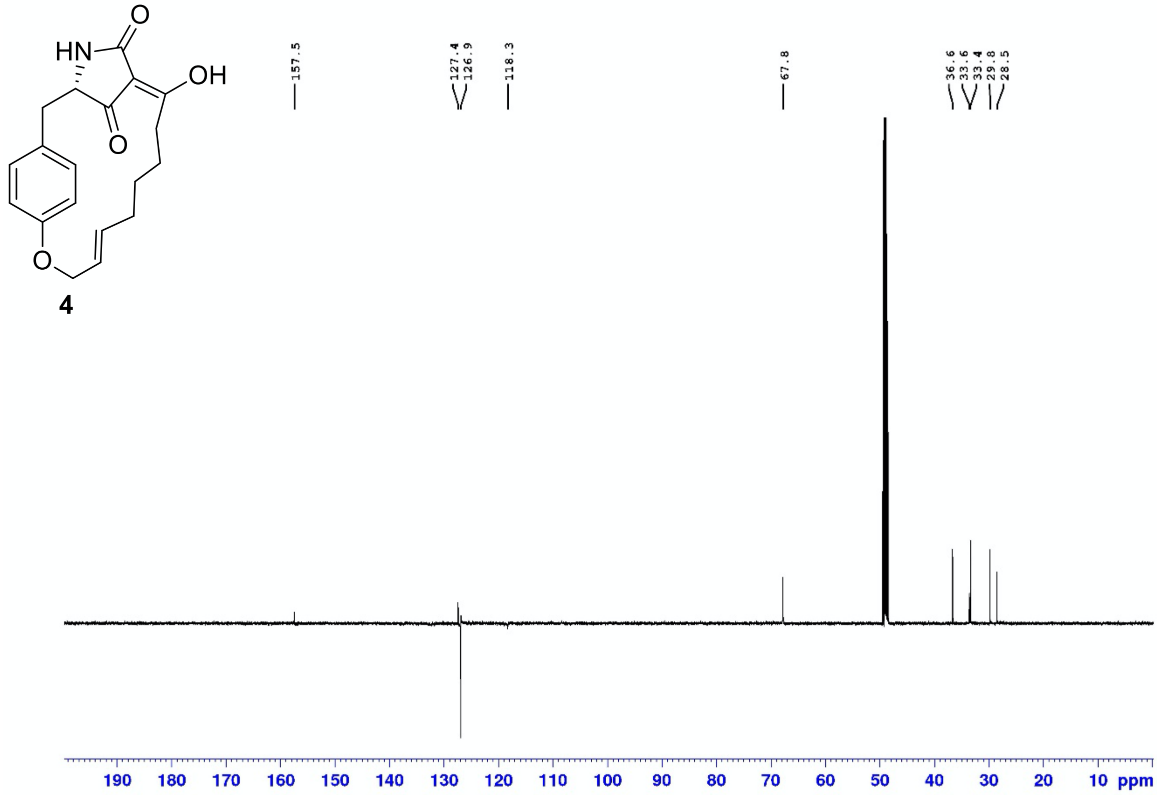

Figure S27. ^{13}C-NMR spectrum of compound **4** in CD_3OD.

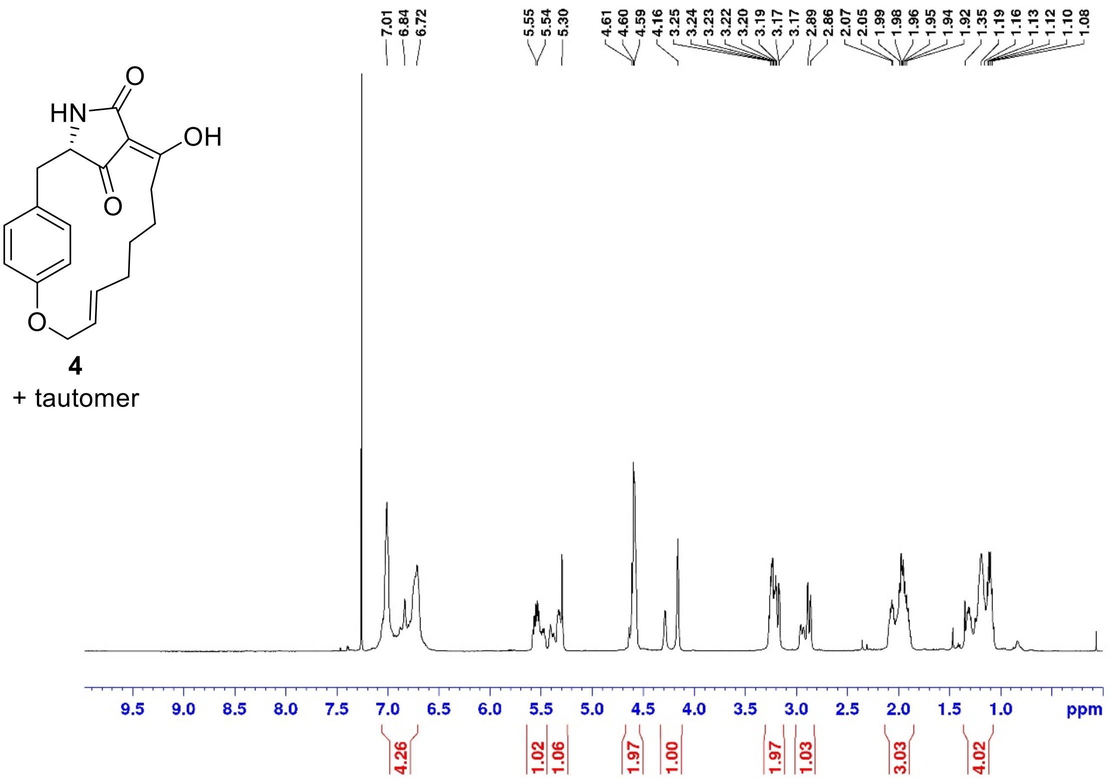

Figure S28. ^{1}H-NMR spectrum of compound **4** in $CDCl_3$.

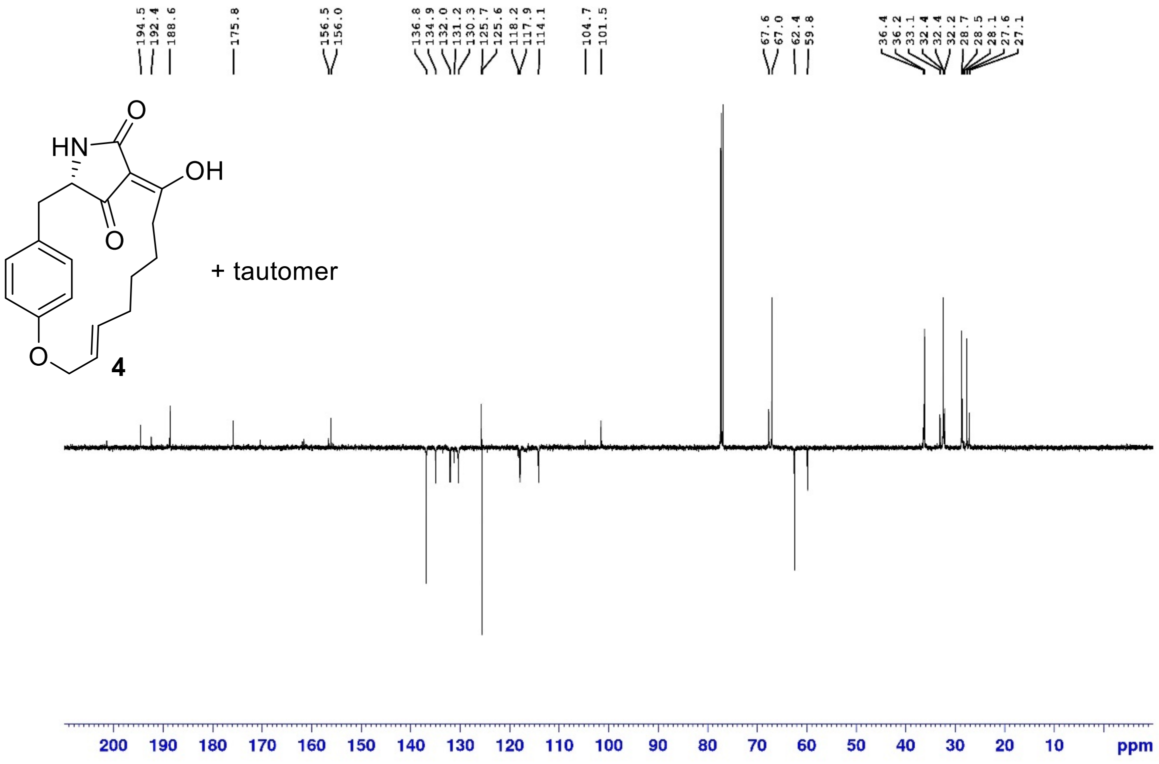

Figure S29. ^{13}C-NMR spectrum of compound **4** in $CDCl_3$.

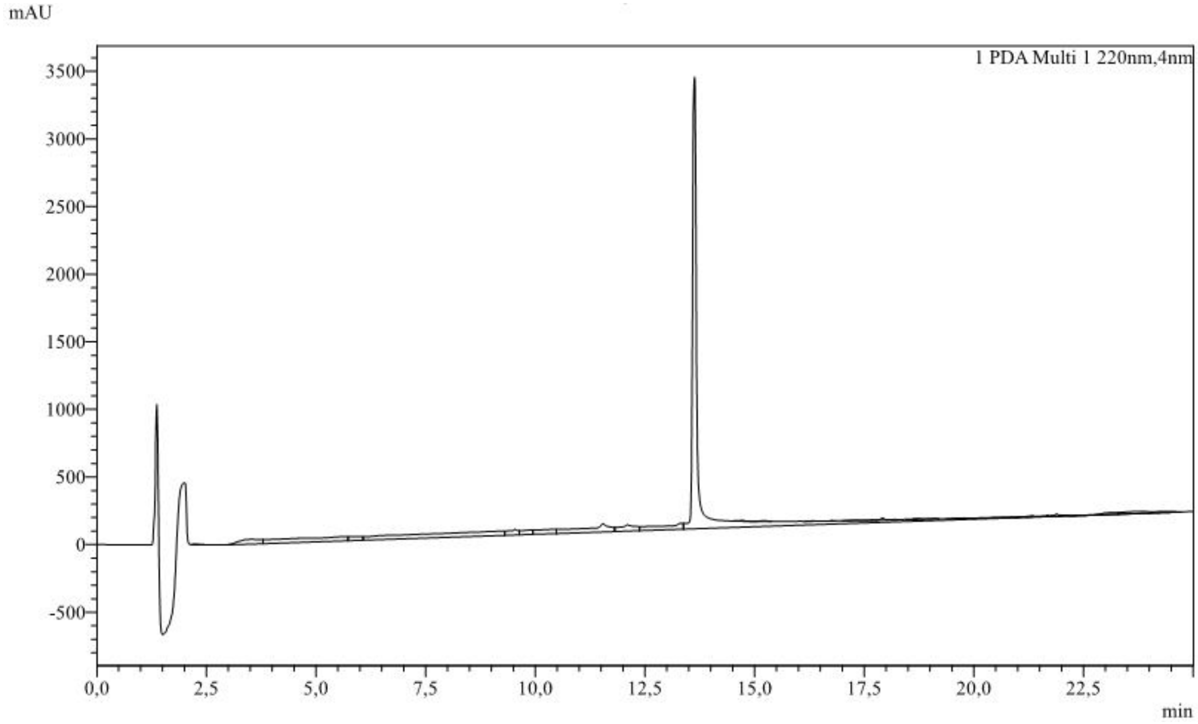

Figure S30. Chromatogram of compound **4**. HPLC: *Shimadzu Nexera XR,* Autosampler *SIL-20A*, diode array detector *SPD-M20A*, C18-column (150 × 4 mm). Method: 10% MeCN in H_2O + 0.1% HCOOH → 97% MeCN in H_2O + 0.1% HCOOH, flow: 1.0 mL/min.

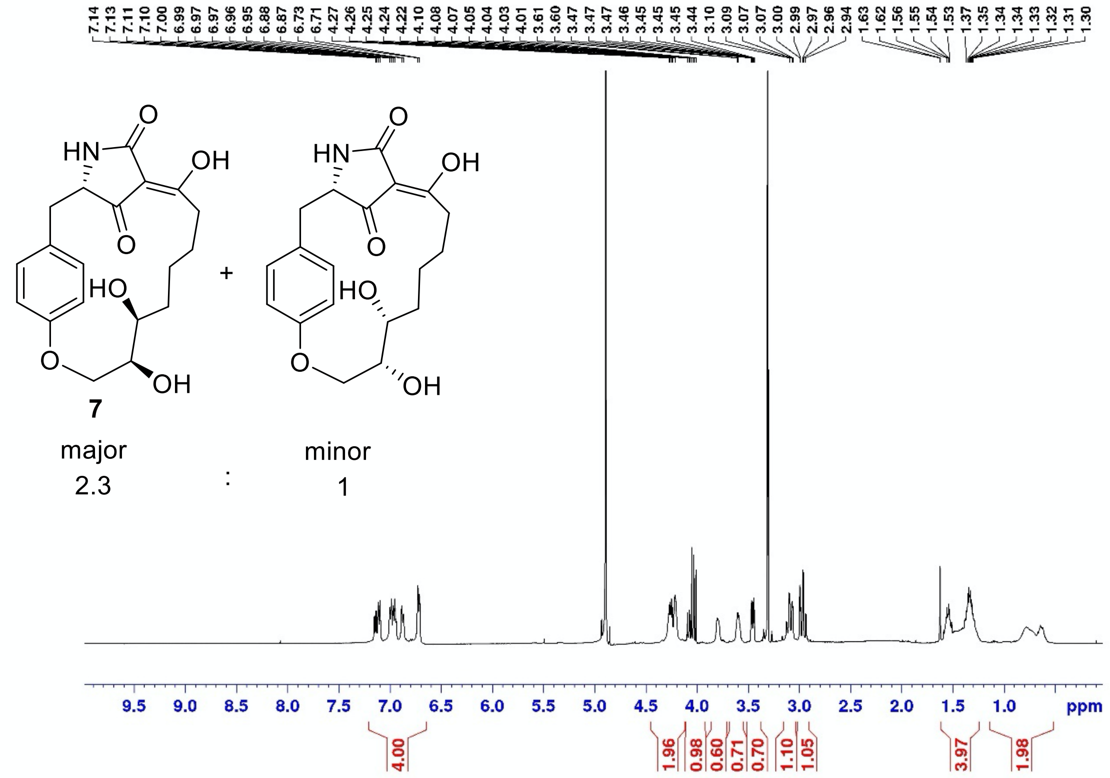

Figure S31. ^{1}H-NMR spectrum of compound **7** in CD_3OD.

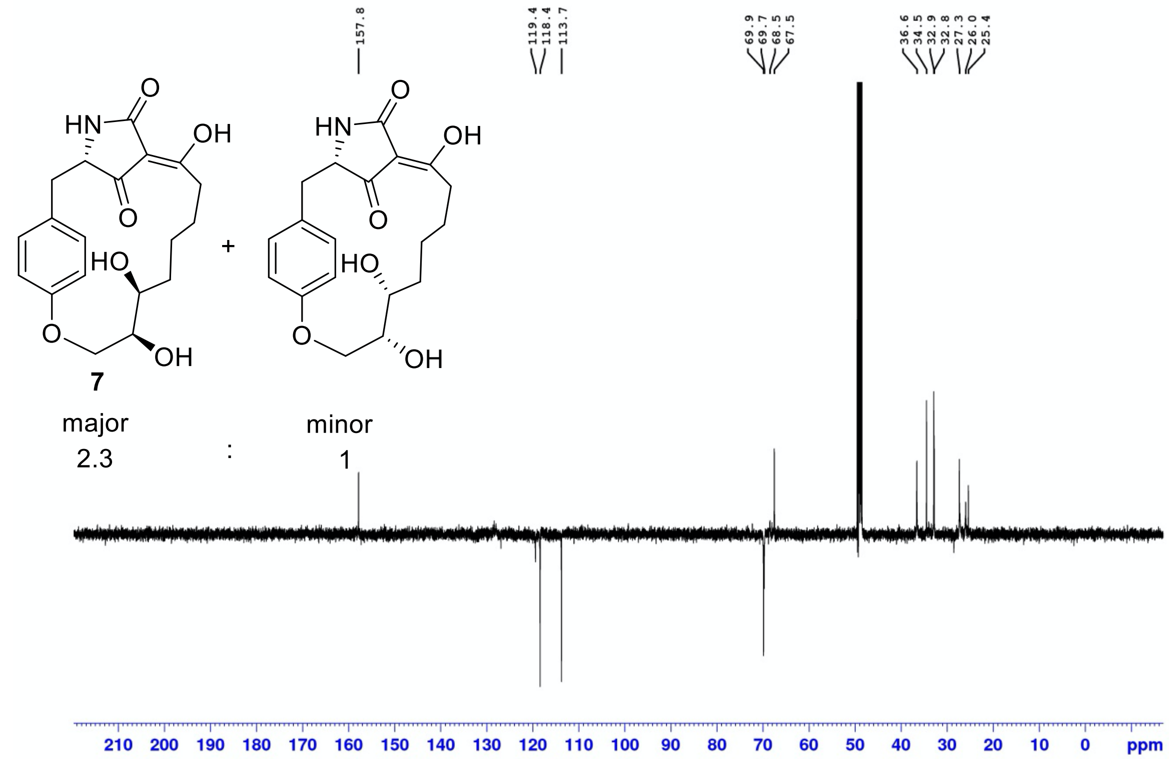

Figure S32. ^{13}C-NMR spectrum of compound **7** in CD_3OD.

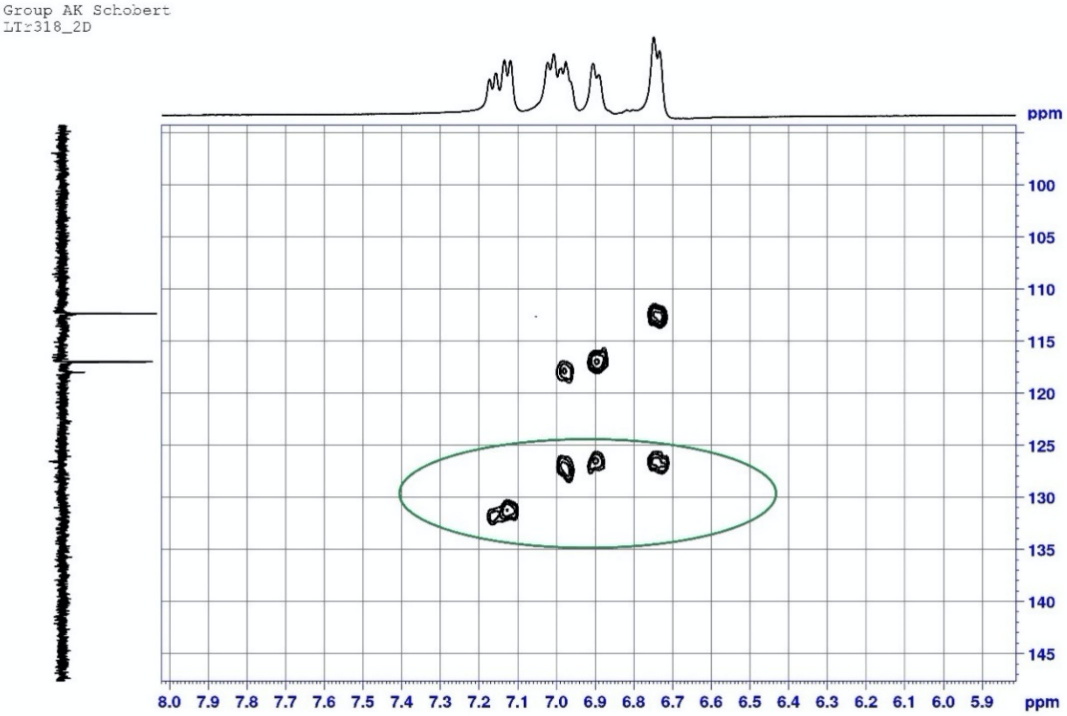

Figure S33. Part of HMBC-2D-NMR spectrum of compound **7** in CD_3OD, which was used for peak assignment.

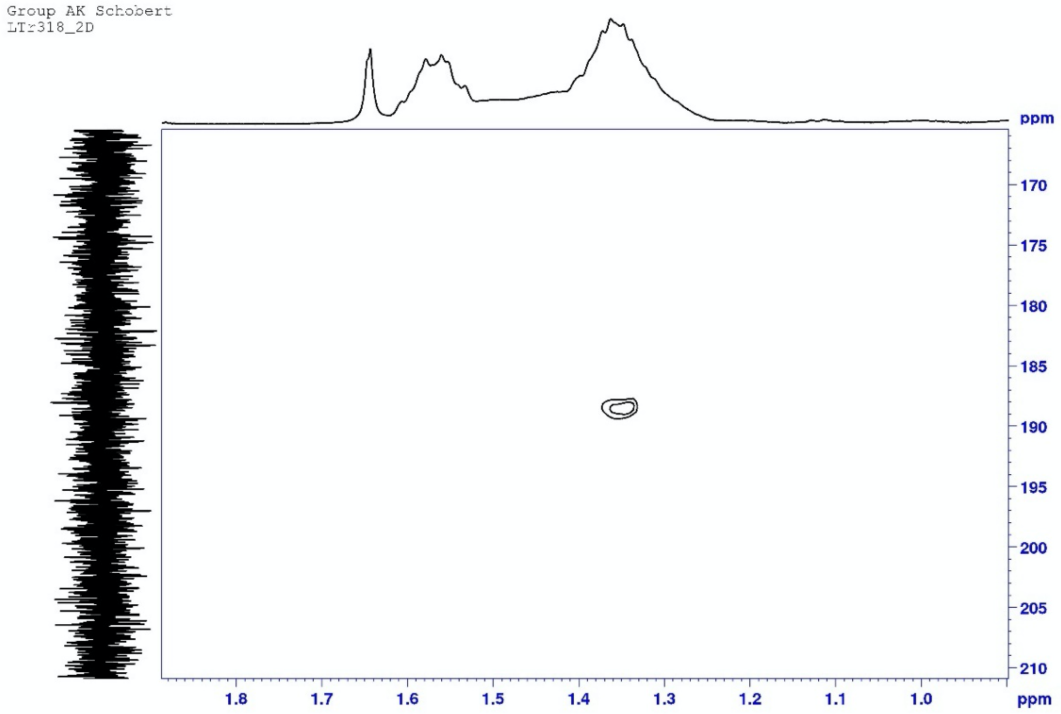

Figure S34. Part of HMBC-2D-NMR spectrum of compound **7** in CD_3OD, which was used for peak assignment.

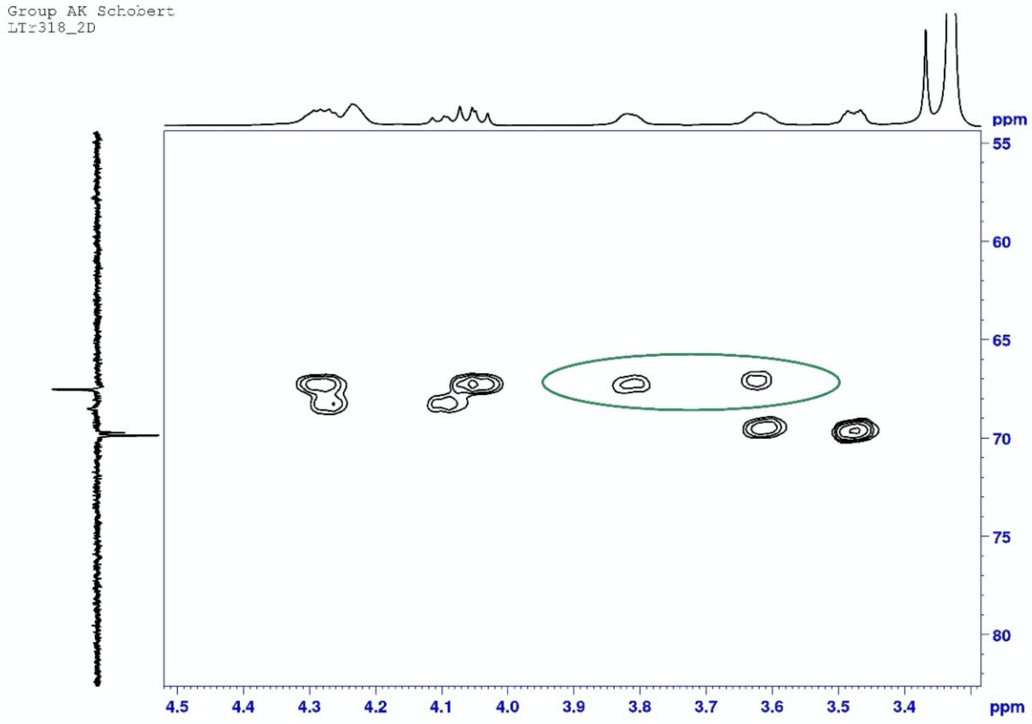

Figure S35. Part of HSQC-2D-NMR spectrum of compound **7** in CD_3OD, which was used for peak assignment.

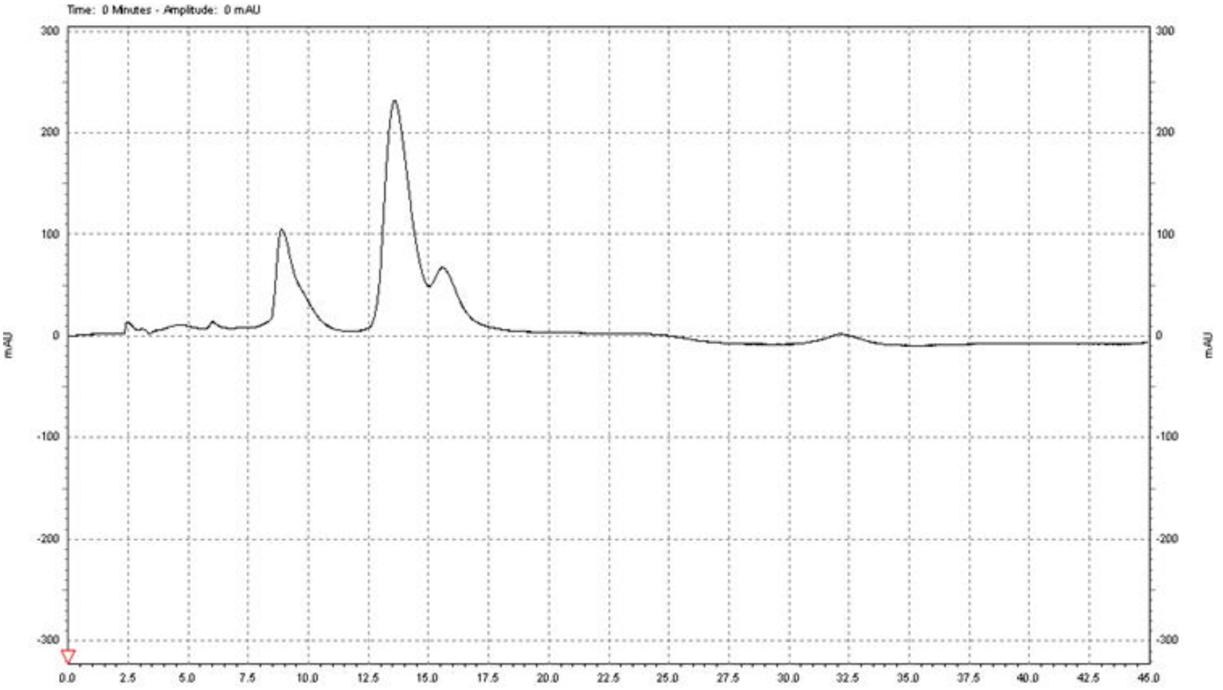

Figure S36. Chromatogram of compound **7**. HPLC: *Beckmann System Gold Programmable Solvent Modul 126,* Beckmann instruments diode array detection module 128, Phenomenex Lux® Amylose-1-HPLC column (100 × 4.6 mm). Method: 30% EtOH in hexanes → 40% EtOH in hexanes → 50% EtOH in hexanes → 60% EtOH in hexanes → 80% EtOH in hexanes, flow: 0.7 mL/min. A mixture of tautomers and diastereomers can be seen

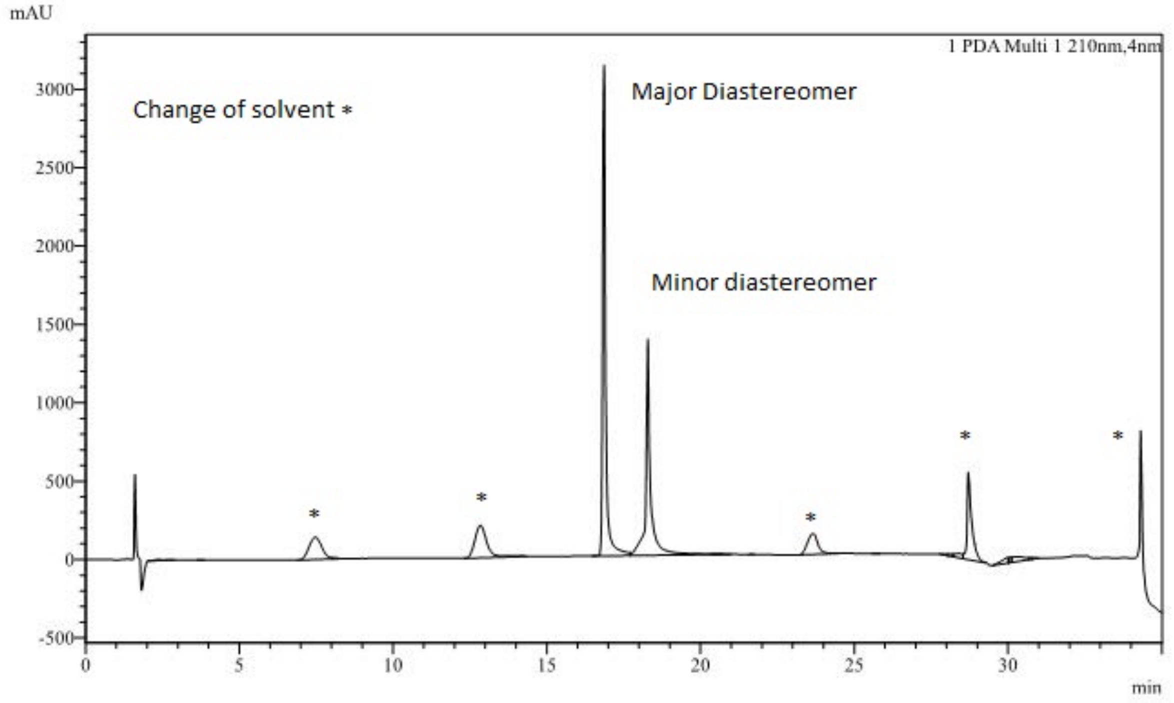

Figure S37. Chromatogram of compound **7**. HPLC: *Shimadzu Nexera XR,* Autosampler *SIL-20A*, diode array detector *SPD-M20A*, C18-column (150 × 4 mm). Method: 5% MeCN in H_2O + 0.1% HCOOH → 10% MeCN in H_2O + 0.1% HCOOH → 20% MeCN in H_2O + 0.1% HCOOH → 30% MeCN in H_2O + 0.1% HCOOH → 40% MeCN in H_2O + 0.1% HCOOH→ 97% MeCN in H_2O + 0.1% HCOOH, flow: 1.0 mL/min.

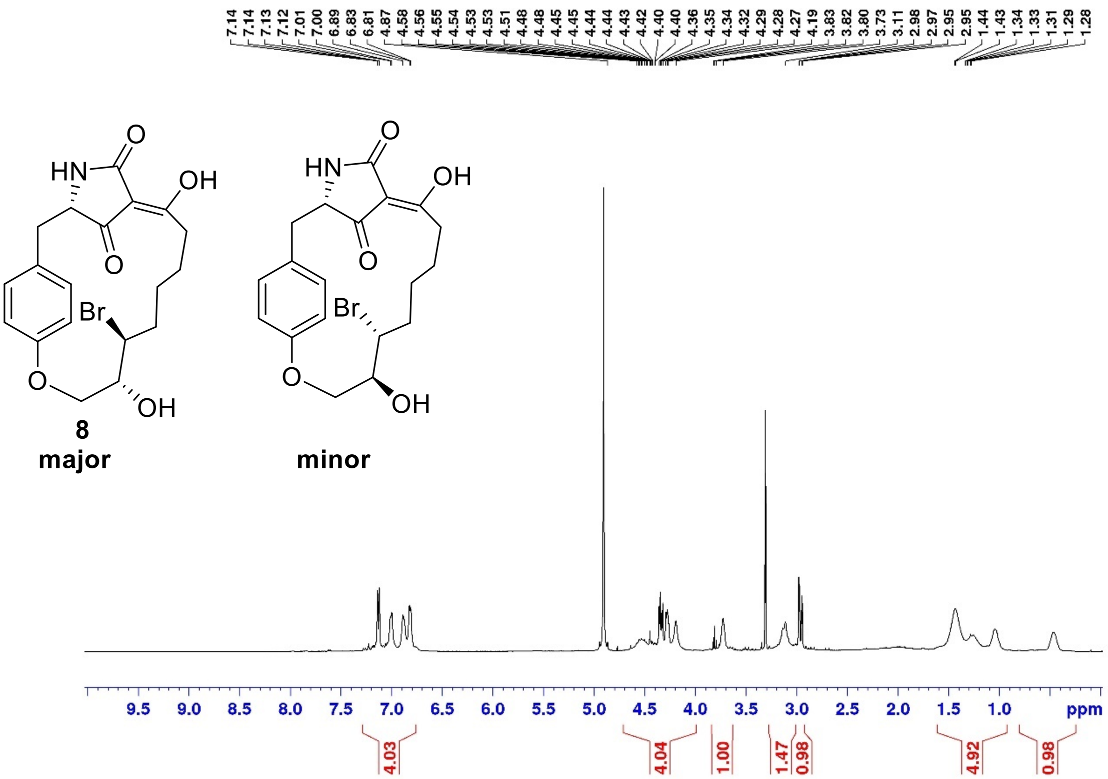

Figure S38. ^{1}H-NMR spectrum of compound **8** in CD_3OD.

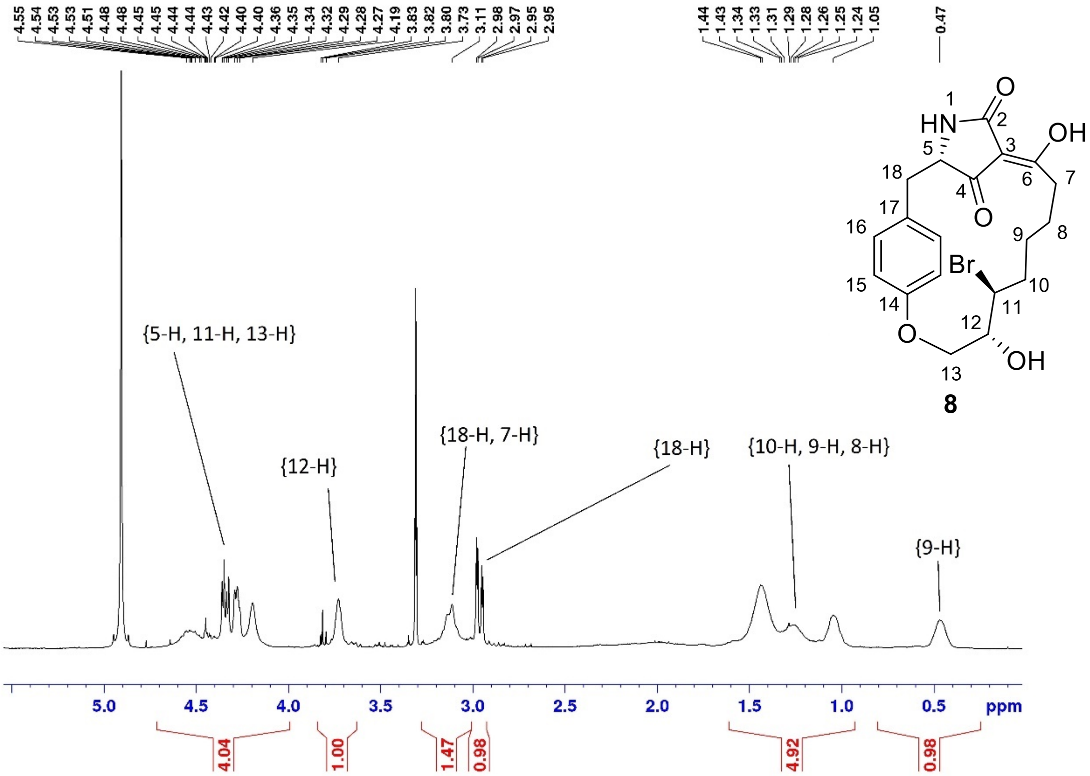

Figure S39. ^{1}H-NMR spectrum of compound **8** between 5.5 ppm and 0.0 ppm in CD_3OD with assignment of signals.

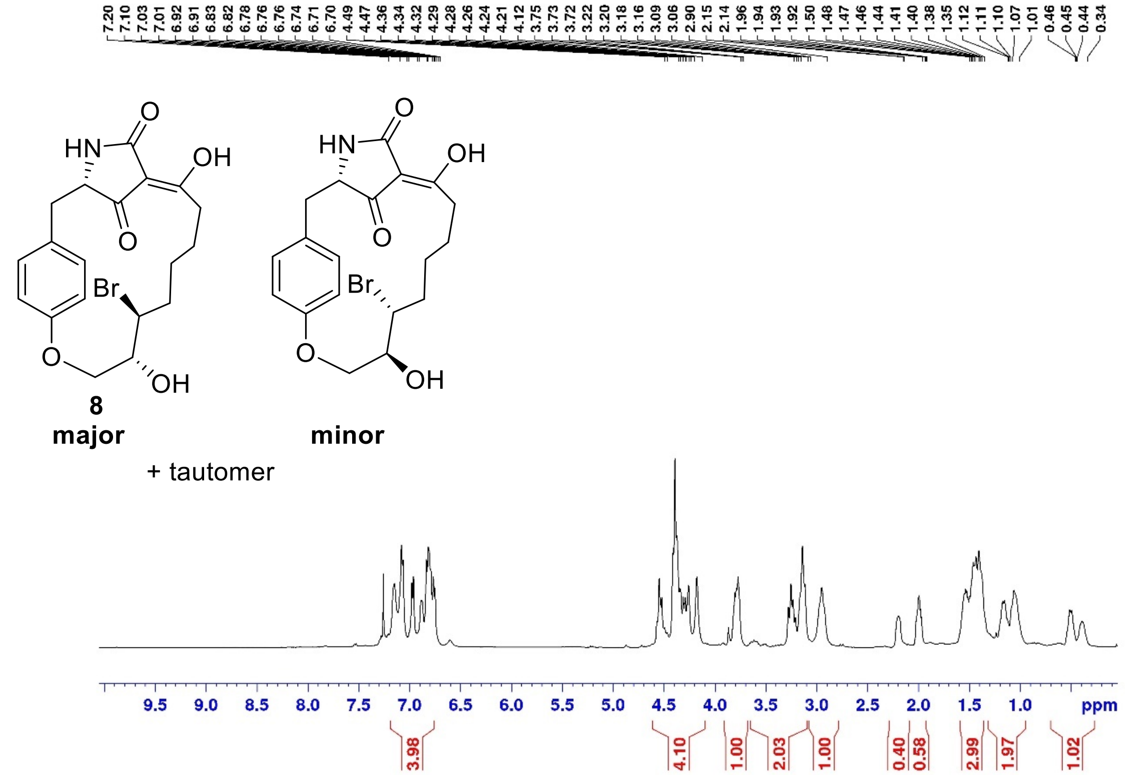

Figure S40. ^{1}H-NMR spectrum of compound **8** in $CDCl_3$.

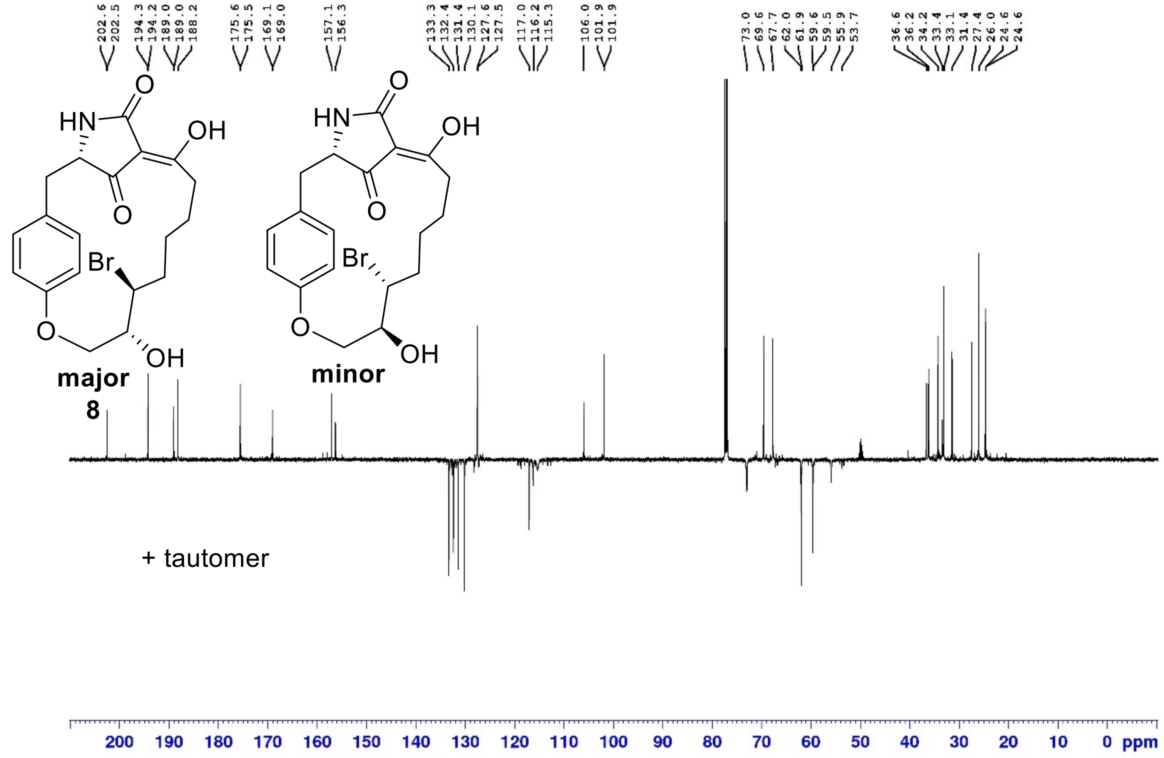

Figure S41. ^{13}C-NMR spectrum of compound **8** in $CDCl_3$.

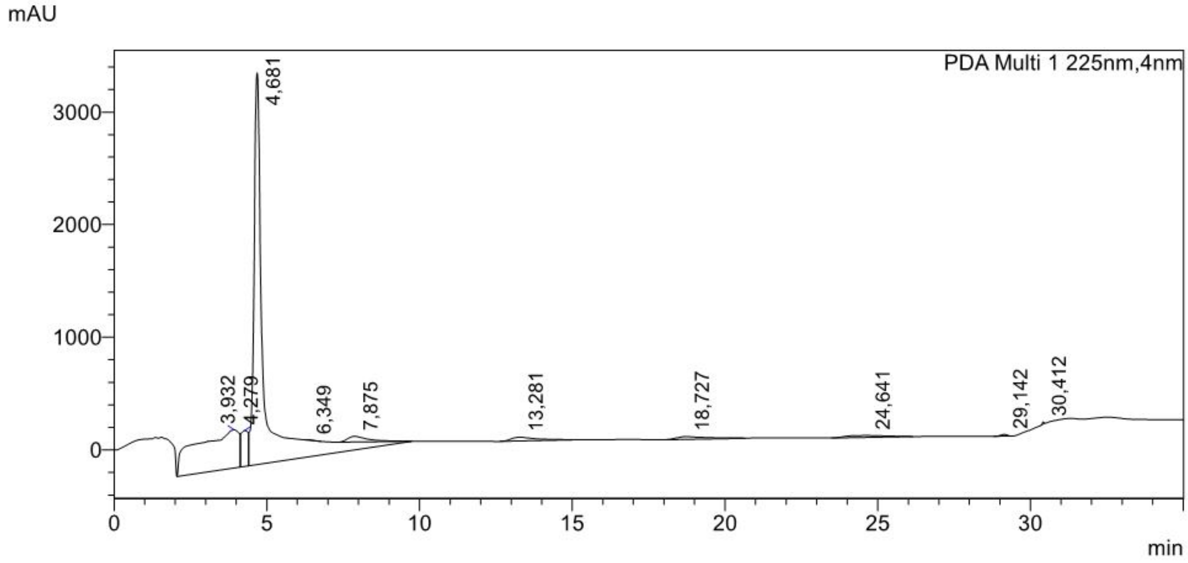

Figure S42. Chromatogram of compound **8**. HPLC: *Shimadzu Nexera XR,* Autosampler *SIL-20A*, diode array detector *SPD-M20A*, C18-column (150 × 4 mm). Method: 30% MeCN in H_2O + 0.1% HCOOH → 35% MeCN in H_2O + 0.1% HCOOH → 40% MeCN in H_2O + 0.1% HCOOH → 45% MeCN in H_2O + 0.1% HCOOH → 50% MeCN in H_2O + 0.1% HCOOH→ 97% MeCN in H_2O + 0.1% HCOOH, flow: 1.0 mL/min.

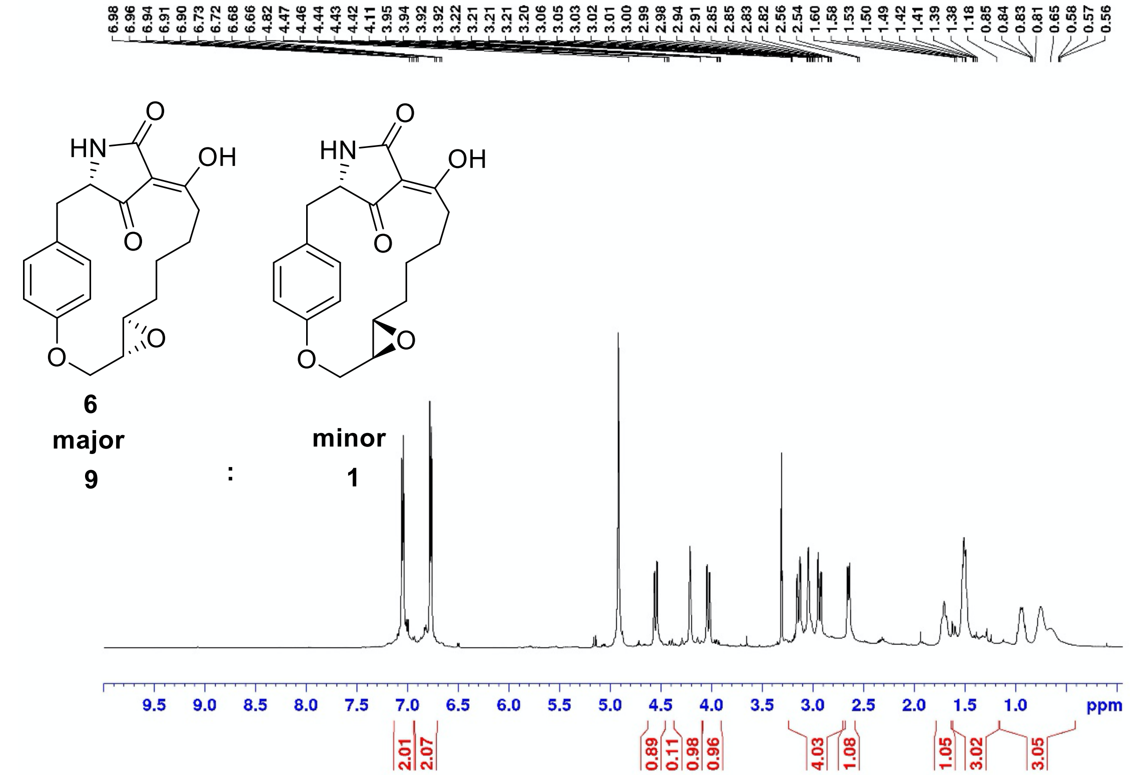

Figure S43. ^{1}H-NMR spectrum of compound **6** in CD_3OD.

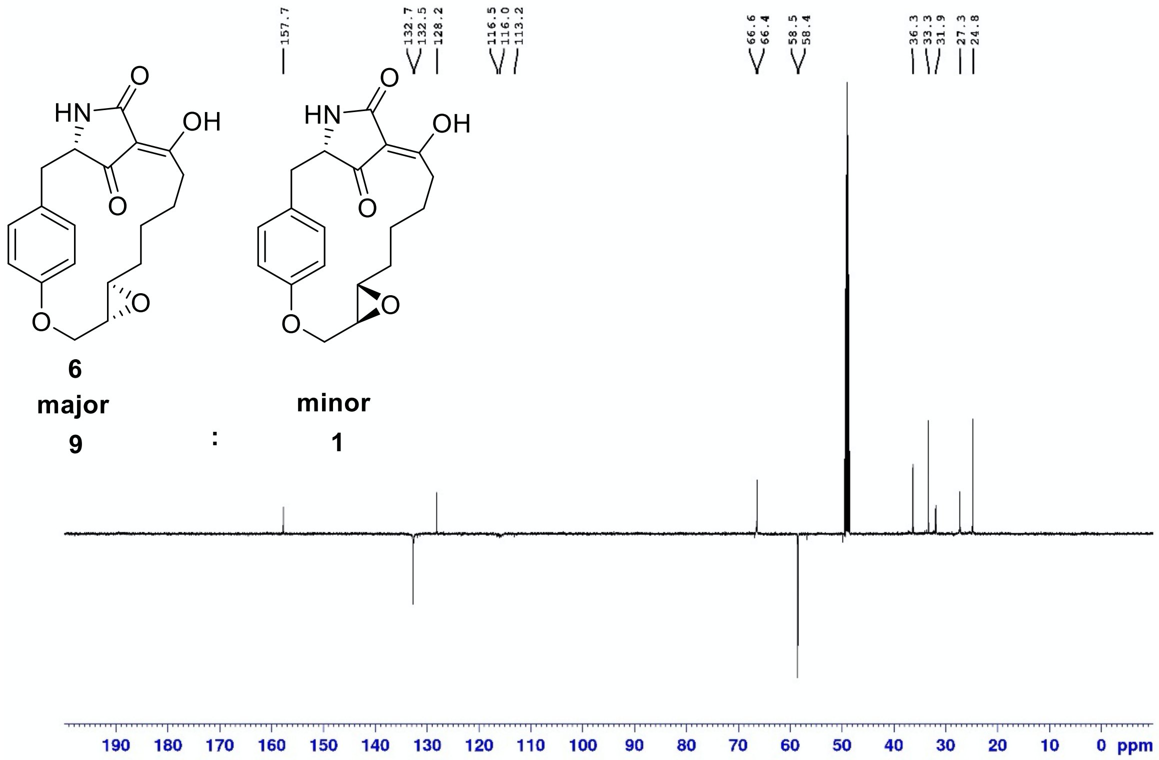

Figure S44. ^{13}C-NMR spectrum of compound **6** in CD_3OD.

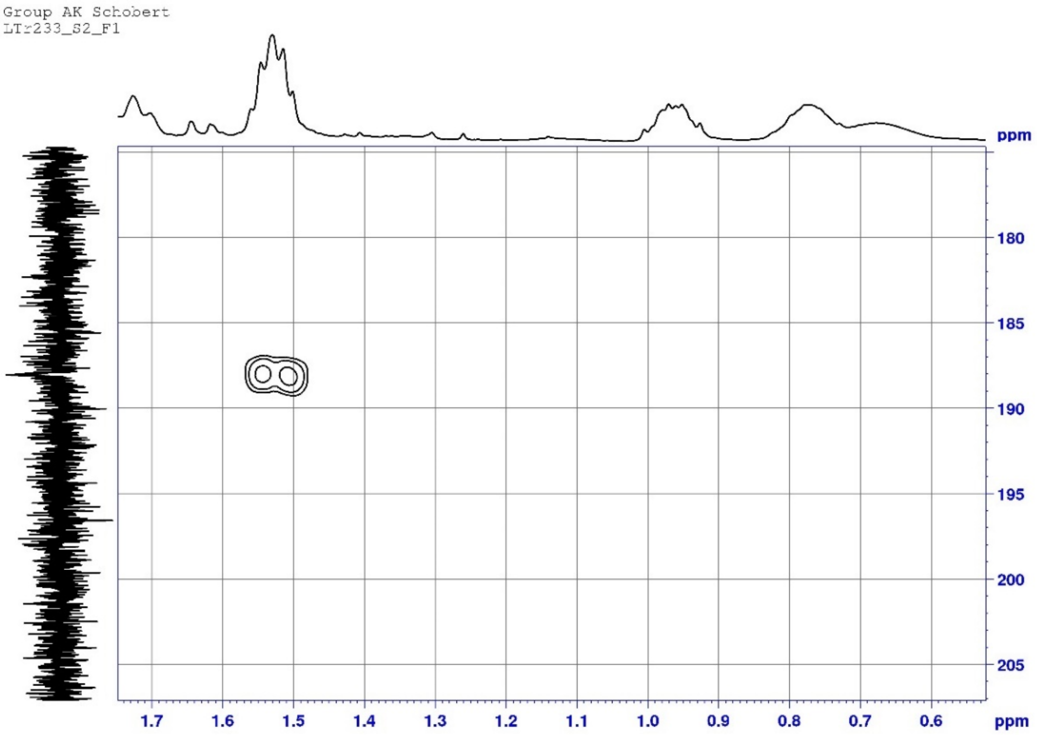

Figure S45. Part of HMBC-2D-NMR spectrum of compound **6** in CD_3OD, which was used for peak assignment.

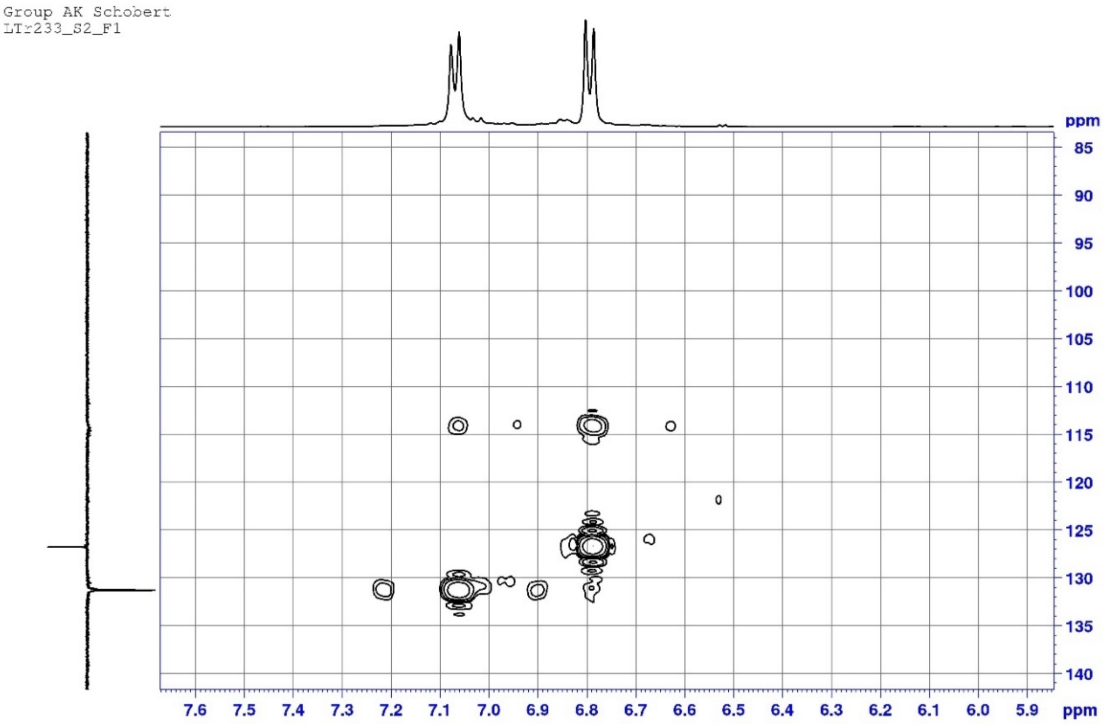

Figure S46. Part of HMBC-2D-NMR spectrum of compound **6** in CD_3OD, which was used for peak assignment.

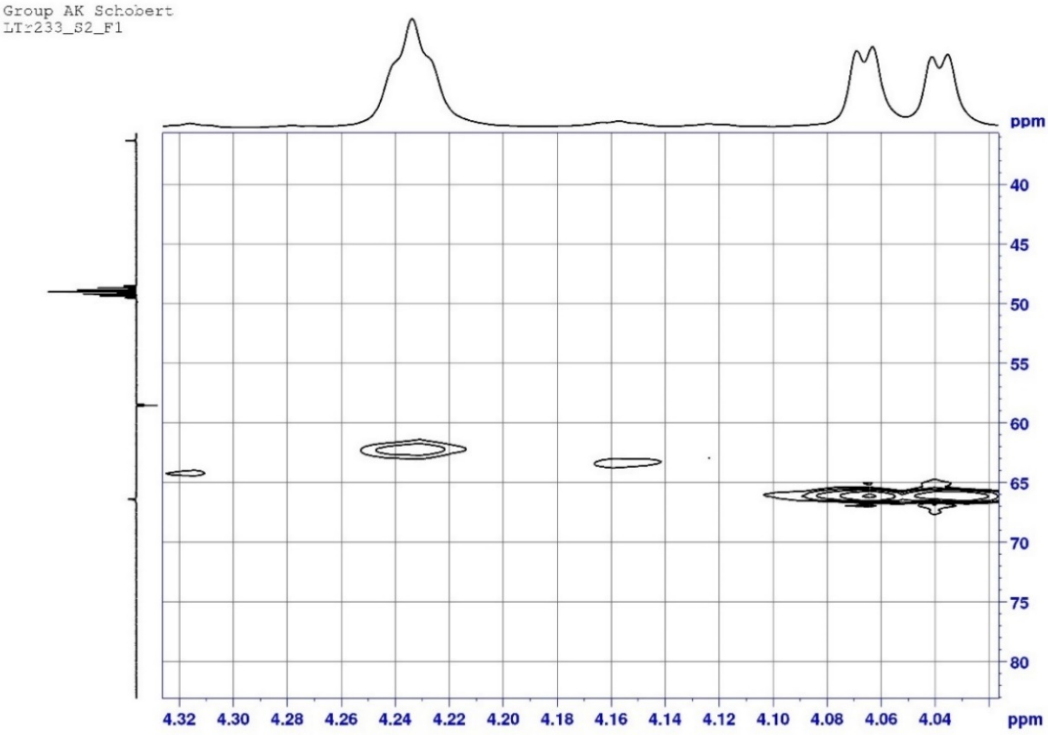

Figure S47. Part of HSQC-2D-NMR spectrum of compound **6** in CD_3OD, which was used for peak assignment.

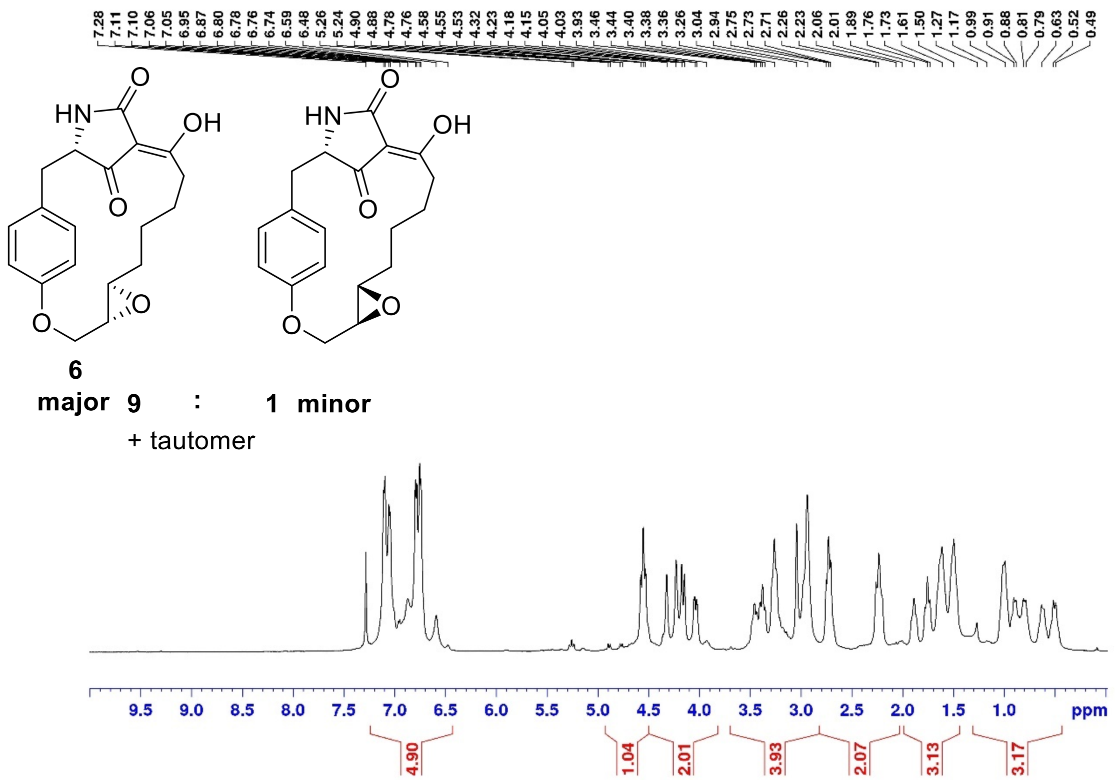

Figure S48. ^{1}H-NMR spectrum of compound **6** in $CDCl_3$.

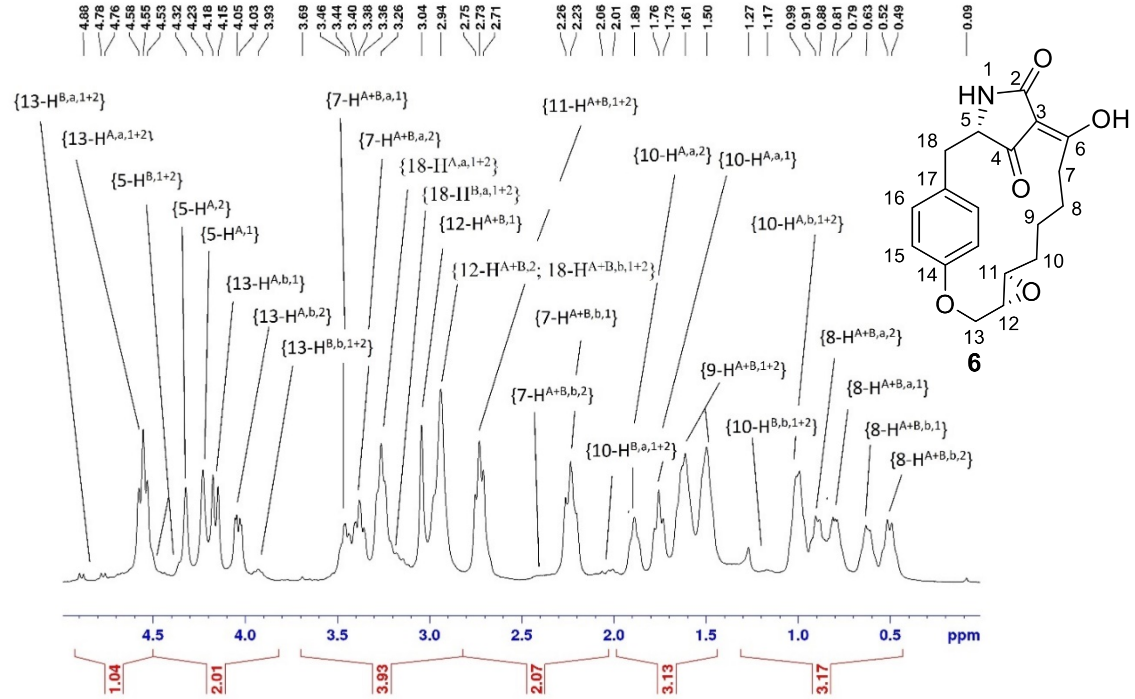

Figure S49. ^{1}H-NMR spectrum of compound **6** between 5.0 ppm and 0.0 ppm in $CDCl_3$ with assignment of signals. Signals of major diastereomer marked as A, signals of minor diastereomer marked as B; diastereotopic H-atoms indicated as a, b; signals of major tautomer marked as 1, signals of minor tautomer marked as 2.

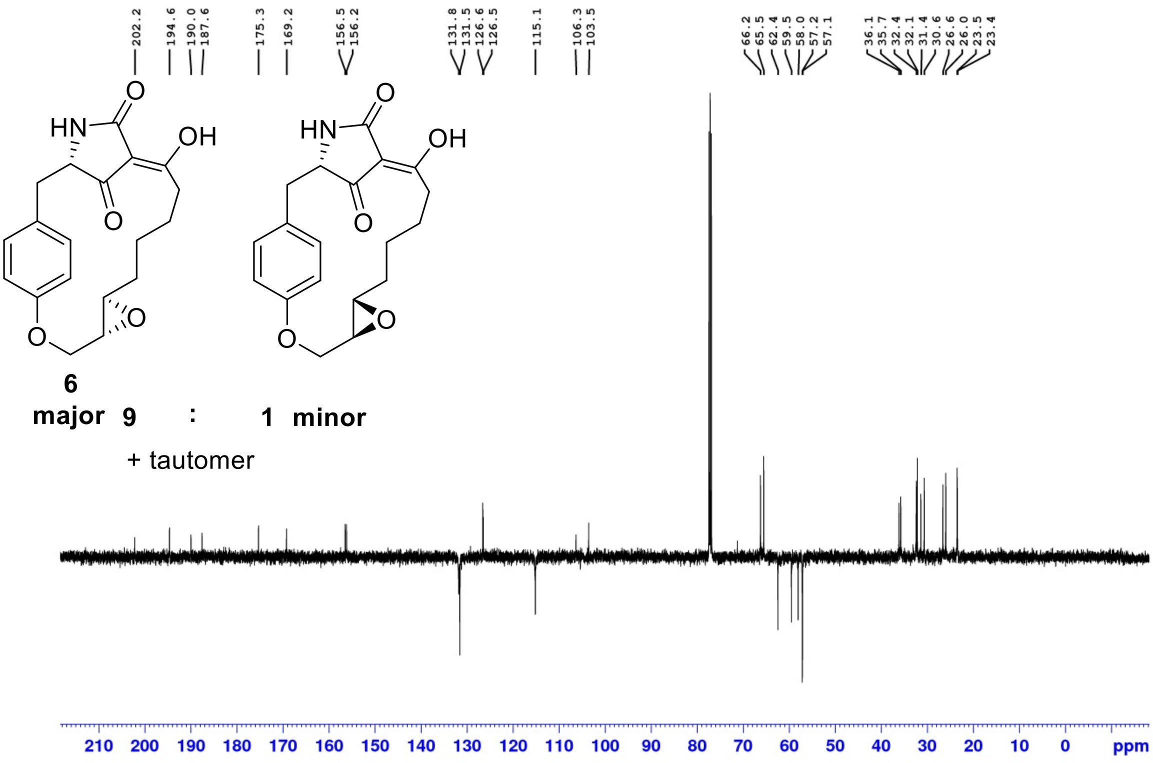

Figure S50. ^{13}C-NMR spectrum of compound **6** in $CDCl_3$.

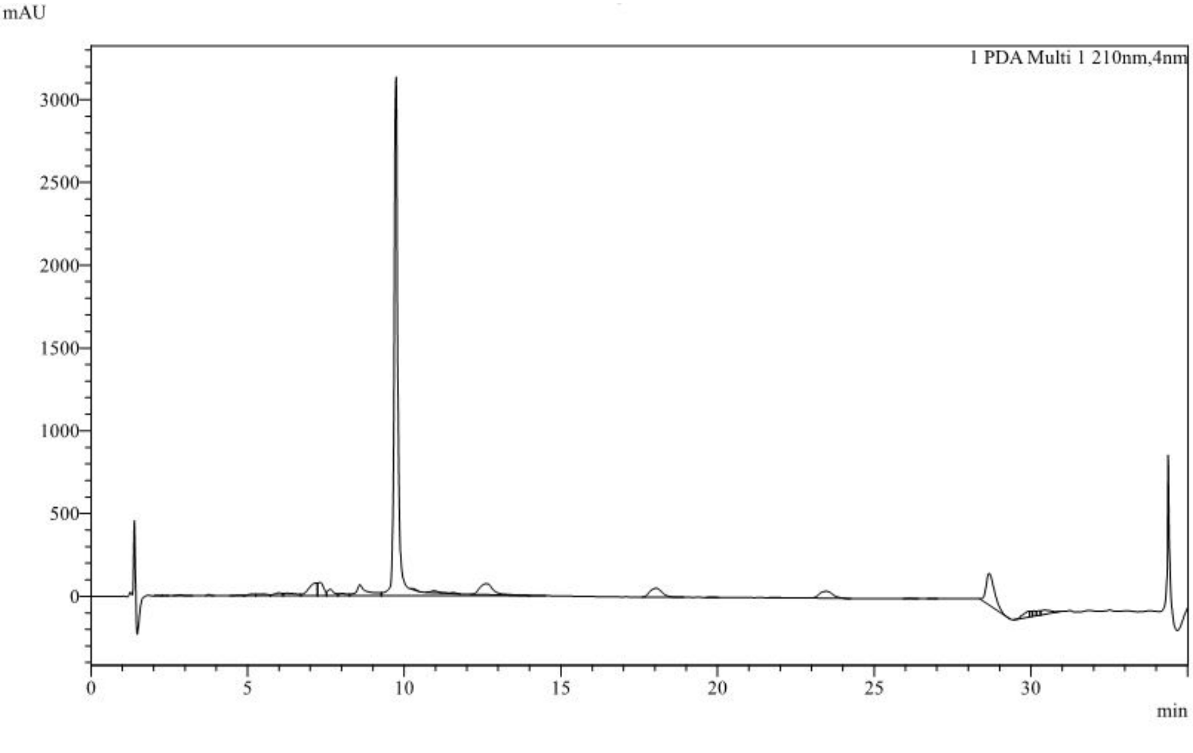

Figure S51. Chromatogram of compound **6**. HPLC: *Shimadzu Nexera XR,* Autosampler *SIL-20A*, diode array detector *SPD-M20A*, C18-column (150 × 4 mm). Method: 30% MeCN in H_2O + 0.1% HCOOH → 35% MeCN in H_2O + 0.1% HCOOH → 40% MeCN in H_2O + 0.1% HCOOH → 45% MeCN in H_2O + 0.1% HCOOH → 50% MeCN in H_2O + 0.1% HCOOH→ 97% MeCN in H_2O + 0.1% HCOOH, flow: 1.0 mL/min.

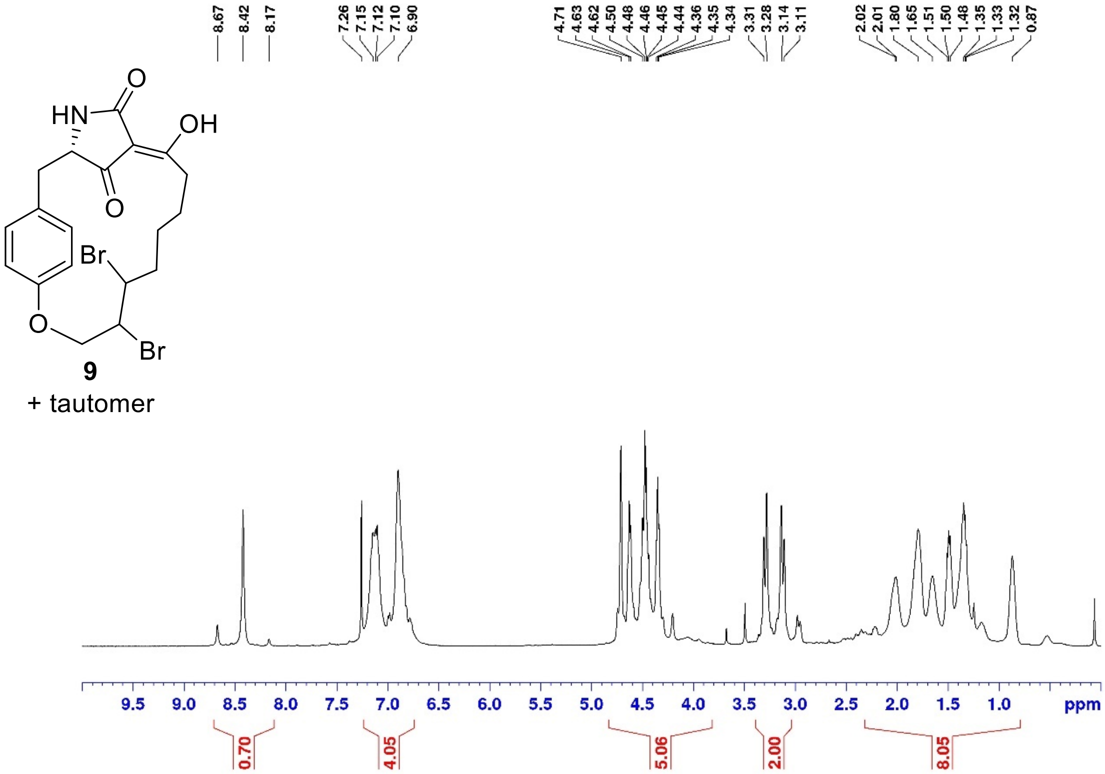

Figure S52. ^{1}H-NMR spectrum of compound **9** in $CDCl_3$.

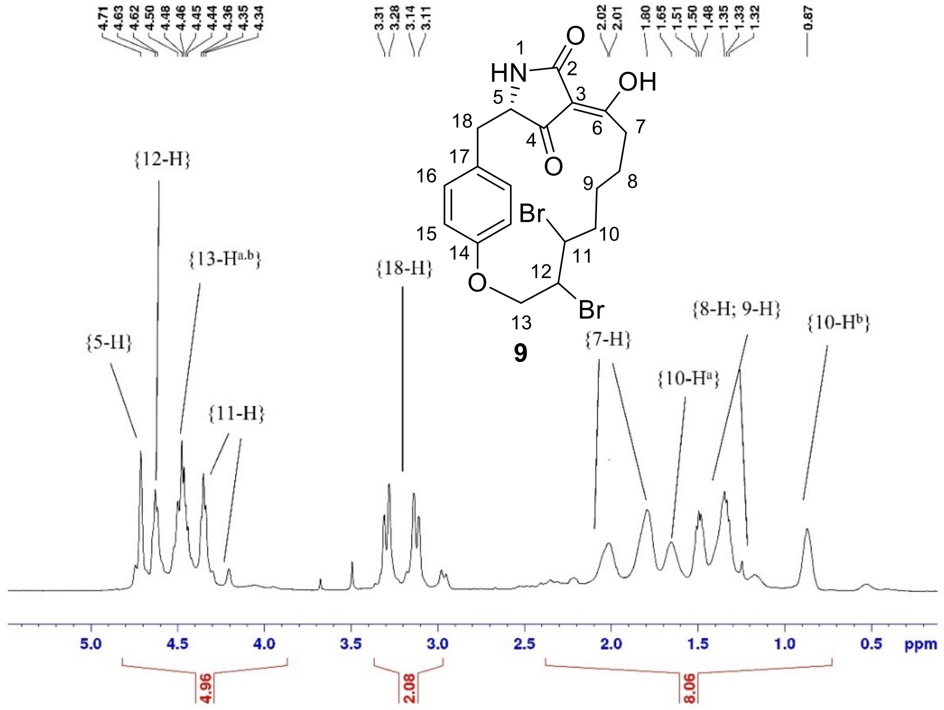

Figure S53. ^{1}H-NMR spectrum of compound **9** between 5.0 ppm and 0.0 ppm in $CDCl_3$ with assignment of signals. Signals of major diastereomer marked as A, signals of minor diastereomer marked as B; diastereotopic H-atoms indicated as a, b; signals of major tautomer marked as 1, signals of minor tautomer marked as 2.

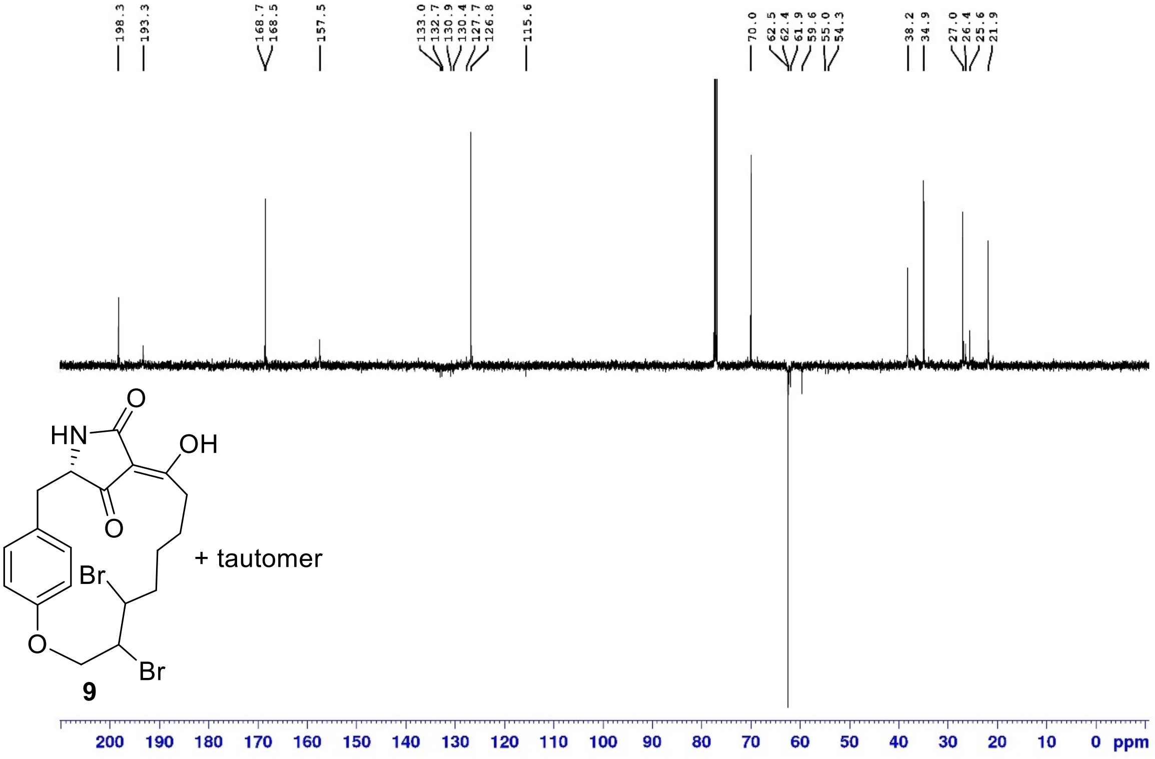

Figure S54. ^{13}C-NMR spectrum of compound **9** in $CDCl_3$.

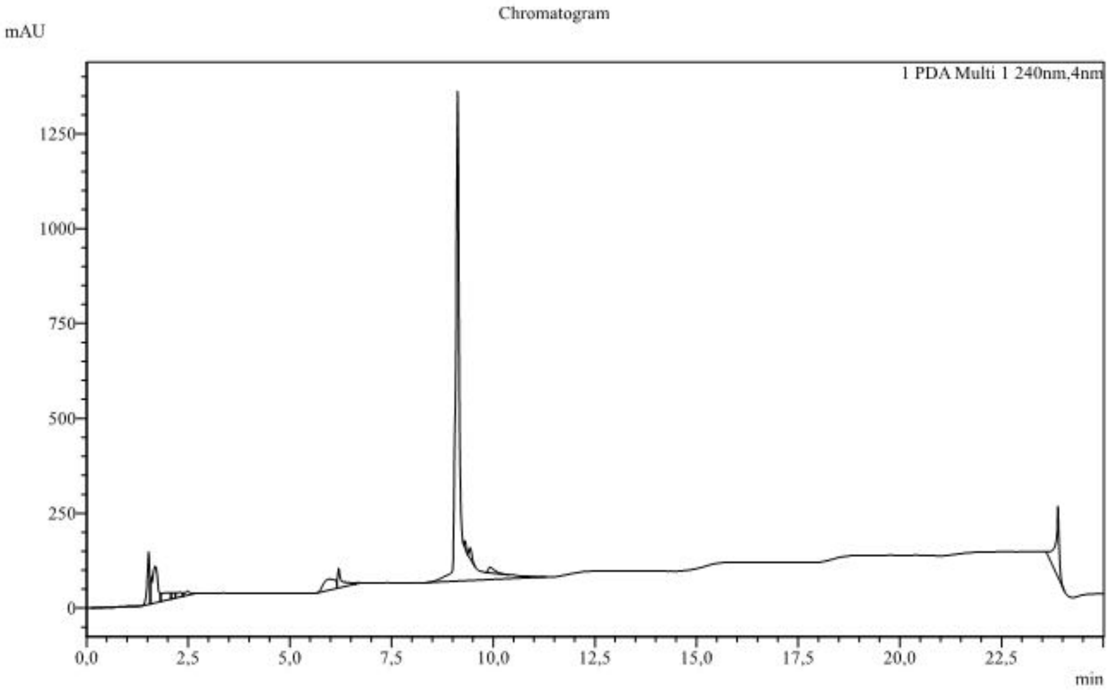

Figure S55. Chromatogram of compound **9**. HPLC: *Shimadzu Nexera XR,* Autosampler *SIL-20A*, diode array detector *SPD-M20A*, C18-column (150 × 4 mm). Method: 40% MeCN in H_2O + 0.1% HCOOH → 60% MeCN in H_2O + 0.1% HCOOH → 70% MeCN in H_2O + 0.1% HCOOH → 80% MeCN in H_2O + 0.1% HCOOH → 90% MeCN in H_2O + 0.1% HCOOH → 95% MeCN in H_2O + 0.1% HCOOH → 97% MeCN in H_2O + 0.1% HCOOH, flow: 1.0 mL/min.

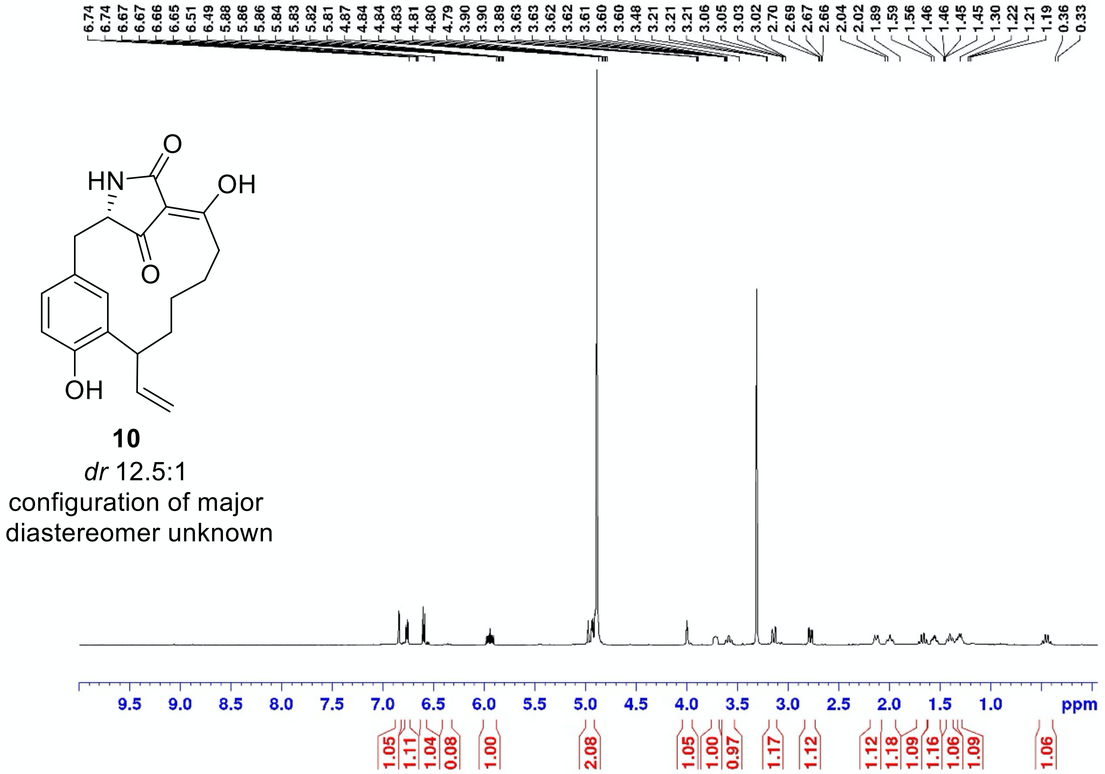

Figure S56. ^{1}H-NMR spectrum of compound **10** in CD_3OD.

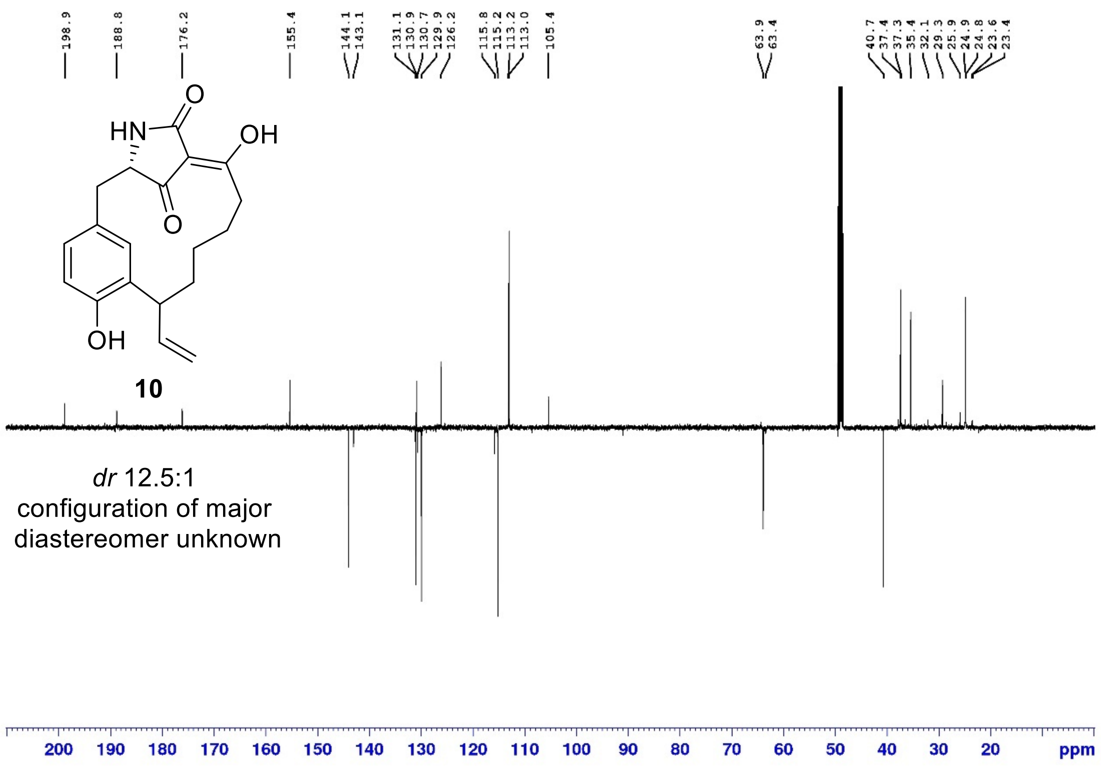

Figure S57. ^{13}C-NMR spectrum of compound **10** in CD_3OD.

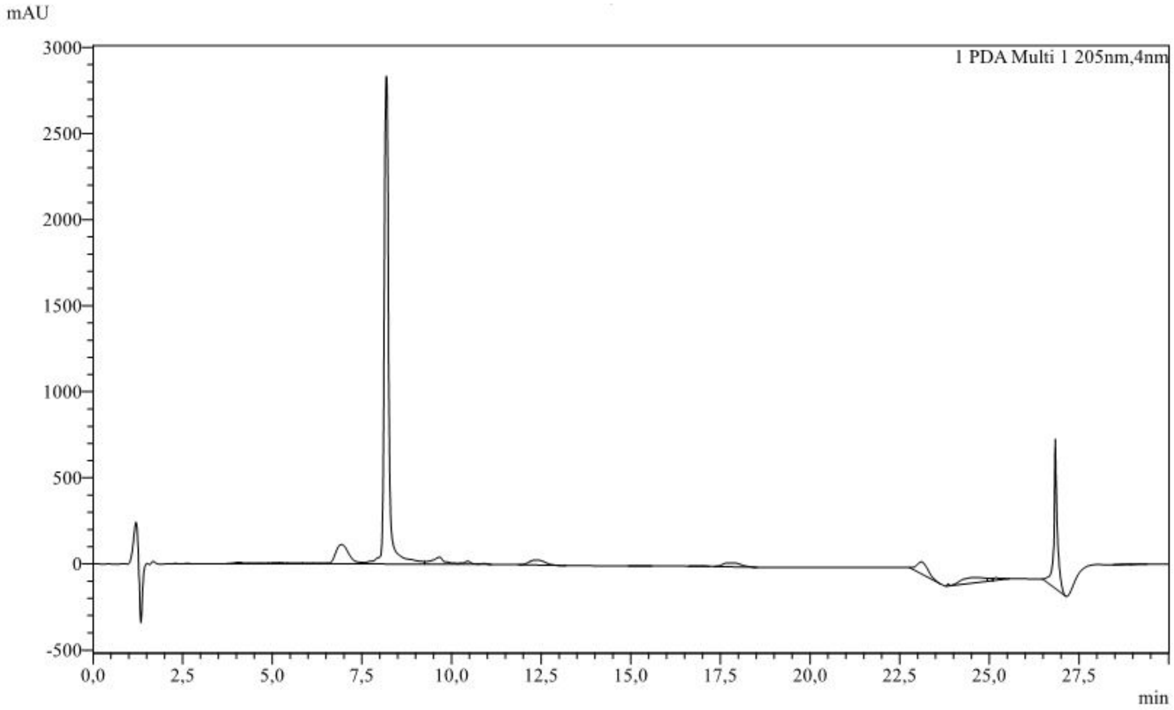

Figure S58. Chromatogram of compound **10**. HPLC: *Shimadzu Nexera XR,* Autosampler *SIL-20A*, diode array detector *SPD-M20A*, C18-column (150 × 4 mm). Method: 40% MeCN in H_2O + 0.1% HCOOH → 50% MeCN in H_2O + 0.1% HCOOH → 55% MeCN in H_2O + 0.1% HCOOH → 60% MeCN in H_2O + 0.1% HCOOH → 97% MeCN in H_2O + 0.1% HCOOH, flow: 1.0 mL/min.

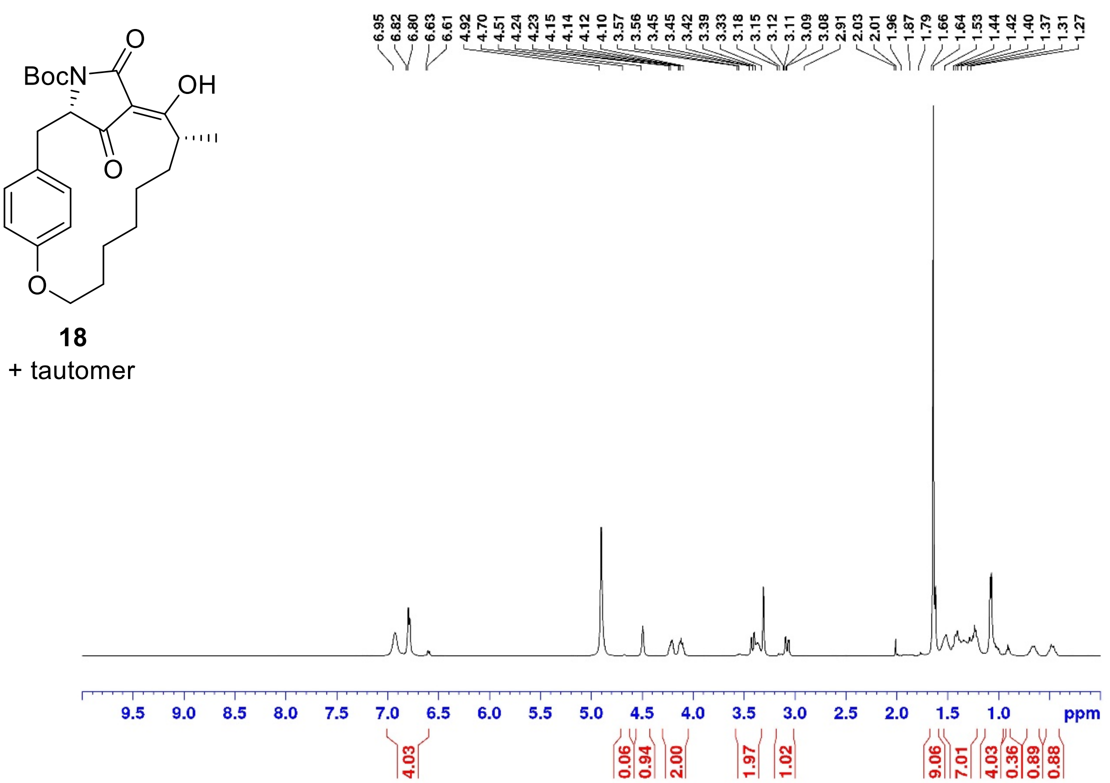

Figure S59. ^{1}H-NMR spectrum of compound **18** in CD_3OD.

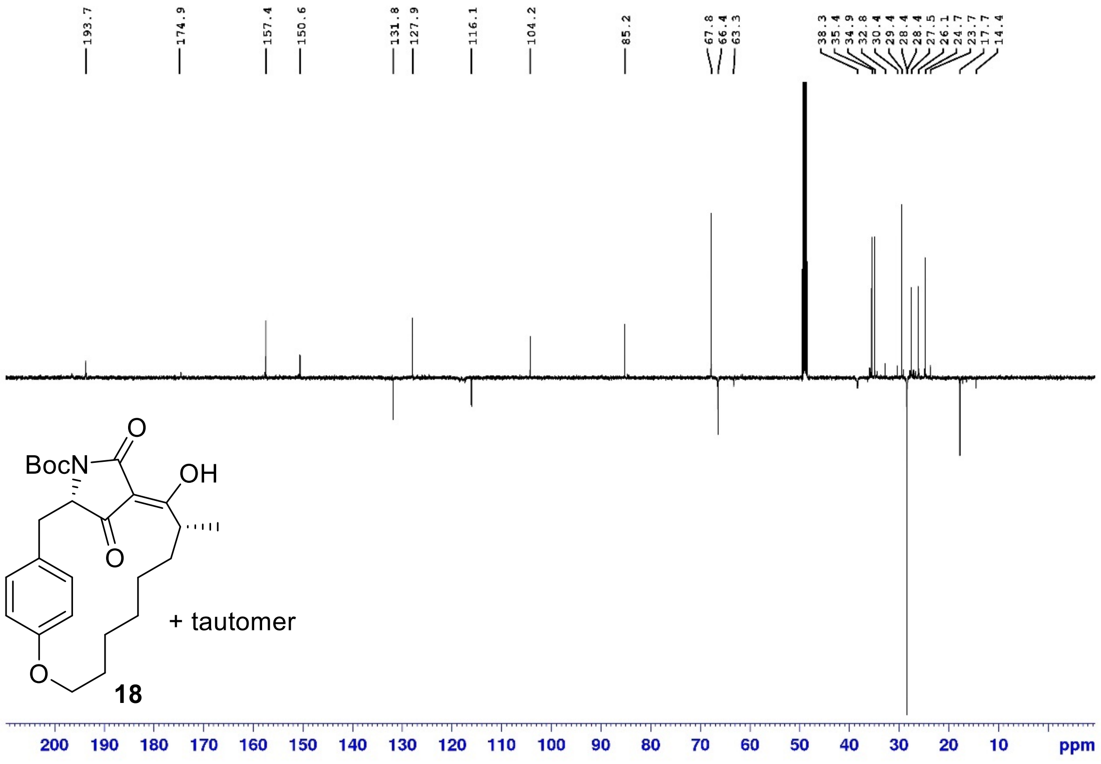

Figure S60. ^{13}C-NMR spectrum of compound **18** in CD_3OD.

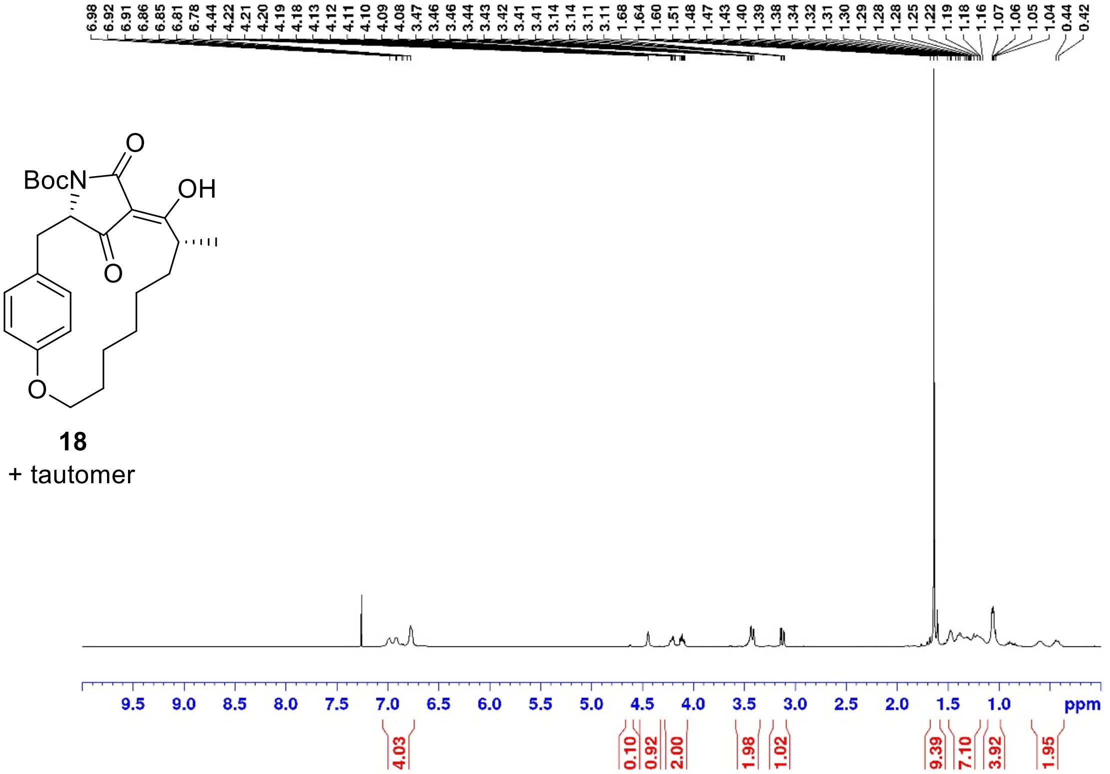

Figure S61. ^{1}H-NMR spectrum of compound **18** in $CDCl_3$.

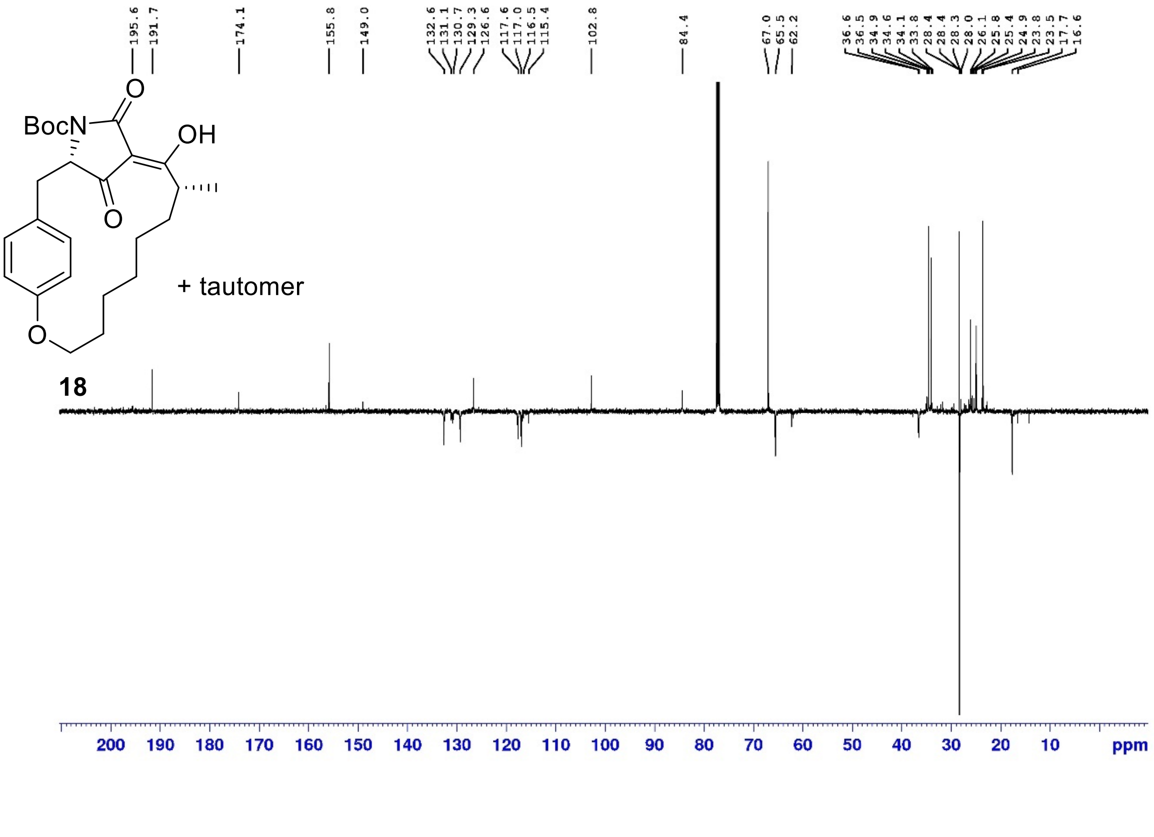

Figure S62. ^{13}C-NMR spectrum of compound **18** in $CDCl_3$.

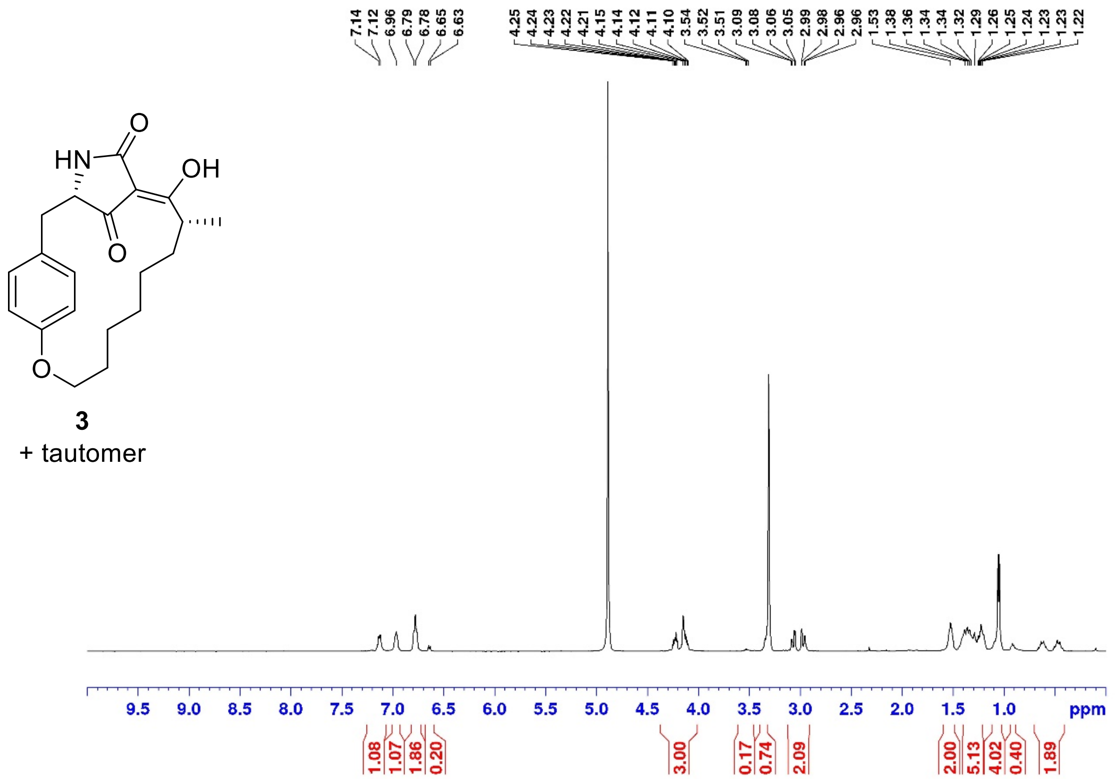

Figure S63. ^{1}H-NMR spectrum of compound **3** in CD_3OD.

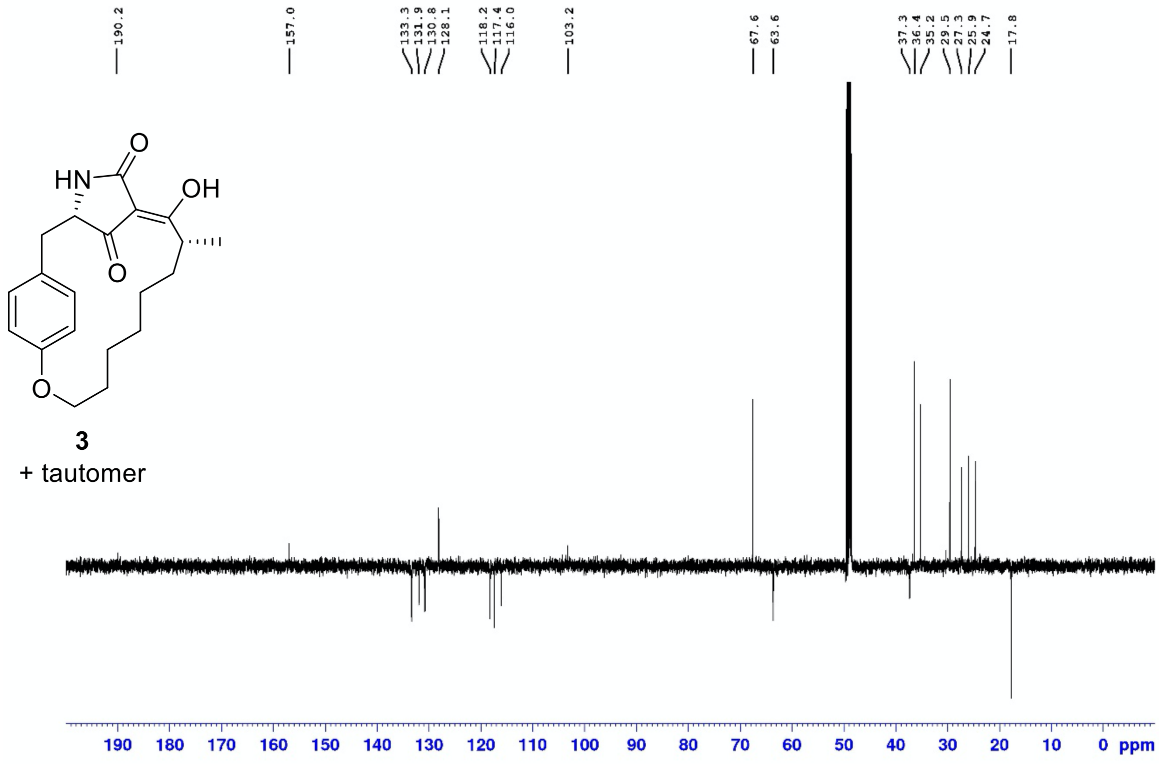

Figure S64. ^{13}C-NMR spectrum of compound **3** in CD_3OD.

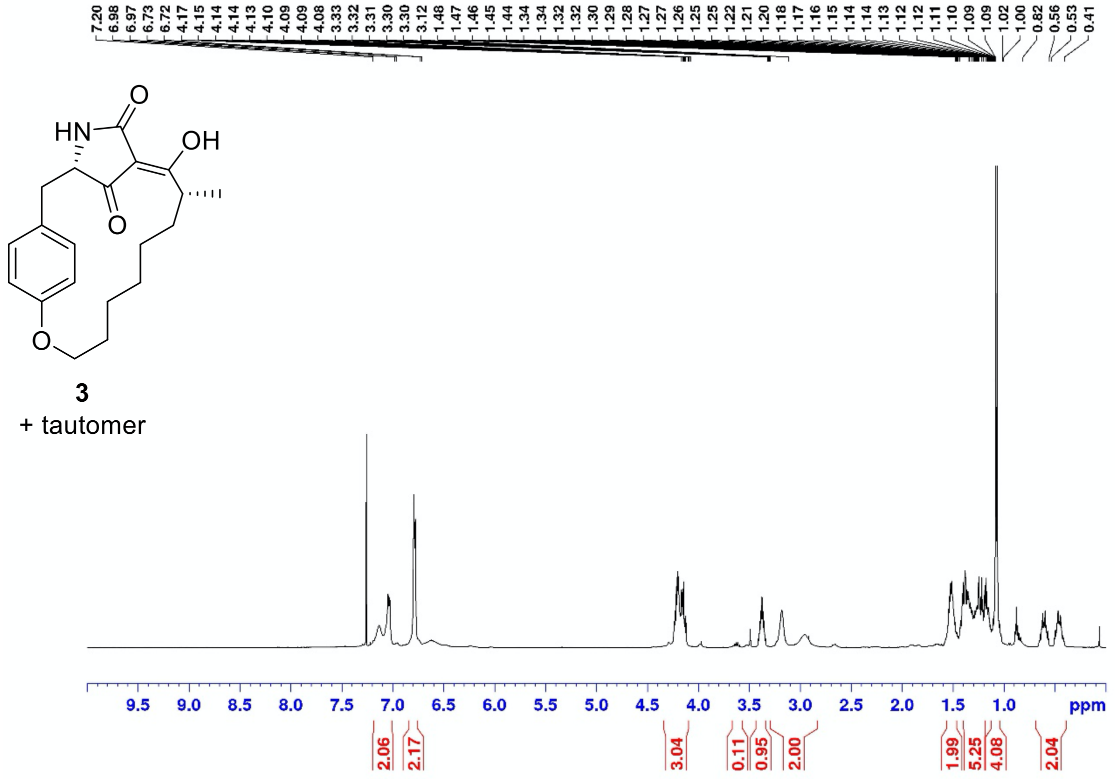

Figure S65. ^{1}H-NMR spectrum of compound **3** in $CDCl_3$.

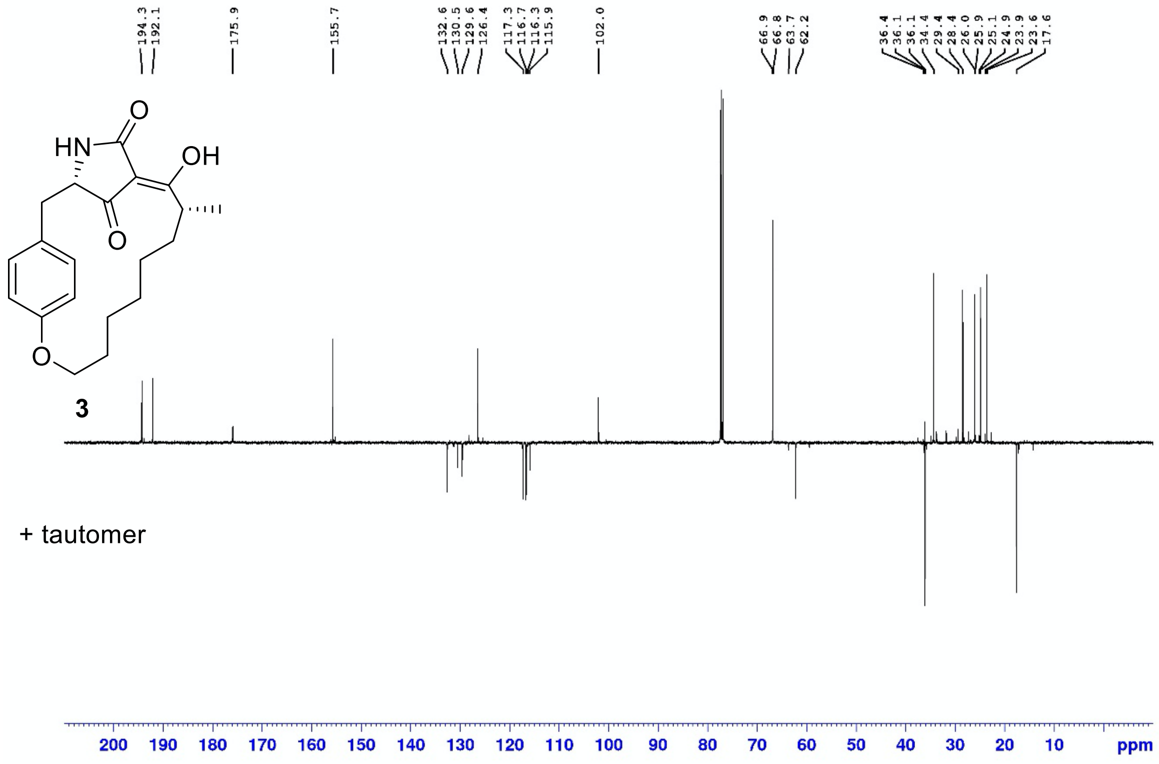

Figure S66. ^{13}C-NMR spectrum of compound **3** in $CDCl_3$.

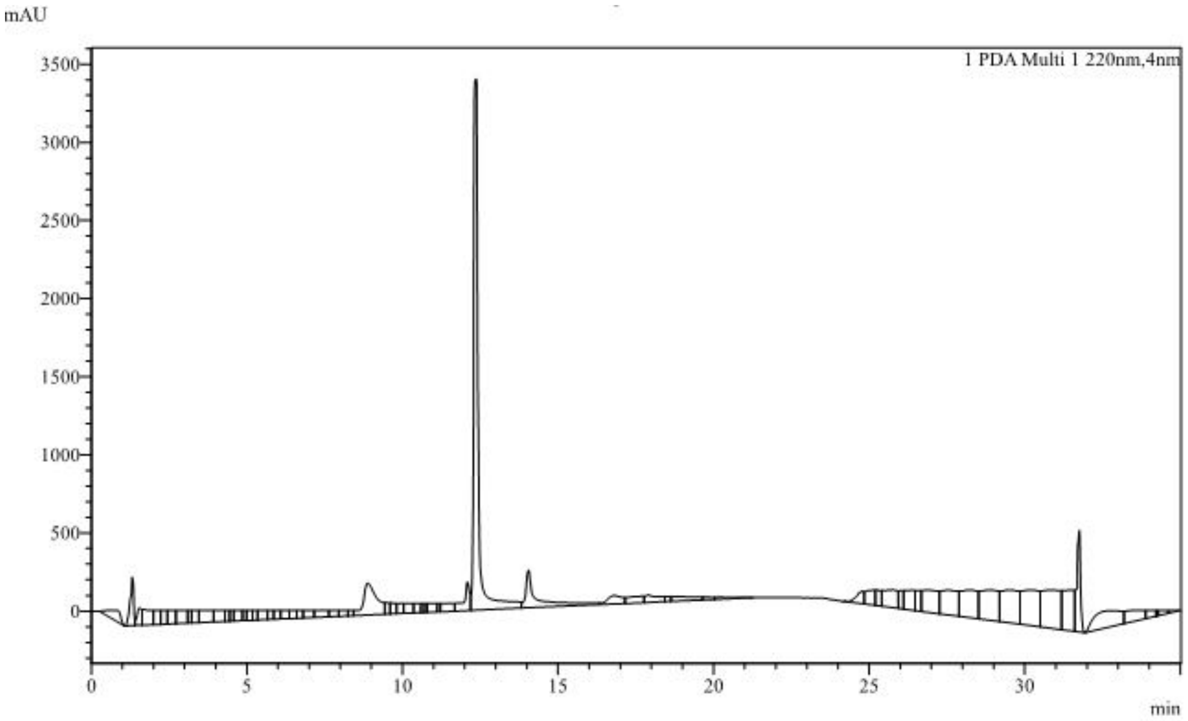

Figure S67. Chromatogram of compound **3**. HPLC: *Shimadzu Nexera XR,* Autosampler *SIL-20A*, diode array detector *SPD-M20A*, C18-column (150 × 4 mm). Method: 40% MeCN in H_2O + 0.1% HCOOH → 60% MeCN in H_2O + 0.1% HCOOH → 80% MeCN in H_2O + 0.1% HCOOH → 97% MeCN in H_2O + 0.1% HCOOH, flow: 1.0 mL/min.

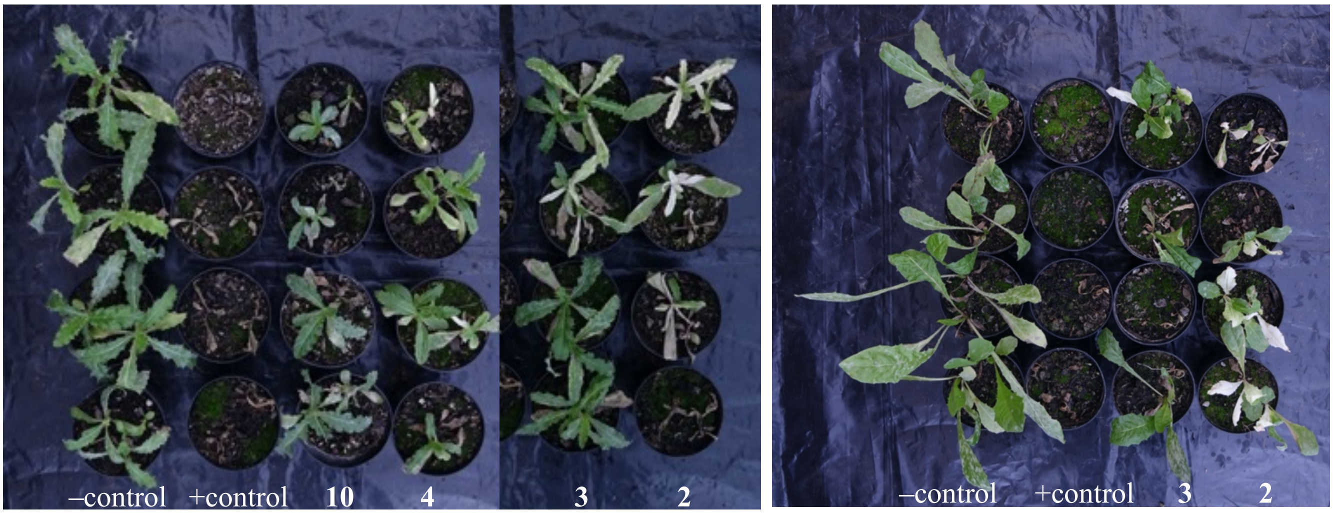

Figure S68: Thistles (left) and dandelions (right), 28 d after treatment with compounds **10** (150 mM), **4** (150 mM), **3** (100 mM) and **2** (100 mM). Negative control: isopropanol:water = 1:1 + 0.25% Tween20; positive control: commercial herbicide diflufenican (1.2 mM).

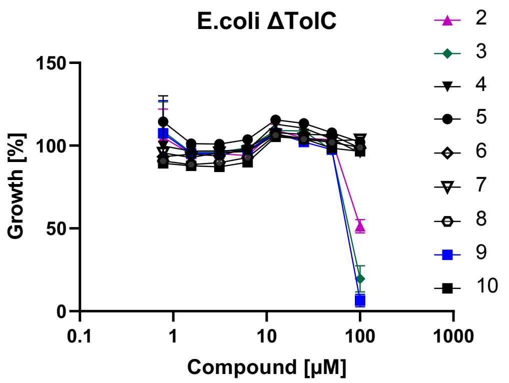

Figure S69. Growth inhibitory effects of various concentrations of macrocidin derivatives on *E. coli ΔTolC* cultures.

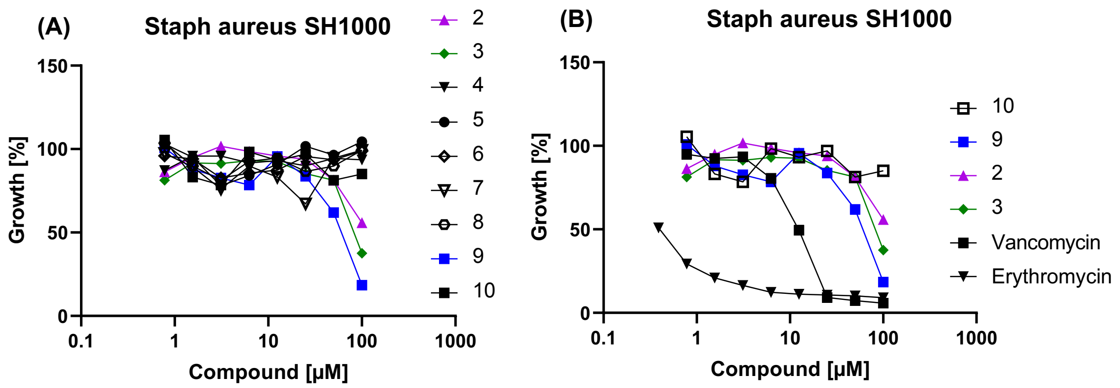

Figure S70. Growth inhibitory effects of various concentrations of (A) all macrocidinoids and of (B) the most active macrocidin derivatives **2**, **3** and **9**, and vancomycin and erythromycin on *S. aureus* (SH1000) cultures.

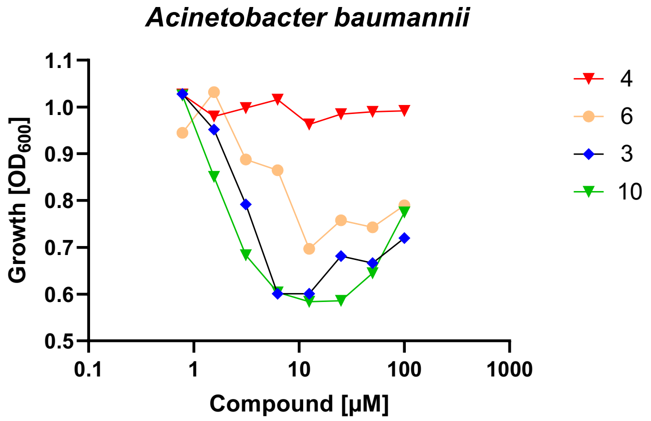

Figure S71. Growth inhibitory effects of various concentrations of selected macrocidin derivatives on *Acinetobacter baumannii* cultures.

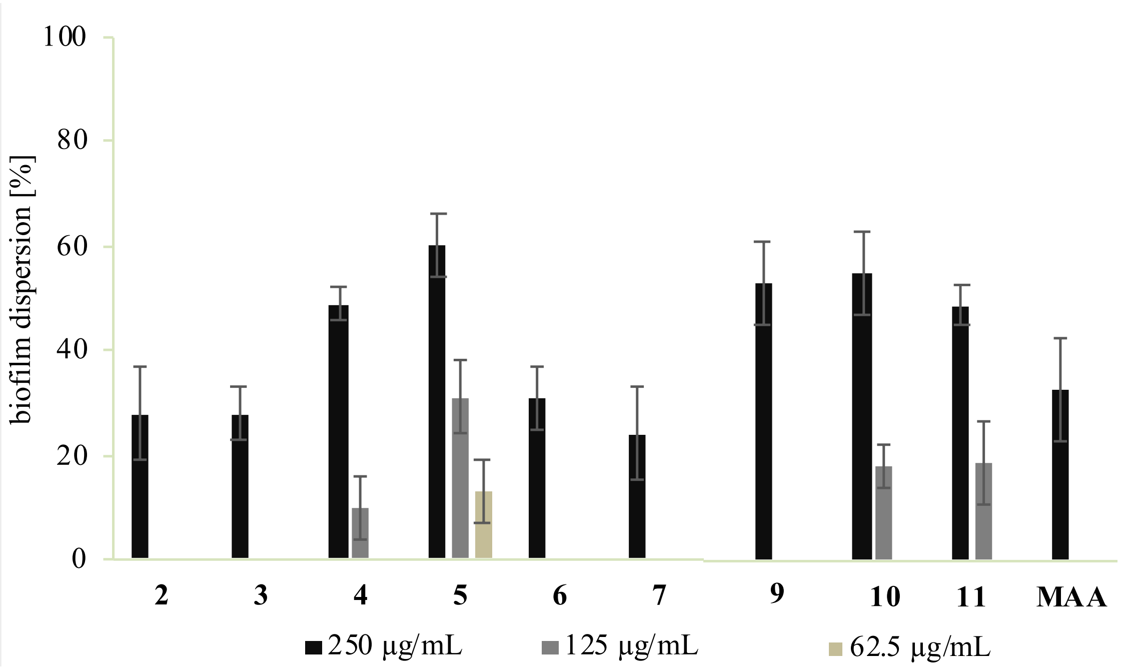

Figure S72. Dispersal effects on preformed biofilms of *C. albicans* by compounds **2-7** and **9-11** at various concentrations, error bars indicate SD.

Table S1. Antibacterial effects of compounds **2-10** on *E. coli* ΔTolC and *S. aureus*.
Inhibitory concentrations IC_{50} [µM] as determined by the broth microdilution method [ref. 21 and section 4. Materials and methods].

**Compound**	***Escherichia coli ΔTolC*** [µM]	***Staphylococcus aureus SH1000*** [µM]
**2**	100	100 ± 20
**3**	82 ± 15	83 ± 20
**4**	inactive	inactive
**5**	inactive	inactive
**6**	inactive	inactive
**7**	inactive	inactive
**8**	inactive	inactive
**9**	75 ± 15	57 ± 20
**10**	inactive	inactive
Vancomycin		12 ± 2

Table S2. Effects of compounds **2-10** on the formation of *S. aureus* biofilms and their dispersive effects on preformed biofilms of *S. aureus* and *C. albicans*, SD values are shown in brackets.

compounds	organisms	Biofilm inhibition / dispersion effects [%]								
		250 µg/mL	125 µg/mL	62.5 µg/mL	31.3 µg/mL	15.6 µg/mL	7.8 µg/mL	3.9 µg/mL	2 µg/mL	1.3 µg/mL
	S. aureus	85 (±3)	84 (±3)	84 (±2)	82 (±1)	74 (±1)	69 (±2)	56 (±4)	30 (±5)	30 (±10)
**2**	**preformed *S. aureus***	82 (±4)	85 (±3)	82 (±3)	74 (±8)	49 (±9)	30 (±5)	/	/	/
	**preformed *C. albican*s**	28 (±9)	/	/	/	/	/	/	/	/
	**S. aureus**	83 (±1)	79 (±3)	80 (±3)	80 (±3)	81 (±2)	74 (±2)	53 (±6)	36 (±9)	34 (±9)
**3**	**preformed S. aureus**	73 (±4)	75 (±3)	70 (±6)	63±14	35 (±12)	16 (±11)	/	/	/
	**preformed *C. albicans***	28 (±5)	/	/	/	/	/	/	/	/
	S. aureus	83 (±1)	85 (±1)	84 (±3)	77 (±4)	58 (±7)	31 (±10)	14 (±9)	13 (±7)	/
**4**	**preformed *S .aureus***	76 (±7)	68 (±12)	49 (±13)	14 (±10)	/	/	/	/	/
	**preformed *C. albicans***	49 (±3)	10 (±6)	/	/	/	/	/	/	/
	S. aureus	82 (±3)	81 (±4)	77 (±3)	65 (±10)	35 (±10)	15 (±10)	/	/	/
**5**	**preformed *S. aureus***	77 (±8)	64 (±13)	36 (±12)	23 (±2)	/	/	/	/	/
	**preformed *C. albicans***	60±6	31 (±7)	13 (±6)	/	/	/	/	/	/
	S. aureus	82 (±3)	56 (±3)	17 (±8)	/	/	/	/	/	/
**6**	**preformed *S. aureus***	25 (±11)	/	/	/	/	/	/	/	/
	**preformed *C. albicans***	31 (±6)	/	/	/	/	/	/	/	/
	S. aureus	/	/	/	/	/	/	/	/	/
**7**	**preformed *S. aureus***	/	/	/	/	/	/	/	/	/
	**preformed *C. albicans***	/	/	/	/	/	/	/	/	/
	S. aureus	76 (±10)	28 (±10)	/	/	/	/	/	/	/
**8**	**preformed *S. aureus***	/	/	/	/	/	/	/	/	/
	**preformed *C. albicans***	53 (±8)	/	/	/	/	/	/	/	/
	S. aureus	79 (±4)	81 (±3)	79 (±3)	79 (±6)	82 (±3)	68 (±7)	42 (±7)	35 (±9)	24 (±10)
**9**	**preformed *S. aureus***	73 (±9)	80 (±4)	79 (±4)	80 (±4)	57 (±3)	12 (±10)	/	/	/
	**preformed *C. albicans***	49 (±4)	19 (±8)	/	/	/	/	/	/	/
	S. aureus	82 (±3)	81 (±4)	79 (±3)	53 (±7)	33 (±8)	/	/	/	/
**10**	**preformed *S. aureus***	83 (±4)	79 (±4)	58 (±15)	33 (±12)	/	/	/	/	/
	**preformed *C. albicans***	/	/	/	/	/	/	/	/	/
	S. aureus	83 (±3)	82 (±4)	82 (±5)	80 (±4)	81 (±4)	77 (±6)	40 (±9)	/	/
**MAA**	**preformed *S. aureus***	68 (±2)	59 (±12)	50 (±4)	58 (±8)	/	/	/	/	/
	**preformed *C. albicans***	33 (±10)	/	/	/	/	/	/	/	/
		**(/) no activity**								

Table S3. Inhibitory concentrations IC_{50} [µM] of compounds **2-10** when applied to KBV cervix carcinoma, 518A2 melanoma, HCT-116, HCT-116$^{p53-/-}$ knockout mutant colon carcinoma and EaHy hybrid endothelial cells. Compounds were tested in a range from 50 nM to 100 µM. Values are the means ± SD determined in four independent experiments and derived from dose-response curves after 72 h incubation using the MTT assay. [n.d. = not determined]

	**IC_{50} values [µM]**				
	**518A2**	**HCT-116^{wt}**	**HCT-116^{p53-}**	**EaHy**	**KbV**
**2**	>50	13.9±0.8	17.2±2	n.d.	31.3±3
**3**	n.d.	>50	>50	n.d.	>50
**6**	n.d.	>50	>50	>50	>50
**7**	>50	>50	>50	>50	>50
**8**	n.d.	>50	>50	>50	>50
**9**	n.d.	>50	>50	>50	>50
**10**	n.d.	>50	>50	>50	>50
